# Self-organized patterning of crocodile head scales by compressive folding

**DOI:** 10.1038/s41586-024-08268-1

**Published:** 2024-12-11

**Authors:** Gabriel N. Santos-Durán, Rory L. Cooper, Ebrahim Jahanbakhsh, Grigorii Timin, Michel C. Milinkovitch

**Affiliations:** 1 Laboratory of Artificial & Natural Evolution (LANE), Department of Genetics & Evolution, University of Geneva, Geneva, Switzerland; 2 SIB Swiss Institute of Bioinformatics, Geneva, Switzerland

**Keywords:** Pattern formation, Morphogenesis, Evolutionary developmental biology, Dynamical systems, Computational biophysics

## Abstract

Amniote integumentary appendages constitute a diverse group of micro-organs, including feathers, hair and scales. These structures typically develop as genetically controlled units^1^, the spatial patterning of which emerges from a self-organized chemical Turing system^2,3^ with integrated mechanical feedback^4,5^. The seemingly purely mechanical patterning of polygonal crocodile head scales provides an exception to this paradigm^6^. However, the nature and origin of the mechanical stress field driving this patterning remain unclear. Here, using precise in ovo intravenous injections of epidermal growth factor protein, we generate Nile crocodile embryos with substantially convoluted head skin, as well as hatchlings with smaller polygonal head scales resembling those of caimans. We then use light-sheet fluorescence microscopy to quantify embryonic tissue-layer geometry, collagen architecture and the spatial distribution of proliferating cells. Using these data, we build a phenomenological three-dimensional mechanical growth model that recapitulates both normal and experimentally modified patterning of crocodile head scales. Our experiments and numerical simulations demonstrate that crocodile head scales self-organize through compressive folding, originating from near-homogeneous skin growth with differential stiffness of the dermis versus the epidermis. Our experiments and theoretical morphospace analyses indicate that variation in embryonic growth and material properties of skin layers provides a simple evolutionary mechanism that produces a diversity of head-scale patterns among crocodilian species.

## Abstract (web summary)

Crocodile head scales self-organize through purely mechanical compressive skin folding rather than a patterning process controlled by gene interactions.

## Main

Vertebrates exhibit a diverse array of integumentary appendages, including feathers, hair and scales. These micro-organs facilitate various functions, ranging from mechanical protection and thermoregulation to sexual display. Previous research has demonstrated that the early embryonic development of diverse integumentary appendages is broadly conserved^1,7^. Typically, these units develop from anatomical placodes characterized by conserved molecular signalling in both the epidermis and the underlying dermis^1,8–11^. Indeed, the patterning of hair, feathers and scales crucially requires Turing reaction–diffusion-type dynamics^12–15^, produced by chemical interactions between activatory and inhibitory morphogens^2,3,16^. However, ex vivo studies have shown that the self-organized periodic patterning of integumentary appendages can also involve mechanical components^17^—for example, the local aggregation and contraction of mesenchymal cells activates feather primordia development through the mechanosensitive signalling of β-catenin^4,5^. In chicken, the nested spatial patches of morphogen expression in the dermis might also endow the corresponding tissue domains with different material properties, thereby generating the budding of feather primordia through an elastic instability^18^.

**Fig. 1:**
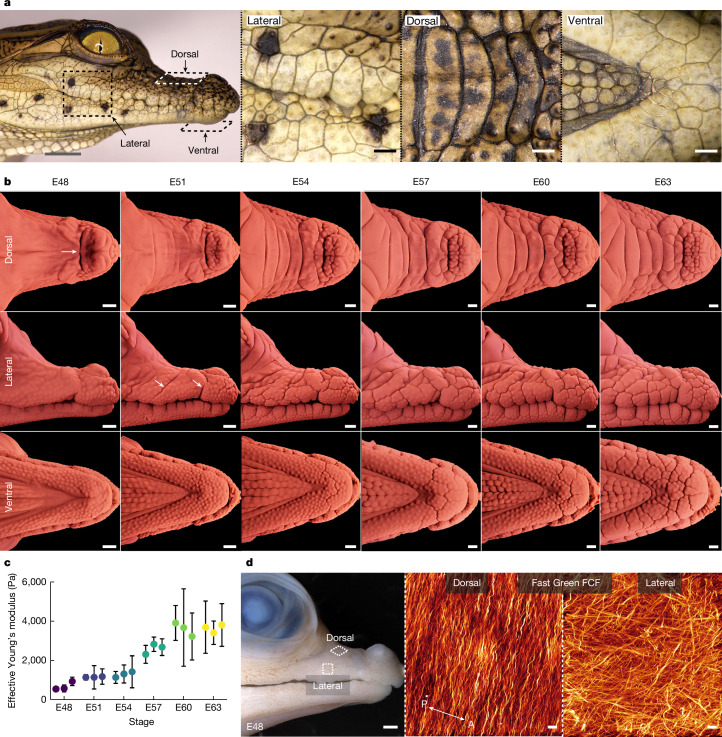
Dynamics of crocodile head-scale development. **a**, The scales adorning the upper and lower jaws of hatched Nile crocodiles (*C. niloticus*) form irregular, non-overlapping polygonal domains^6^. **b**, LSFM imaging of nuclear-stained (TO-PRO-3) samples reveals changes in the surface geometry of the upper and lower jaws during scale emergence. At E48, the head appears mostly smooth. Scale edges subsequently propagate across the skin surface until both the lower and upper jaws are covered by irregular scale domains by E63. The scales of the upper jaw dorsal surface (top row of images) are larger and more elongated than those observed elsewhere. **c**, Nanoindentation reveals that head-scale emergence is associated with an increase in epidermal surface stiffness from E48 to E63. *n* = 3 biologically independent samples per stage. Data are mean ± s.d. of 10 measurements per sample. **d**, Fast Green staining reveals the anisotropy of collagen architecture of the embryonic dermis at E48. The dorsal upper jaw dermis exhibits highly organized collagen fibres, running perpendicular to the long axis (anteroposterior, A–P) of the jaw, whereas fibres of the lateral jaw dermis lack a single dominant direction. This result was validated in *n* = 8 individual biological replicates using either LSFM or confocal microscopy. Scale bars, 10 μm (**d** (middle and right)), 5 mm (**a** (column 1)) and 1 mm (**a** (columns 2–4), **b** and **d** (left)).

Analyses of developing crocodile embryos have demonstrated that their head scales (that is, the scales covering their face and jaws), but not their body scales, constitute irregular non-overlapping convex polygonal domains of highly keratinized skin^6^ (Fig. 1a). Rather than emerging from placodes, spatially organized through paradigmatic chemical reaction–diffusion patterning^2,3,16^ and putative mechanical feedback, these head scales instead appear to arise from a purely mechanical process generating a pattern reminiscent of material cracking^6^. One speculative explanation for this superficial similarity was that skin cell proliferation might be strongly coupled to mechanical tension produced by the rapid growth of the crocodile embryonic jaw skeleton. More specifically, any local crease nucleated by tension-driven proliferation would cause tensile stress to redistribute and accumulate at its tips, causing the successive cracking-like propagation^19^ of local tensile stress, local proliferation maximum and crease tips. Given the great difficulties of experimentation with crocodile embryos, neither this nor alternative mechanical processes have been tested.

Here, using in vivo experiments and numerical simulations, we invalidate the tension-driven local growth hypothesis and reveal that the edges of crocodile head scales are inward skin folds generated by in-plane compressive stress originating from rapid near-homogeneous growth of the skin. First, we treat developing embryonic Nile crocodiles (*Crocodylus niloticus*) with in ovo intravenous injections^20,21^ of epidermal growth factor protein (EGF) to exacerbate epidermal differentiation^22,23^ (and, therefore, its effective stiffness) and growth, thereby perturbing the mechanics underlying head-scale formation. While quantifying these results with light-sheet fluorescence microscopy (LSFM), we show that this treatment results in crocodile embryos with substantially convoluted head skin patterns. By arresting this treatment at the appropriate embryonic stage, this ‘brainy’ network of skin folds partially relaxes towards a pattern of smaller polygonal head scales in hatched crocodiles, that is, highly similar to the head-scale patterns of caimans. Next, we validate all our experimental results with extensive numerical simulations implementing a three-dimensional (3D) mechanical growth model that incorporates parameters inferred from volumetric LSFM. Notably, the normal Nile crocodile head-scale patterning process requires differential stiffness of the dermis versus the epidermis, but does not require differential growth of these two adherent skin layers. Finally, we produce a theoretical morphospace of skin folding patterns showing that variations of both growth and material properties of the dermis versus the epidermis readily explain the diversity of head-scale patterns among crocodilian species.

## Mechanical patterning of head scales

**Extended Data Fig. 1:**
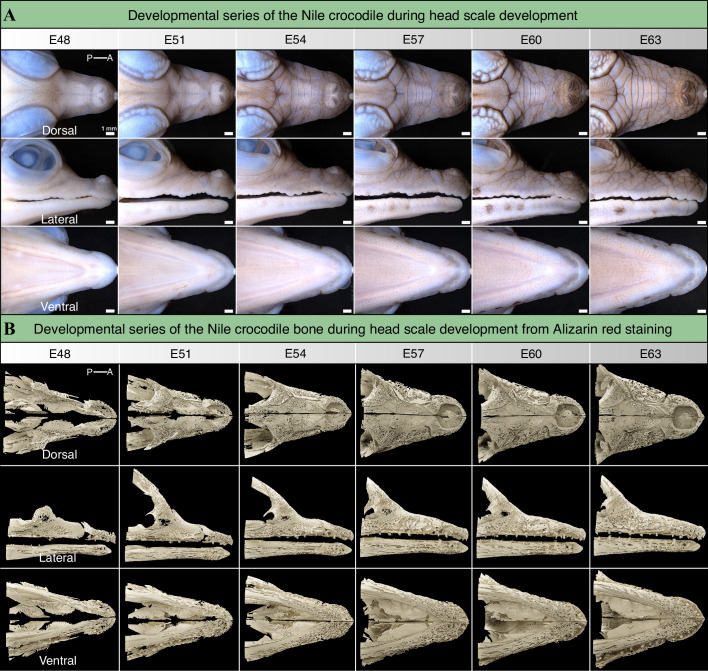
Normal embryonic development of the crocodile head. (**A**) Growth series (stereomicroscope views of dorsal, lateral and ventral sides) showing the normal emergence of crocodile head scales, obtained from embryonic Nile crocodile samples fixed between E48 and E63. These samples are equivalent to those comprising the LSFM growth series in Fig. 1b and Supplementary Video 1. (**B**) Growth series showing progressing ossification from E48 to E63 of the upper jaw and lower jaw bones of embryonic Nile crocodiles stained with Alizarin Red. 3D images are acquired with LSFM and are also shown in Supplementary Video 2. P = posterior; A = anterior.

First, we investigate the normal patterning of head scales in the Nile crocodile between embryonic days 48 (E48) and E63 (Fig. 1b, Extended Data Fig. 1a and Supplementary Video 1). At E48, the elongated jaws appear mostly smooth (Fig. 1b (left)), besides the presence of the nasal disc on the upper jaw (white arrow) and placode-derived integumentary multi-sensory organs (ISOs) scattered across both jaws^6,24^. However, by E51, the first skin folds propagate across the laterodorsal surface of the upper jaw (Fig. 1b (second column, white arrows)). As the embryo continues to develop, new edges arise, propagate and interconnect to form polygonal domains, until the face and jaws are covered with irregular, non-overlapping scales by E63 (Fig. 1b (right column)). Nucleation and propagation of new folds rapidly decreases beyond E63 and is completed by E75 (ref. ^6^).

Large, elongated head-scale domains oriented perpendicular to the jaw’s long axis are visible on the dorsal surface of the upper jaw, whereas smaller, more regular, polygonal domains appear on the lateral sides of both jaws (Fig. 1b, Extended Data Fig. 1a and Supplementary Video 1). Head-scale emergence is accompanied by the progression of ossification of the underlying bone and the development of teeth (Extended Data Fig. 1b and Supplementary Video 2). Nanoindentation of the lateral upper jaw surface reveals that the emergence of crocodile head scales is associated with progressively increased stiffness of the epidermal surface from E48 to E63 (Fig. 1c), which is indicative of increased tissue density and keratinization. Note that the propagating scale edges avoid the ISOs. Optical LSFM sections confirm that ISOs, which develop before the onset of skin folding^6^, comprise dermal cell condensations and associated innervation^24^ within a collagen-rich dermis^25^, beneath a dense epidermal cell layer (Supplementary Fig. 1).

Collagen is an abundant structural protein of the extracellular matrix and is a key determinant of tissue-specific material properties^26^. Our high-resolution confocal imaging of the crocodile upper jaw at E48, before the emergence of head scales, reveals that the dorsal upper jaw dermis exhibits highly organized collagen fibres running perpendicular to the jaw long axis (Fig. 1d and Supplementary Fig. 2), that is, in the same direction as the elongated scales that will subsequently emerge in the same region (Fig. 1a,b). In comparison, fibres of the lateral upper jaw dermis are more disorganized and lack a single dominant orientation. We hypothesize that such variation in the 3D organization of collagen fibres, and the resultant discrepancy in the material properties of the dermis, contributes to differences in head-scale geometries observed between the dorsal and lateral upper jaw surfaces.

The presence of both incomplete edges and a substantial number of 90° edge junctions in the crocodile head-scale lattice is compatible with a developmental process analogous to material cracking in a tensile stress field^6^. Such a process would require tensile stress to locally enhance proliferation, causing the nucleation and propagation of both the stress and proliferation maxima, possibly generating dynamics similar to those observed in material cracking^6^. However, labelling of proliferating cells with 5-ethynyl-2′-deoxyuridine (EdU) (Supplementary Video 3) does not evidence increased proliferation at the tips of propagating folds (Supplementary Fig. 3), invalidating the tension-driven local growth hypothesis.

## EGF agonism modifies scale patterning

**Fig. 2:**
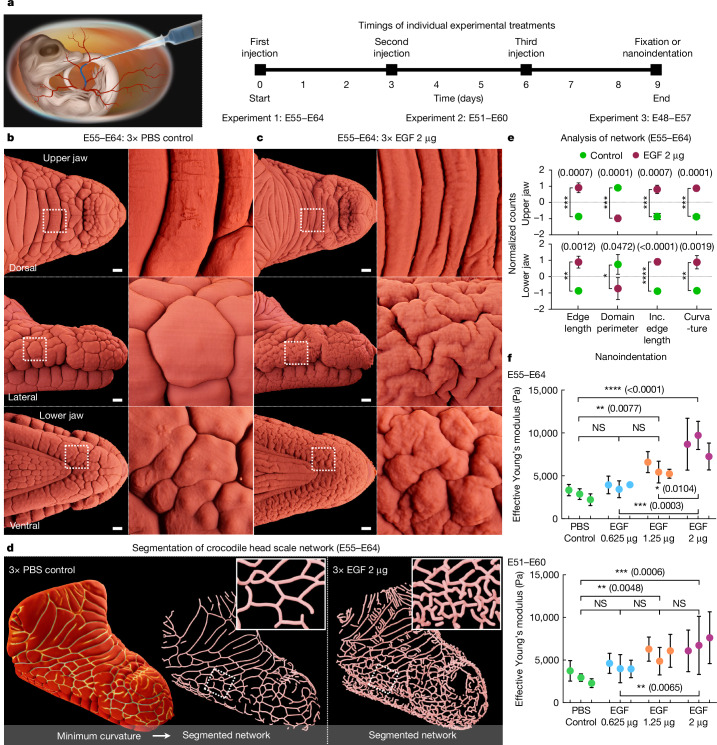
Agonism of epidermal growth substantially modifies crocodile head-scale patterning. **a**, Crocodile embryos were treated with repeated intravenous injections of EGF at different stages of head-scale development. **b**, Control embryos injected with PBS exhibit normal patterning of polygonal head-scale domains (LSFM imaging of TO-PRO-3 nuclear staining). **c**, Embryos injected with 2 µg EGF exhibit considerably modified folding on both the upper and lower jaws. **d**,**e**, Curvature-based segmentation of the fold network (**d**) was used to quantify geometrical and topological features (**e**) that distinguish control versus EGF-treated samples. *n* ≥ 4 biological replicates per treatment. Data are mean  ± s.d. Inc. edge length, length of incomplete edges, that is, connected only at one end. **f**, Nanoindentation of the lateral upper jaw surface reveals a significant dose-dependent increase in epidermal surface stiffness after EGF treatment. *n* = 3 biologically independent samples per treatment group. Data are mean ± s.d. of five measurements per sample. Statistical significance was calculated using one-way analysis of variance (ANOVA) (**e** and **f**); NS, not significant; *P < 0.05, **P < 0.01, ***P < 0.001, ****P < 0.0001. Individual *P* values are shown in parentheses. Scale bars, 1 mm (**b** and **c**).

**Extended Data Fig. 2:**
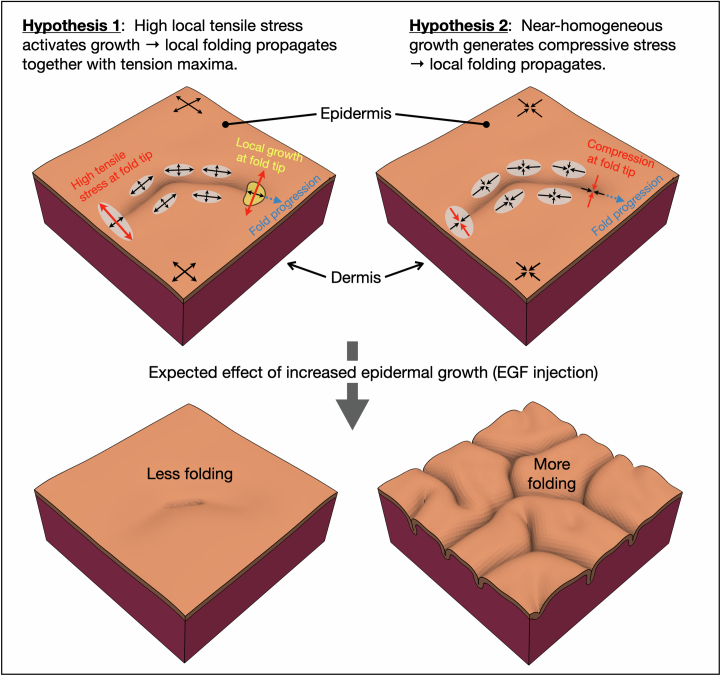
Comparison of the tension-driven (cracking-like) local growth and compression-driven hypotheses. **(Top left)** Hypothesis 1 assumes that *(i)* the skin is under tension because of the rapid growth of the crocodile bony jaws and *(ii)* cell proliferation (hence, skin growth) is strongly activated beyond a given threshold of tensile stress. Under these assumptions, any crease nucleated by local tension-driven proliferation would cause the initial tensile stress perpendicular to the crease to redistribute and accumulate at its tips. Hence, the large tensile stress at fold tips might cause the propagation of the local proliferation maximum, hence, the cracking-like progression of the fold. As we do not observe a significantly larger density of EdU^+^ cells at the tips of propagating folds (Supplementary Fig. 3), hypothesis 1 can be rejected. **(Top right)** Hypothesis 2 assumes that the skin is growing faster than the underlying head tissues. Hence, the skin is under compressive stress, which is dissipated through folding. This hypothesis does not require the coupling of mechanical stress with cell proliferation. **(Bottom)** As EGF exacerbates growth, treatment of developing embryos with this drug would reduce or increase folding under hypothesis 1 or hypothesis 2, respectively. Note that, under both hypotheses, folds would tend to connect at 90° with pre-existing skin folds (or domain boundaries) because it would propagate perpendicular to the local tensile or compression maxima, respectively.

Conversely, the hierarchical development of scales on the face and jaws of crocodiles could be caused by an alternative, purely mechanical, self-organized process: the emergence of compressive elastic instabilities^27,28^, in the form of folds, caused by differential homogeneous growth between the dermis and epidermis or between the skin and underlying stiff tissues. Comparable mechanisms have been attributed to the surface buckling of tumours^29^, the developmental patterning of cortical convolutions of the human brain^30^, villification of the human and chicken gut^31^, and the surface wrinkling of mucosa^32^. To test this hypothesis, we treated developing crocodile embryos in vivo with precise and repeated intravenous injections^21^ of EGF (Fig. 2a) to experimentally elevate global epidermal growth and differentiation (here, keratinization) during head-scale emergence. This treatment enables us to further distinguish between the tension-field (cracking-like) hypothesis and the compression-field hypothesis (Extended Data Fig. 2). Indeed, the experimentally exacerbated global epidermal growth caused by EGF treatment would counteract a putative tension field and therefore reduce folding. By contrast, if head-scale patterning is caused by a compression field, EGF treatment would intensify compression and, therefore, increase folding.

We tested multiple combinations of both EGF doses and experimental timings (Supplementary Table 1 and Supplementary Fig. 4). In each experiment, individual embryos were injected three times (on experimental days 0, 3 and 6), before fixation (or nanoindentation) on day 9 (Fig. 2a). After post-treatment fixation, the samples were processed for LSFM to precisely quantify modifications to tissue folding geometry resulting from EGF treatment (Methods). Crocodile embryos treated from E55 to E64 with control injections of PBS exhibit normal patterning of polygonal head-scale domains (Fig. 2b and Supplementary Video 4 (left)). Conversely, when treated with injections of 2 µg EGF, embryos develop extensive additional skin folding on both the upper and lower jaws (Fig. 2c and Supplementary Video 4 (right)), thereby invalidating the tension-field hypothesis and validating the compression-field hypothesis. More specifically, EGF-treated embryos exhibit thinner and more numerous elongated scale domains on the dorsal surface of the upper jaw, and brainy (that is, labyrinthine) folding patterns on the lateral jaw surfaces.

**Extended Data Fig. 3:**
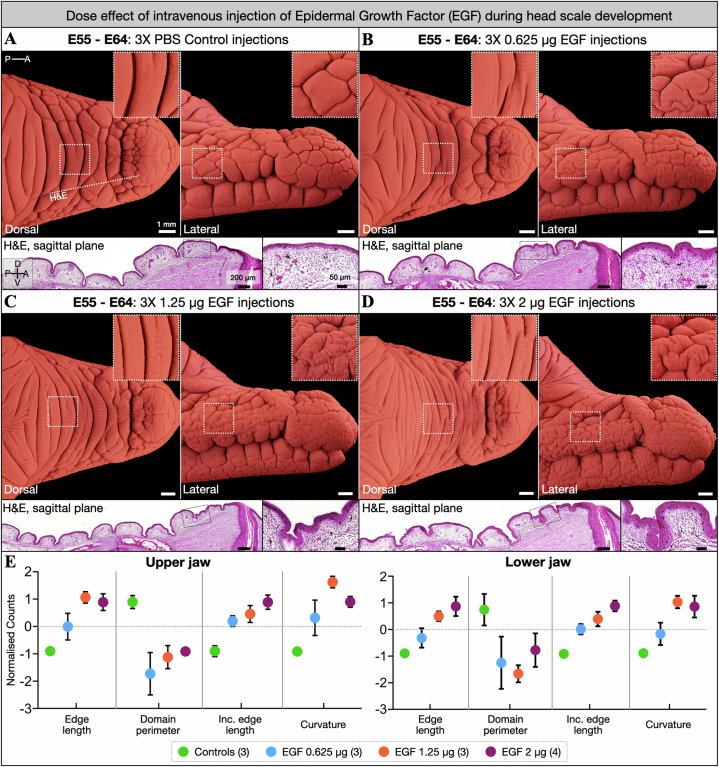
The effect of EGF treatment on head-scale patterning is dose-dependent — geometrical analysis. Surface rendering of 3D volumes acquired with LSFM (TO-PRO-3 Iodide nuclear staining). The dose effect of EGF treatment is shown for samples treated from E55 to E64. **(A)** Control embryos injected with PBS reveal normal patterning of head scales. The A-P and D-V axes show the antero-posterior and the dorso-ventral orientations, respectively. **(B-D)** Increasing the dose of EGF treatment results in increased additional folding on both the upper and lower jaws, as well as substantially increased epidermal thickness (revealed by H&E sections; see also Supplementary Fig. 5). **(E)** The dose effect of EGF treatment is quantified with geometrical and topological features (see Methods) of the corresponding folding networks (number of individuals is indicated between parentheses: n = 3 biologically independent samples per treatment except for 2 µg EGF for which n = 4; error bars show standard deviations). The high value of ‘domain perimeter’ in the control samples is due to the dominance of large polygonal domains in the natural folding network.

**Extended Data Fig. 4:**
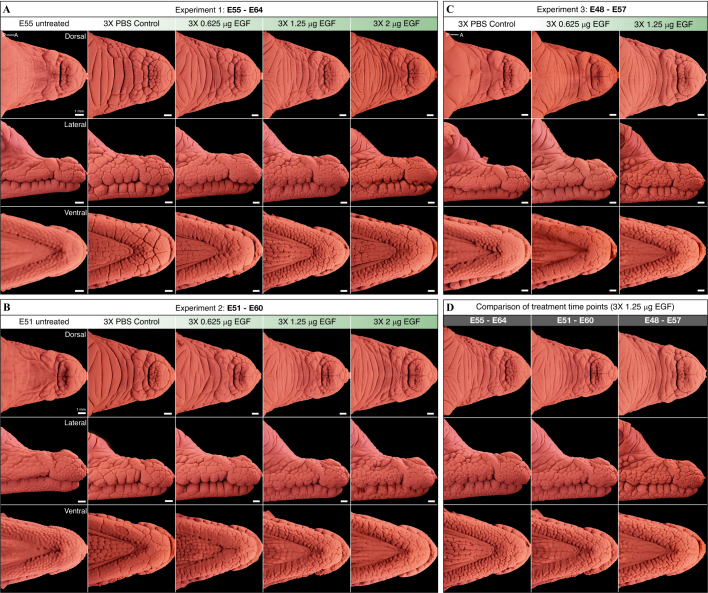
EGF treatment is consistently EGF-dose dependent for different treatment time points. Surface rendering of 3D volumes acquired with LSFM (TO-PRO-3 Iodide nuclear staining) shows the effect of experimental timing of treatment upon skin surface folding. Samples treated at different experimental time points all exhibit a dose-dependent increase in skin surface folding associated with EGF treatment, including **(A)** samples treated from E55 to E64, **(B)** samples treated from E51 to E60, and **(C)** samples treated from E48 to E57. **(D)** Corresponding differences in folding pattern length scales can be observed by directly comparing embryos treated with the same EGF dose (3×1.25 µg EGF) at different treatment times. P = posterior; A = anterior.

**Extended Data Fig. 5:**
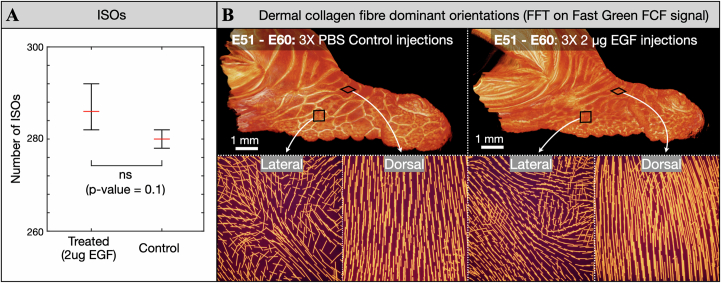
EGF treatment does not affect ISOs or collagen architecture. **(A)** The number of ISOs are similar in three control and three treated embryos at E64; an unsurprising observation given that ISOs develop well before the onset of skin folding. Note that ISOs below the plane defined by the egg tooth and the two (left and right) premaxillary notches were not counted because their segmentation from LSFM data is unreliable. (n = 3 biologically independent samples per treatment; the red line shows the median value and error bars show the minimum and maximum values). Statistical significance is calculated using one-way ANOVA. **(B)** High-resolution LSFM imaging (Fast Green FCF staining) indicates that both control and EGF-treated embryos fixed at E60 exhibit the same highly-organized collagen fibres on the top of the upper jaw (*i.e*., running perpendicular to the jaw long axis), whereas lateral jaw surface lacks a single consistent dominant orientation of fibres.

We next used curvature-based segmentation to detect folds (Fig. 2d) and we quantified geometrical and topological features (Methods) of the corresponding networks (Fig. 2e): these features strongly distinguish the controls from the EGF-treated samples. The effect of treatment is dose dependent, with higher EGF doses corresponding to increased epidermal growth and keratinization (Extended Data Fig. 3) as well as increased skin folding (Supplementary Fig. 5 and Supplementary Video 5). Furthermore, nanoindentation measurements after EGF treatment across two separate time periods confirm a dose-dependent increase in epidermal surface stiffness caused by hyperkeratinization (Fig. 2f). We also examined the effect of EGF treatment at different developmental timepoints (Fig. 2a). EGF injections beginning at either E51 or E48 also result in a substantial increase in tissue folding (Extended Data Fig. 4) and epidermal surface stiffness (Fig. 2f (bottom)). Note that EGF treatment does not appear to affect the development of ISOs (Extended Data Fig. 5a); an unsurprising observation given that ISOs develop well before the onset of skin folding^6,24^. Moreover, EGF-treated samples exhibit the same variation in the 3D organization of collagen fibres (dorsal upper jaw versus lateral jaw) as observed in control samples (Extended Data Fig. 5b).

Overall, our results demonstrate that experimentally increasing global epidermal growth and differentiation extensively modifies the folding patterns of crocodile head scales. These results validate the conjecture that these scales emerge from a mechanical process^6^, but invalidate the more specific hypothesis that folding arises from tension-driven local epidermal proliferation caused by a heterogeneous tensile stress field^6^. Instead, our results support the alternative hypothesis that the natural patterning of crocodile head scales occurs due to a global compressive stress field caused by differential near-homogeneous growth, and/or differential material properties, of the dermis versus the epidermis.

## Post-embryonic effect of EGF treatment

**Extended Data Fig. 6:**
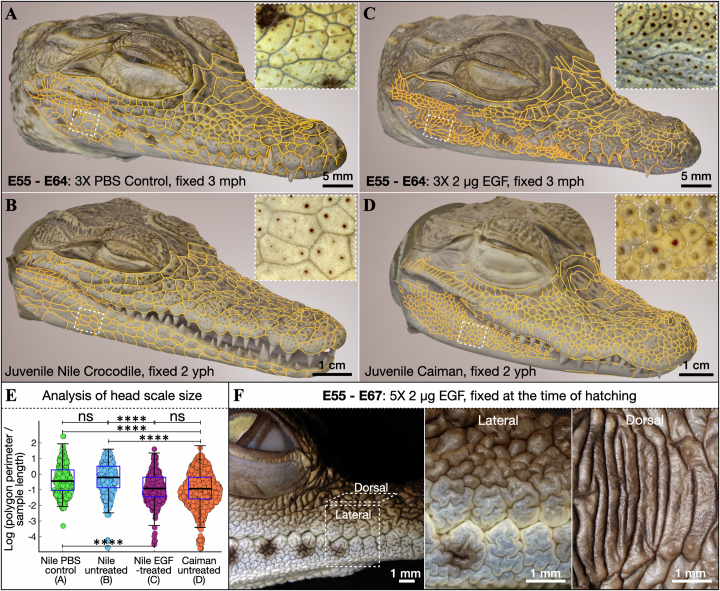
EGF-modified head scale patterning in hatched crocodiles. PBS control and EGF-treated crocodile embryos were incubated until the hatching phase to examine the post-embryonic pattern of head scales. We analyse the 3D scale fold networks of these samples and compare them with those of a juvenile Nile crocodile and a Caiman. **(A)** Control embryos injected with PBS and fixed at 3 months post hatching (mph) exhibit normal patterning of irregular polygonal head scale domains. **(B)** The patterning of head scales in a juvenile Nile crocodile (2 years post hatching, yph) is comparable to that of the 3 mph individual. **(C)** Parts of the convoluted ‘brainy’ pattern of embryos injected with 2 µg EGF between E55 and E64 subsequently relax towards a pattern of abnormally small polygonal scale domains (inset) on both the upper and lower jaws. **(D)** The relative sizes of polygonal scales in the EGF-modified head scale pattern shown in (C) are comparable to those of juvenile Caimans (here at 2 yph). **(E)** Using a one-way ANOVA test, we observe a statistically significant difference in polygonal scale relative size between the control and EGF-treated crocodile, whereas there is no significant difference in relative scale size between the EGF-treated crocodile and the Caiman (p = 0.889) (n = 1 biologically independent samples per group, medians [thick black lines], 25–75 percentiles [blue boxes], and ranges [black brackets] are shown). **(F)** An embryo with sustained EGF treatment (5X injections of 2 µg) from E55 to E67 reveals dramatic labyrinthine folding on both the upper and lower jaws, at the time of hatching. See Supplementary Table 1 for a summary of hatched crocodile experimental replicates. ns = non-significant, ****= p-value < 10^−4^.

We next sought to examine the effect of EGF treatments on the final pattern of head scales in hatched specimens. We therefore allowed a subset of EGF-treated embryos to develop until hatching (around E90) and beyond (Extended Data Fig. 6, Supplementary Table 2 and Supplementary Figs. 6–9). We then reconstructed the 3D surface geometry of these samples (Methods) and used the corresponding segmented fold networks to undertake a quantitative analysis of relative polygonal scale size among samples. Embryonic crocodiles treated with three PBS control injections from E55 to E64 exhibit normal patterning of non-overlapping polygonal head scales after hatching (Extended Data Fig. 6a). This pattern subsequently remains stable, and consists of scales that are statistically comparable in relative size to those of juvenile Nile crocodiles at 2 years after hatching (Extended Data Fig. 6b,e), indicating that the head-scale pattern in Nile crocodiles is fully established at around E75 (ref. ^6^).

**Extended Data Fig. 7:**
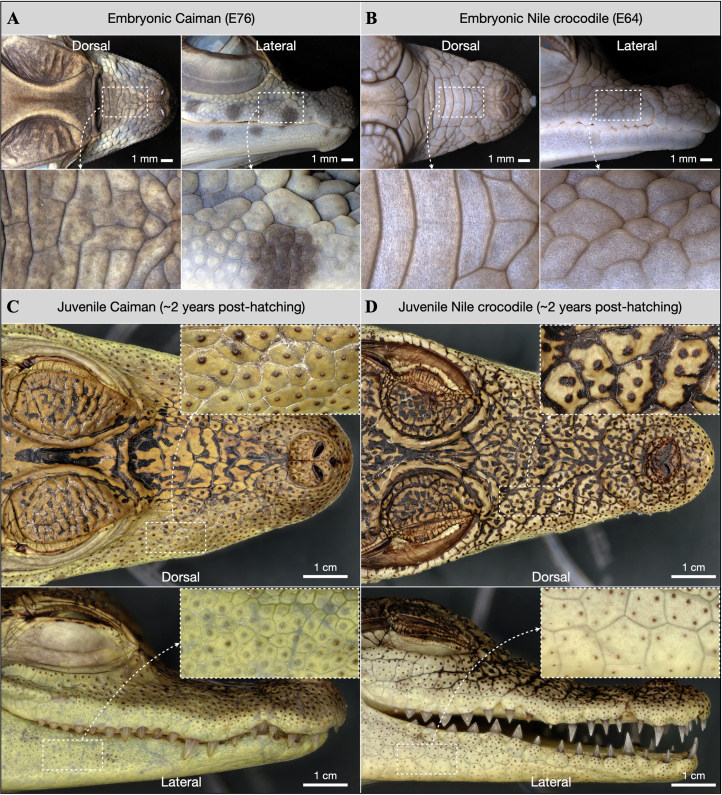
Nile crocodiles and caimans show distinct head scale patterns. **(A)** An embryonic caiman (E76) exhibits additional folds punctuating the longitudinal scale domains on the dorsal upper jaw surface and more numerous scale domains on both the latero-dorsal and lateral surfaces of upper and lower jaws. **(B)** This pattern is distinct from that of the embryonic Nile crocodile (E64), which exhibits larger polygonal scale domains. **(C-D)** Juvenile caiman (~2 years post-hatching) also exhibit smaller, more numerous polygonal head scale domains, in comparison to the Nile crocodile at the same age.

Conversely, when embryos treated with three injections of 2 μg EGF are allowed to hatch 4 weeks after the treatment has ended, they exhibit a skin folding pattern that is partially labyrinthine and partially made of polygonal head scales (Extended Data Fig. 6c (inset)). In other words, the brainy pattern that is generated by a strongly exacerbated EGF-induced epidermal growth and differentiation within a short period (9  days) of embryonic development (Fig. 2c) partially settles at later stages into a new steady-state pattern of significantly smaller polygons (Extended Data Fig. 6e). Notably, these small head scales are reminiscent of other crocodile species, such as those of the spectacled caiman (*Caiman crocodilus*), that are characterized by smaller and more numerous head scales, both in their embryonic and juvenile forms (Extended Data Figs. 6d and 7). Indeed, the polygonal scales of the hatched Nile crocodiles with arrested EGF treatment are statistically comparable in relative size to those of the juvenile caiman at 2 years after hatching (Extended Data Fig. 6c–e). Thus, variation of embryonic epidermal growth and/or differentiation provides a simple evolutionary mechanism explaining the differences in head-scale patterns among different crocodilian species.

We also investigated the effect of maintaining the EGF treatment over a longer period of embryonic development by injecting three crocodile embryos with five doses of 2 μg EGF (from E55 to E67) and collecting them at the hatching stage (around E90). A single embryo survived this intensive treatment, exhibiting a permanent transition from polygonal domains into extensive labyrinthine folding (Extended Data Fig. 6f and Supplementary Fig. 10).

## A biomechanical growth model

**Fig. 3:**
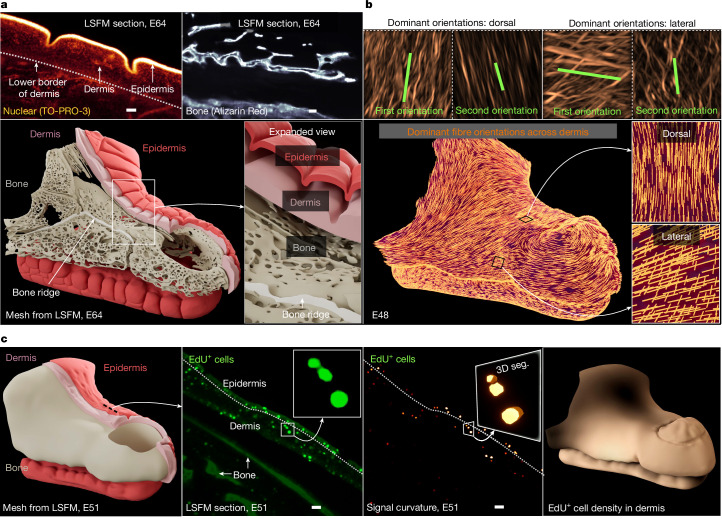
Estimating the mechanical parameters of crocodile head-scale patterning using LSFM. **a**, Nuclear staining (TO-PRO-3 or YO-PRO-1 iodide) enables segmentation of the epidermis and the dermis, and the bone tissue was segmented using Alizarin Red (Supplementary Video 6). **b**, Smoothed spatial variation of collagen fibre dominant orientations was identified by 3D fast Fourier transform of 3D light-sheet images after Fast Green staining^25^. **c**, Segmentation of proliferating (EdU^+^) cells in the epidermis and dermis of an E51 embryo was performed with a 3D signal principal curvatures approach^36^, and the spatial variation of proliferating cell densities across the face and jaws was assessed by spectral least-squares approximation. Skin growth is substantially smaller on the dorsal region of the upper jaws (dark regions in the right image). Replicates of LSFM samples with nuclear staining and EdU labelling are shown in Supplementary Table 5. The spatial variation of the 3D collagen network architecture was validated in *n* = 8 individual biological replicates using either LSFM or confocal microscopy. Scale bars, 100 μm (**a**) and 50 μm (**c**).

Next, we used LSFM to quantify the mechanically relevant elements that dominate the dynamics of crocodile skin folding (Methods). In brief, we used nuclear staining to capture the precise geometries of the epidermis and dermis, as well as Alizarin Red staining to capture the geometry of the underlying bone (Fig. 3a and Supplementary Video 6). Second, using Fast Green^25^ staining, we determined the dominant orientations of dermal collagen fibres across the jaws (Fig. 3b). Third, we detected EdU-labelled cells (Supplementary Video 3) to determine the spatial variation of both epidermal and dermal growth, finding a substantially lower growth rate of the skin on the dorsal region of the upper jaws (Fig. 3c). Notably, segmentation of EdU^+^ and EdU^−^ cell nuclei in skin samples that were imaged at higher resolution indicates that the growth rates of the dermis and epidermis are similar, with the former being even 20% higher than the latter (Supplementary Table 3).

**Extended Data Fig. 8:**
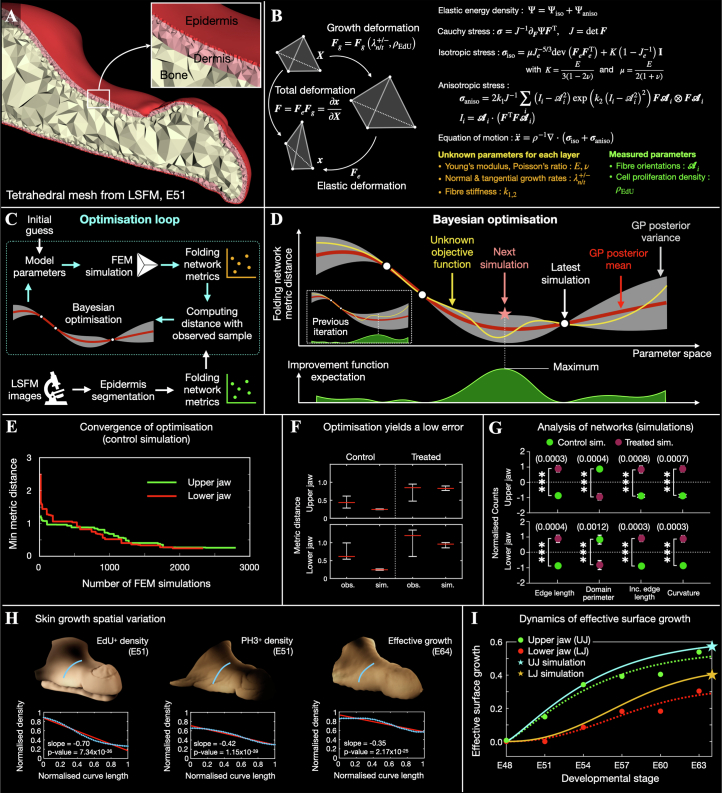
Mechanical growth model and Bayesian optimization of parameters. (**A**) Tetrahedral mesh generated for epidermis, dermis and bone layers of crocodile head embryo at stage E51. The geometry is segmented from LSFM imaging before smoothing of the epidermis layer. **(B)** The total deformation gradient, ${\boldsymbol{F}}$, of each tetrahedron is decomposed into growth, ${{\boldsymbol{F}}}_{g}$, and elastic, ${{\boldsymbol{F}}}_{e}$, components. ${{\boldsymbol{F}}}_{g}$ is determined from the relative epidermal and dermal cell proliferation densities, ${\rho }_{{\rm{EdU}}}$, with extreme values of normal and tangential growth values, ${G}_{N/T}^{+/-}$. The total Cauchy stress, $\sigma$, experienced by each tetrahedron is a function of the elastic energy density function, $\varPsi$, which itself is decomposed into isotropic (${\varPsi }_{{\rm{iso}}}$) and anisotropic (${\varPsi }_{{\rm{aniso}}}$) energy densities. For ${\varPsi }_{{\rm{iso}}}$, the neo-Hookean hyperelastic model is considered, whereas we adapted a collagen fibre model^35^ for ${\varPsi }_{{\rm{aniso}}}$. The isotropic and anisotropic stresses (${\sigma }_{{\rm{iso}}}$ and ${\sigma }_{{\rm{aniso}}}$) are computed from the deformation gradients and material properties of each layer. Among the model parameters, the fibre orientations (${{\mathcal{A}}}_{i}$) and the density of proliferating cells (${\rho }_{{\rm{EdU}}}$) are measured from LSFM images, while the Young’s modulus ($E$), Poisson’s ration ($\nu$), fibre stiffness, ${k}_{\mathrm{1,2}}$, and growth values ${G}_{N/T}^{+/-}$ are unknown. At each guessed set of values of the unknown parameters (see the optimization procedure in panel C), the steady-state geometry of the skin is computed by solving the equation of motion. **(C)** The unknown parameters of the mechanical model are estimated using a Bayesian optimization process. The latter starts from an initial guess followed by a FEM simulation run until steady state. The folding network metrics of the resulting geometry are then computed (see ‘Methods’) and are compared to the metrics of the observed folding network in a E64 embryo (extracted from LSFM images). The distance between the two metrics constitutes the optimality criterion to be minimized. Selection of the next set of parameters is performed through a Bayesian optimization algorithm. **(D)** A Gaussian process is used to determine the posterior probability function of the optimality criterion across the 6-dimensional parameter space. The associated posterior mean and variance functions allow computing an improvement function that is used to select the next sample. Both the posterior probability and improvement functions are updated after each observation, *i.e*., each simulation. The process is iterated until no improvement of the optimality criterion is achieved. **(E)** The Bayesian optimization process converges both for upper and lower jaws. **(F)** Both for upper and lower jaws, independent rounds of optimization (sim.) yield smaller or similar errors (metric distance values from the target real geometry) than between the same target and three other real samples at the same stage (obs.); n = 3 independent experiments, the red line shows the median value and the error bars shown the minimum and maximum values. **(G)** Curvature-based segmentation is used to quantify geometrical and topological features that distinguish three simulated controls (Control sim.) *versus* three simulated EGF-treated (Treated sim.) fold networks. *Inc. edge length* = length of incomplete edges, *i.e*., connected only at one end. Asterisks indicate significance levels: ***, <0.001; ****, <0.0001 (statistical significance is calculated using one-way ANOVA and individual p-values are shown in brackets; n = 3 independent experiments, error bars show standard deviation). (**H**) Skin growth is substantially reduced (dark colour on top panels) on the dorsal region of the upper jaw, as confirmed by multiple measurements: the densities of EdU-positive (EdU^+^) and PH3-positive (PH3^+^) cells at the onset of folding (E51 embryos), as well as the effective skin surface growth measured after near-completion of head scale patterning (*i.e*., in an E64 sample). Top panels: spectral least-square approximation of the 65 first modes describing the spatial variation of the corresponding measurement; bottom panels: variation of density (EdU^+^ cells, PH3^+^ cells, and effective skin surface growth) along the blue line indicated on the corresponding top panel; there is very strong statistical support for the three slopes to be negative (*i.e*., lower density values at the top of the jaws). Statistical significance is calculated using linear regression. (**I**) The dotted lines show the growth functions fitted on the surface effective growth data measured on the upper (green) and lower (red) jaws of Nile crocodile embryos at six developmental stages. The stars at E64 show the effective growth values measured on simulations whose final folding patterns were optimized through the Bayesian process for the upper (blue) and lower (orange) jaws. The plain lines are the growth dynamics (scaled from the corresponding dotted line to encompass the growth value measured on optimized simulations) used in the Supplementary Videos 7–11. Effective growth is measured, both in real samples and in simulations, by subtracting the integrated surface of the envelope of the jaws (generated by the alpha-shape function of Meshlab) from the integrated original folded surface.

We next used these data to build a 3D finite-element numerical growth model (Methods) to test the effects of both normal and EGF-enhanced epidermal growth and keratinization on crocodile head-scale patterning (Fig. 2b,c). In brief, the 3D volumes of the epidermis, dermis and bone are represented (both for upper and lower jaws) as tetrahedral meshes (Extended Data Fig. 8a). Growth-induced deformation is then realized through finite-strain theory applied to the neo-Hookean material model. The direction of fibres, as well as the spatial pattern of cell proliferation density (Fig. 3b,c), are fed to the mechanical model as known fields, whereas the elastic moduli, fibre stiffness and final amount of growth are considered to be unknown parameters and are determined through an optimization loop (Extended Data Fig. 8c). Importantly, the multidimensional Bayesian optimization process (Extended Data Fig. 8d–g) works by minimizing the difference between folding network metrics (Fig. 2e and Methods) in the simulated versus real target embryo. Thus, the optimization process is blind to which model parameter(s) should be changed, and to what extent, to achieve the target geometry. The target geometry is either that of a control embryo at E64 or an EGF-treated embryo at the same stage. Convergence efficiency of the optimization process is illustrated in Extended Data Fig. 8e,f.

## Simulating head-scale patterning

**Fig. 4:**
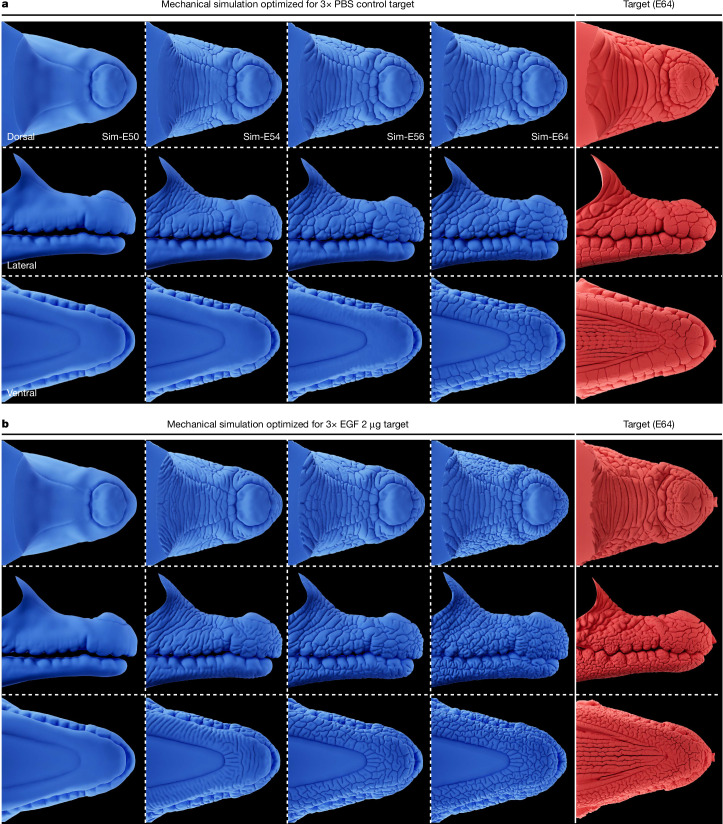
Mechanical growth simulations recapitulate natural and modified head-scale patterning. **a**, Mechanical modelling of skin growth accurately recapitulates natural head-scale patterning (Supplementary Video 7). Using growth dynamics derived from skin surface change measured from real samples (Extended Data Fig. 8i), surface folds propagate across the jaws and connect to form irregular polygonal domains, including elongated polygons on the dorsal jaw surface and smaller polygons on the lateral jaw surfaces. The numerical simulation steady state is comparable to the pattern observed in the E64 control target sample (right) used for Bayesian optimization. **b**, When the model parameters are optimized using the folding network metrics of an EGF-treated E64 target sample, the same mechanical model recapitulates its abnormal convoluted folding pattern with more numerous elongated polygons on the dorsal surface of the upper jaw and labyrinthine folding on the lateral and ventral surfaces (Supplementary Video 11).

Next, using the neo-Hookean mechanical model and Bayesian optimization described above, we simulated normal crocodile head-scale patterning (Fig. 4a) as well as the effect of EGF treatment on that mechanical developmental process (Fig. 4b). First, using an E64 control target for Bayesian optimization, we recapitulated the normal patterning of crocodile head scales (Fig. 4a): starting from a smooth geometry, the mechanical growth simulation produces surface folds, which propagate across the laterodorsal upper jaw. As the simulation progresses, these folds interconnect to form irregular polygonal domains, including large, elongated domains on the dorsal jaw surface, and smaller more regular polygonal units on the lateral sides of both jaws (Fig. 4a and Supplementary Video 7). These simulations integrate the spatial variation of growth derived from EdU labelling, as well as dynamics of skin surface change measured from real samples (Extended Data Fig. 8h,i).

**Extended Data Fig. 9:**
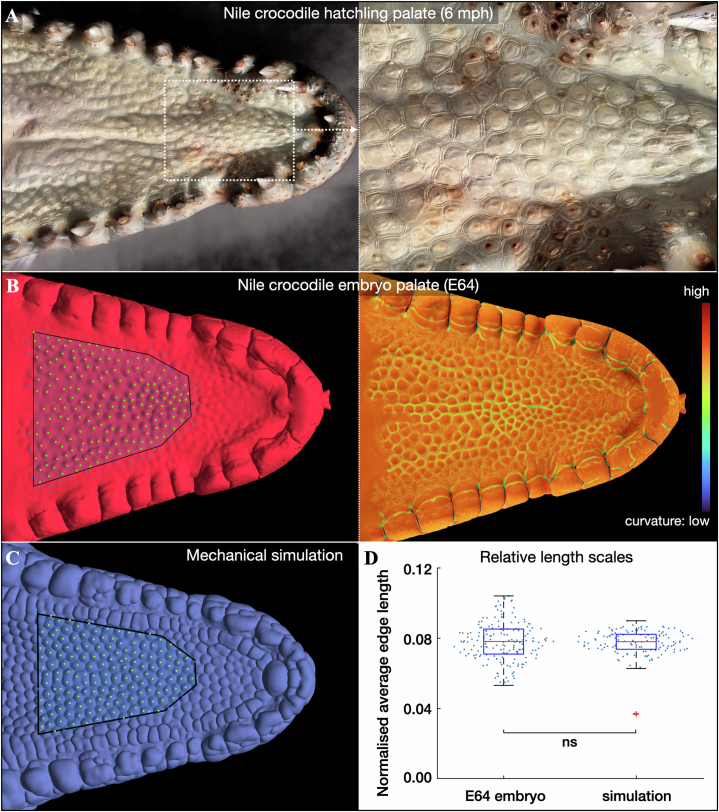
Patterning of the crocodile palate. (**A**) The palate (left, 2D image; right, magnified region) of a juvenile six-month old Nile crocodile is covered by nodular elements. **(B)** Surface reconstruction (left, TO-PRO-3 staining LSFM imaging; right, curvature field) of the palate of an E64 Nile crocodile embryo reveals these developing nodular elements. The Delaunay triangulation among well-defined elements is shown. **(C)** Our mechanical model recapitulates the emergence of these nodular structures. The Delaunay triangulation among elements in the same region as in (B) is shown. **(D)** Distributions of mean first-neighbour distance among nodular elements are not statistically different (one-way ANOVA test p-value = 0.38) between one observed (LSFM) and one simulated pattern; medians (red lines), 25–75 percentiles (blue boxes), and ranges (black brackets) are shown.

Notably, without modifying any parameters, our simulation also recapitulates the patterning (including its proper length scale) of the small, nodular elements located on the palate of the upper jaw (Extended Data Fig. 9). Note also that the inclusion of the bone ridges of the upper jaw (Fig. 3a) is required for the proper alignment of the lateral borders of the elongated dorsal scales, as indicated by numerical simulations omitting these bony features (Supplementary Video 8). Furthermore, failing to account for the observed smaller skin growth on the dorsal region of the upper jaws prevents the development of properly elongated scales (Supplementary Video 9). Our simulations also indicate that the compression-driven propagating folds avoid ISOs, and generate some incomplete edges, as well as a large number of 90° edge junctions at the domain boundaries (Supplementary Fig. 11). Overall, our growth simulations accurately recapitulate the proposed mechanically-driven process of head-scale patterning observed in Nile crocodiles.

As we propose that collagen network architecture has a substantial role in crocodile head-scale patterning, we incorporated the spatial variation of the dominant orientation(s) of dermal collagen fibres (Fig. 3b) in the simulations above (Fig. 4 and Supplementary Video 7). To test the importance of the anisotropic response of collagen fibres to homogeneous stress, we repeated our simulation while ignoring the collagen fibres (Supplementary Fig. 12 and Supplementary Video 10). These simulations do not produce elongated domains on the dorsal surface of the upper jaw, and other scale domains are also abnormally small. This result confirms that collagen architecture is an essential mechanical determinant of crocodile head-scale patterning.

Next, we optimize our mechanical growth simulations using an EGF-treated target sample (Fig. 4b (right)). As only relative stiffnesses are relevant to mechanical modelling (Methods), we constrain this Bayesian optimization process to only modify the relative values of epidermal parameters, whereas those of the dermis and bone layers remain unchanged. The optimized simulation exhibits extensive additional folding on both the upper and lower jaws (Fig. 4b and Supplementary Video 11). Indeed, as observed in EGF-treated samples (Fig. 2c), the simulated pattern includes thinner and more numerous elongated domains on the dorsal surface of the upper jaw as well as labyrinthine (brainy) folding on the lateral and ventral jaw surfaces.

Notably, although the optimization procedure is blind to which model parameter ratio(s) should be changed (and by how much) to achieve folding network metrics that match those of the EGF-treated embryonic samples, it resulted in increased values of three key epidermal parameters for both the lower and upper jaws: Young’s modulus, tangential growth and Poisson’s ratio (Supplementary Table 4). This is in agreement with our observations of an EGF-dose-dependent increase in both tissue stiffness (Fig. 2f) and epidermal growth (Supplementary Fig. 5), thereby highlighting the biological importance of these Bayesian-optimized parameters. Overall, our mechanical simulations successfully recapitulate the effect of EFG treatment on head-scale patterning.

## A mechanical morphospace of skin folding

**Fig. 5:**
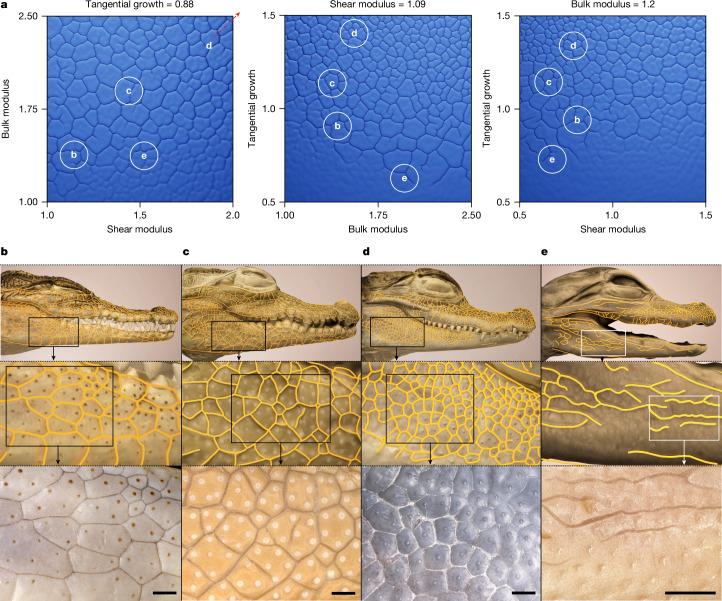
Morphospace of crocodilian head-scale patterns. **a**, Gradient plots showing that different folding patterns are obtained when smoothly varying three mechanical parameters (bulk modulus, shear modulus and tangential growth; bulk and shear moduli are related to Young’s modulus and Poisson’s ratio through Supplementary equation (4–7)). The white circles indicate examples of approximate parameter values generating head-scale patterns of the four species illustrated in **b**–**e**. **b**–**e**, Successively magnified regions of the scale edge networks in four species of crocodilians: the Nile crocodile (*C. niloticus*; 2 years post-hatching; **b**), the marsh crocodile (*Crocodylus palustris*; 2 years post-hatching; **c**), the spectacled caiman (*Caiman crocodilus*; 2 years post-hatching; **d**) and the American alligator (*Alligator mississippiensis*; 1 month post-hatching; **e**). The alligator lower jaw folds are particularly shallow; thus, their contrast in the bottom right image has been enhanced. Scale bars, 1 mm (**b**–**e**).

**Extended Data Fig. 10:**
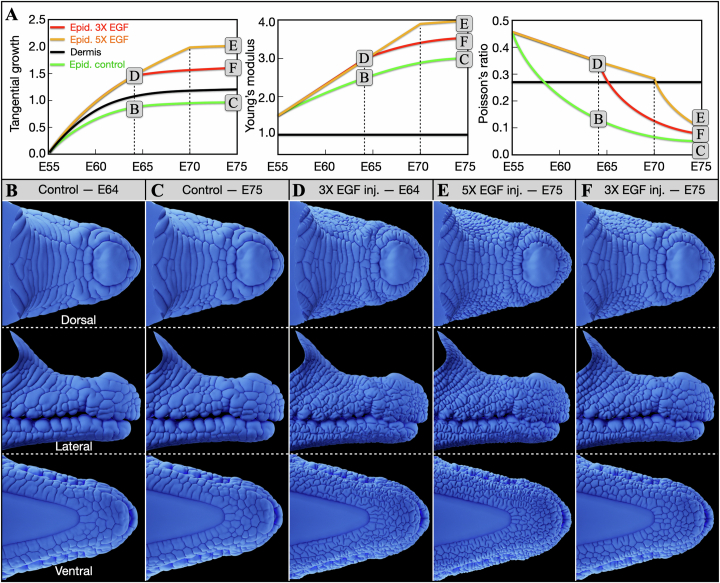
Simulating the transition from labyrinthine folding to caiman-like small polygons. (**A**) Simulating the increased growth (left panel) and increased effective stiffness (central panel) — while optimizing for the effective compressibility (right panel) — of the upper jaw epidermis under control and treated conditions recapitulates all the experimentally observed patterns. The parameter values obtained for the lower jaws are very similar (Supplementary Table 4). **(B, C)** Simulations under moderate epidermal growth and increasing epidermal stiffness, recapitulate the natural patterning of Nile crocodile head scales at E64 (B) and E75 (C) or hatching. **(D, E)** Exacerbated epidermal growth and differentiation due to three injections of 2 µg EGF at E55, E58 and E61 generates a brainy pattern of folds at E64 that becomes even more convoluted with sustained EGF treatment (5X injections of 2 µg) from E55 to E67. **(F)** When simulating arrested EGF treatment at E64, the brainy pattern partially relaxes into a caiman-like pattern of more numerous and smaller polygonal head scales at E75. All these dynamics are also illustrated in Supplementary Video 12.

Our simulations above indicate that, under Bayesian-optimized parameters of skin growth and material properties, our biomechanical model recapitulates (1) the normal polygonal pattern of Nile crocodile head scales (Fig. 4a, Extended Data Fig. 10a–c and Supplementary Video 7) and (2) the convoluted pattern of E64 embryos previously treated with three injections of EGF (Fig. 4b, Extended Data Fig. 10d and Supplementary Video 11). If we simulate continued exacerbated epidermal growth and differentiation beyond E64, we recapitulate at E75 (Extended Data Fig. 10e) the convoluted labyrinthine folding pattern observed after a sustained EGF treatment between E55 and E70 (Extended Data Fig. 6f and Supplementary Fig. 10). Notably, when we simulated the arrest of EGF treatment after E64 (that is, switching back to the normal growth rate), we observed that the simulated convoluted pattern partially transforms to a new pattern at E75 that includes skin areas with small polygons (Extended Data Fig. 10f and Supplementary Video 12). This new steady state mimics the caiman-like pattern that we observed in hatched and juvenile Nile crocodiles that were treated with EGF before E64 (Extended Data Fig. 6c–e). Thus, variations in embryonic growth and material properties of skin layers provide a simple evolutionary mechanism producing a diversity of head-scale patterns among crocodilian species. To further investigate this conjecture, we produce a theoretical morphospace of skin folding patterns (Fig. 5a) by varying the relative epidermal versus dermal growth rates and elastic moduli. This analysis shows that such variations enable us to produce the whole diversity of observed crocodilian head-scale patterns, including those of Nile crocodiles, marsh crocodiles, spectacled caimans and American alligators (Fig. 5b–e).

## Conclusions and discussion

It is increasingly clear that biological shape generation involves key mechanical processes^33^ effective at the mesoscopic scale^17,34^. We previously demonstrated^6^ that the irregular polygonal head scales of crocodiles are not placode-derived developmental units^1,8,10^ that emerge through Turing-like chemical patterning^2,3,16^. Instead, they arise from a mechanical process producing geometrical features and dynamics superficially reminiscent of material cracking in a tensile stress field^6^. Here we show that the patterning of crocodile head scales in fact emerges from compressive mechanical instabilities. Note that these specific elastic instabilities are not caused by differential growth of the dermis versus the epidermis, but instead by their differential material properties (primarily, stiffness) and their greater growth relative to the underlying tissues. Overall, we demonstrate that evolution has produced two ways of generating crocodilian scales: chemical Turing instabilities (body scales) and mechanical instabilities (head scales).

More specifically, we invalidate the tension-driven local-growth hypothesis of crocodile head-scale development^6^ and validate the conjecture of compression-driven mechanical patterning by producing crocodile embryos with substantially amplified folding patterns after treatment with EGF, a protein that increases epidermal growth and differentiation (Fig. 2c, Extended Data Fig. 6f and Supplementary Video 4). Notably, if the EGF treatment is arrested early enough in development, regions of this brainy pattern of skin folds relax towards a caiman-like pattern of polygonal scales that are substantially smaller than those of untreated Nile crocodiles (Extended Data Fig. 6). Consequently, we suggest that the diversity of head-scale patterns among crocodilian species results from evolutionary changes in simple mechanical parameters, such as differential growth and material properties of the dermis versus the epidermis (Fig. 5).

Our phenomenological mechanical growth model, derived from LSFM imaging, validates the compression-driven hypothesis of crocodile head-scale patterning. It incorporates the 3D geometries of the epidermis, dermis and skull, as well as the spatial variation of skin growth and of dermal collagen fibre orientations^25^ (Fig. 3 and Supplementary Video 13). Importantly, for the simulated folding network metrics to match those of the EGF-treated crocodile embryos (Fig. 4b and Supplementary Video 11), the blind optimization process generates relative values of growth, stiffness and incompressibility of the epidermis that are larger in comparison to those optimized for the normal head-scale pattern of control crocodiles. This is important in two respects. First, EGF is known to increase epidermal (but not dermal) growth and keratinization, resulting in increased epidermal effective stiffness. Second, our nanoindentation measurements confirm that the epidermal surface of the skin is stiffer in EGF-treated than in control samples. Note also that, for the simulated folding network metrics to match those of control embryos (Fig. 4a and Supplementary Video 7), the optimization process correctly yields a stiffer epidermis than dermis. Thus, parameter optimization in our phenomenological model yields epidermis versus dermis relative parameter values that are biologically relevant and conform to our experimental manipulation of crocodile development.

Finally, our study highlights the power of phenomenological models for investigating biological patterning processes. Reaction–diffusion modelling has demonstrated that skin colour patterns^2,3^, as well as the patterning of placode-derived skin appendages^3,5,15^, can be efficiently and quantitatively investigated mathematically at the mesoscale with phenomenological models, that is, while ignoring most of the unmanageable profusion of underlying cellular and molecular variables^3,35^. Similarly, the analyses presented here show that simple elastic models can efficiently recapitulate certain mechanical morphogenetic processes, such as the development of a variety of head-skin folding patterns in multiple crocodilian species (Fig. 5), while ignoring some additional properties of biological tissues, such as the plastic response to stress.

## Methods

### Animal husbandry

Fertilized crocodile eggs (imported from Seronera Crocodile Farm) were transported to the University of Geneva and incubated at 31 °C in moist vermiculite. All treated and non-treated crocodile embryos were fixed and stored in 10% neutral buffered formalin (NBF). All non-fluorescence imaging of embryonic crocodile samples was undertaken using the Keyence VHX 7000 digital microscope. Imaging of hatched crocodile specimens was undertaken using an overhead-mounted Nikon D800 camera. Maintenance of, and experiments with, crocodile embryos and juveniles were approved by the Geneva Canton ethical regulation authority (authorization GE10619B) and performed according to Swiss law. The sample sizes are specified in figure legends and the Supplementary Information. Randomization and blinding was not required.

### Nanoindentation

A Piuma nanoindenter (Optics11) was used to acquire stiffness measurements (effective Young’s modulus, Pa) of embryonic crocodile skin surface. When comparing measurements in two skin samples, a change in epidermal keratinization will produce a change in surface stiffness, which is very likely to be correlated with a change of the same sign in the effective overall Young’s modulus of the whole multilayered epidermis. In other words, an increase in epidermal surface stiffness is very likely accompanied by an increased stiffness of the whole epidermis. Freshly dissected upper jaws were positioned lateral side upwards, submerged in PBS. A probe with a tip radius of 99 µm and stiffness rating of 0.48 N m^−1^ was used to indent at a depth of 2,000 nm. Only measurements from load-displacement curves with a Hertzian contact model fit of ≥95% were subsequently analysed. Each biological replicate for the embryonic nanoindentation series was indented 10 times (Fig. 1c) or 5 times (Fig. 2f). These indentation points were positioned in a single row with each point separated by 120 µm. Plots showing the mean effective Young’s modulus values for each biological replicate with s.d. are presented. Statistical analysis was undertaken in Prism 9 (GraphPad).

### Confocal microscopy

Confocal microscopy was used for embryonic crocodile samples stained with the Fast Green FCF dye (Sigma-Aldrich) according to a protocol of whole-mount collagen staining^25^. Image acquisition was undertaken as previously described^25^, using the SP8 microscope (Leica Microsystems), with a ×63 oil-immersion objective (numerical aperture, 1.4). Fast Green was excited at 627 nm and the signal was detected in the range of 630–730 nm. Image reconstruction was undertaken using Imaris (Oxford Instruments).

### H&E staining

Fixed embryonic crocodile samples were dissected and embedded in paraffin as previously described^24^. Paraffin sections were cut at 10 µm with a RM2255 microtome (Leica Microsystems) before staining with haematoxylin and eosin (H&E). Slides were imaged using an automated slide scanner (3DHISTECH).

### In ovo intravenous EGF injections in crocodiles

The injection of crocodile eggs was undertaken in accordance with our previously published work^20,21^ (https://youtu.be/qCYWSgbffnY). Crocodile eggs were incubated until the appropriate developmental stage and then cleaned with 70% ethanol. Eggs were candled to identify a suitable vein for injection, and a detailing saw (Micromot 50/E, Proxxon) was used to remove the shell while keeping the underlying membrane intact. The eggshell was then removed using fine forceps, and mineral oil was applied to the membrane with a cotton bud, thereby increasing membrane transparency to allow clear visualization of the underlying veins. The samples were injected with either 30 µl of PBS as a control or 30 µl of PBS containing recombinant murine EGF (PeproTech). Different doses of EGF were injected (0.625 µg, 1.5 µg or 2 µg). Patent Blue was also added to the solution to enable visualization of the solution entering the vein during injection. Injections were undertaken using a Hamilton syringe attached to a micromanipulator (MM33 right, Marzhauser). Once injected, the eggs were cleaned to remove excess mineral oil, and the eggshell window was covered with adhesive tape. Treated embryos were then returned to their incubator. The samples were each injected three times over the course of 10 days for each experiment (Fig. 2a). At collection, the embryos were treated with an intravenous injection of EdU to label proliferating cells (Baseclick); embryo collection and fixation were undertaken 3 h after EdU injection. Some EGF-treated embryos were used for nanoindentation at the end of the experiment, and some others were incubated until hatching. Embryos were subsequently fixed in 10% NBF at 4 °C and imaged with a Keyence VHX 7000 digital microscope. Every embryo injected with EGF exhibited modified head-scale patterning. All of the replicates from these experiments are shown in Supplementary Fig. 4 and are summarized in Supplementary Table 1.

The drug that we use here (EGF) has the remarkable property of specifically promoting epidermal growth and differentiation without exhibiting strong deleterious effects in other aspects of in vivo embryonic development. Further validation of the parameters involved in the compression-folding process of crocodile head-scale patterning will require the identification of other drugs that would specifically affect one parameter at the time. For example, it would be particularly interesting to pharmacologically perturb the 3D architecture of collagen in developing crocodile embryos to investigate the corresponding effects on skin folding of the dorsal versus lateral upper jaw surface. Unfortunately, drugs currently known to effect collagen organization (such as β-aminoproprionitrile, BAPN) are highly toxic in vivo as they strongly affect the development of multiple connective tissues such as skin, bones and blood vessels. Given the great difficulties of experimentation with crocodile embryos, the screening of drugs that could, in vivo, specifically affect one mechanical parameter at a time in the skin, could be initially performed in a more classical model (such as the chicken) with more reliable source of embryos.

### LSFM

The upper and lower jaws of fixed embryonic crocodile samples were dissected, dehydrated into methanol, and bleached with hydrogen peroxide, before rehydration and permeabilization in PBS with Triton X-100 (Sigma-Aldrich) (PBST). For nuclear staining, the samples were incubated in either TO-PRO-3 iodide or YO-PRO-1 iodide (3:1,000, Thermo Fisher Scientific) for 6 h. EdU-positive cells (EdU^+^) were detected using the EdU detection kit manufacturer’s guidelines (Baseclick). The samples were then dehydrated into methanol and collagen staining was undertaken in anhydrous conditions with the same Fast Green protocol^25^ as for confocal microscopy (see above). Samples were then cleared according to the iDISCO+ protocol^37^. Upper and lower jaw samples were imaged separately using a light-sheet microscope (Ultramicroscope Blaze, Miltenyi Biotec). Selected specimens were restained with Alizarin Red in potassium hydroxide (KOH) and re-imaged to visualize the developing calcified bone matrix (Extended Data Fig. 1b). Image stacks were processed using ImageJ^38^, before rendering with the Redshift engine of Houdini (SideFX) and the Unreal Engine (Epic Games). A summary of replicates used for LSFM is shown in Supplementary Table 5. Each sample includes both upper and lower jaws, which we scanned separately.

### 3D reconstructions of hatched crocodiles

Using our custom-built imaging system^39^, combining a robotic arm, high-resolution camera and illumination basket of light-emitting diodes, we combine ‘photometric stereo’ and ‘structure from motion’ to reconstruct the precise 3D surface mesh and colour-texture of hatched crocodile heads (Fig. 5b–e and Extended Data Fig. 6a–d). To compare the polygonal scale sizes among individuals, we first compute the minimum principle curvature of the meshes. Then, the folding network of each sample is computed by applying a skeletonization algorithm^40^, followed by graph simplification (using MATLAB R2021a), on the negative curvature regions of the mesh. Using the colour texture of meshes, the folding networks were manually completed and cleaned using Houdini (SideFX).

### Segmentation of LSFM data

Using TO-PRO-3, YO-PRO-1, EdU, Alizarin Red and Fast Green staining (see above), we segmented the light-sheet microscopy data to extract (in both the upper and lower jaws) the geometry of the epidermis, dermis and bone tissues (Supplementary Video 6), as well as the dominant orientations of the dermal collagen fibres, and the distribution of proliferating cells in the dermis and epidermis. The segmented data were used to build a finite element model (FEM, see below) of the crocodile head.

Cell nuclei staining signal enables precise segmentation of the epidermis from the dermis because the former exhibits a higher cell density (Fig. 3a). More specifically, the 3D image generated by LSFM on the basis of the TO-PRO-3/YO-PRO-1 fluorescence signal was subjected to 3D Canny’s edge detection^41^ in MATLAB-R2021a, generating a 3D binary image in which non-zero voxels form point clouds corresponding to two 3D surfaces: the surface of the epidermis and the epidermis–dermis boundary. For each of these two surfaces, we compute at each point the surface normal vector from the intensity gradient. The position of points and their corresponding normal vectors are then fed to a screened Poisson surface reconstruction algorithm^42^ in Meshlab^43^ to reconstruct triangular surface meshes, which effectively represent the initial point clouds in a much lighter format: 3D meshes are much easier to manipulate, for example, with the Laplacian smoothing algorithm to filter out the artifactual stair-step patterns in the original voxelized data format. The epidermis surface and the epidermis–dermis boundaries allow for computing the epidermis thickness across each control and treated sample at different developmental stages.

Collagen network 3D architecture is likely to become instrumental in biomechanical modelling^25,26^ because it endows tissues with distinctive mechanical properties such as anisotropic response to homogeneous stress. Thus, we assess the orientation(s) of collagen fibres in the dermis across the face and jaws of developing crocodile embryos (Fig. 3b). To this end, we use our recently published whole-mount Fast Green staining method, which provides unmatched visualization of 3D collagen network architecture via confocal or light-sheet microscopy^25^. In brief, (1) the two most dominant orientation(s) of populations of collagen fibres were identified by determining the dominant 3D Fast Fourier transform coefficients in each of 13,000 homogeneously distributed dermal samples (cubic patches of 50 × 50 × 50 voxels) of 3D light-sheet images (Supplementary Note 1); (2) smoothing of the spatial variation of fibres orientations was achieved with an exact optimization procedure using a fibre axis mismatch energy functional (Supplementary Note 2); and (3) the two dominant fibre orientations, both tangential to the dermis mid-plane, were interpolated using spectral least-squares approximation (Supplementary Note 3).

After standard EdU labelling and detection (Supplementary Video 3), we used a 3D principal curvatures approach^36^ (on the fluorescence signal) to segment proliferating cells in the jaws of an embryonic crocodile at E51, that is, at the onset of head-scale emergence (Fig. 3c). This approach is highly efficient for individually segmenting cells when they are grouped (that is, in contact). As the signal intensity is embedded in a 3D domain, three signal principal curvatures *k*_1,2,3_ are computed (in MATLAB) for each voxel, and voxels characterized by *k*_s_ > *k*_threshold_, where ${k}_{s}={({k}_{1}^{+}{k}_{2}^{+}{k}_{3}^{+})}^{\frac{1}{3}}$ and ${k}_{i}^{+}=\max ({k}_{i},0)$ are stored. The centroid of the connected voxels is considered as the location of an EdU^+^ cell. We then compute the density of EdU^+^ cells, separately for the dermis and the epidermis, by choosing sampling points in the corresponding segmented tissue layers. The space surrounding each sampling point is limited to a box of 80 × 80 × 80 voxels clipped by the layer boundaries. The density of EdU^+^ cells at a sampling point is computed as the number of cells inside the clipped box divided by its volume. In our numerical model, densities of proliferating cells are represented as a space-dependent growth function. We transfer this information to the 3D model using a spectral least-squares approximation approach to interpolate data on the spatial modes of the target mesh (details are provided in Supplementary Note 3).

For segmenting bone tissue, we use either the 3D Canny’s edge detection of the (very strong) Alizarin Red signal or a semi-automatic procedure for samples with (weaker) Fast Green or EdU signals. In the latter case, we (1) choose several sections in the *x*, *y* and *z* directions and manually mark the separation between the dermis and the bone, (2) store the coordinates of all profile points as a 3D point cloud and compute their normal with Variational Implicit Point Set Surface^44^ and (3) use screened Poisson surface reconstruction^42^ from Meshlab^43^ to generate the mesh corresponding to the bone surface.

### A biomechanical model of head-scale emergence

We use the segmented data to build a 3D finite-element numerical growth model. Triangular meshes were generated, both for upper and lower jaws, at the surface boundaries of the epidermis, dermis and bone of embryos before the onset of head-scale patterning (Fig. 1b and see above). The epidermis surface and the epidermis–dermis interface were smoothed to remove any artificial local deformations associated with sample preparation, including dehydration into methanol. The 3D volume of each of the three layers was represented as a tetrahedral mesh generated with TetGen^45^ (Extended Data Fig. 8a).

During simulated growth, the deformation of tetrahedral elements is realized through finite-strain theory in which the bulk material configuration at current time *t* is represented as the spatial coordinates of a collection of points in the form of a vector variable **x** = **x**(**X**,*t*), where **X** is the spatial coordinates of these points at a reference configuration, that is, at *t* = 0 (Extended Data Fig. 8b). The coordinates between the current and the reference configurations are connected by the deformation gradient map, ***F***—that is, a second-order tensor that incorporates the elastic and growth deformations. The elastic energy and the mechanical stress stored in each tetrahedral element is then calculated from the neo-Hookean material model, known to behave appropriately under large deformations^30,31,46^, and allowing the incorporation of anisotropic material, such as collagen fibres^47^ (Supplementary Note 4). The direction of fibres, as well as the spatial pattern of cell proliferation density, both inferred from LSFM data (Fig. 3b,c), are fed to the mechanical model. However, the elastic moduli, fibre stiffness and final amount of growth are considered as unknown parameters. Note that the absolute values of stiffness are irrelevant in the numerical simulations as the model key parameters are the fibre stiffness relative to the dermis and epidermis moduli, as well as the ratio of epidermis to dermis stiffnesses (Young’s moduli).

### Numerical simulations and parameter optimization

To perform numerical simulations, the mechanical model formulation described above is discretized for tetrahedral elements using the FEM and integrated with contact and viscous forces (Supplementary Note 5). The final model is then implemented in an in-house application that uses NVIDIA GPUs for high-performance computation. For that purpose, we used the CUDA programming language to develop intensive-computation kernels, whereas C++ is used for data management, geometry processing, input/output operations and the graphical user interface. Our application integrates the following open-source libraries: Dear ImGui (https://github.com/ocornut/imgui, MIT licence) for the graphical user interface, CUDA C++ Core Libraries (https://github.com/NVIDIA/cccl, Apache-2.0, FreeBSD, BSD-3-Clause licences) for parallel algorithms, Eigen (https://gitlab.com/libeigen/eigen, MPL-2.0, BSD licences) for linear algebra and libigl (https://github.com/libigl/libigl, GPL-3.0, MPL-2.0 licences) for geometry processing. The simulation input is a tetrahedral mesh that defines the geometry of the crocodile head (epidermis, dermis and bone layers). Moreover, a set of model parameters are used: in addition to the dermal collagen fibres orientation and stiffness, we include, both for epidermis and dermis, the Young’s modulus and Poisson’s ratio, the growth rate functions and the cell proliferation pattern. The deformation of the skin is then computed and the final geometry is generated as a tetrahedral mesh.

The mechanical model is integrated with a Bayesian optimization process (bayesopt library from MATLAB R2021a with parallel sampling), that is, a machine-learning global minimization algorithm. The optimality criterion consists of the distance between the metrics (integrating multiple topological and geometrical features, see below) of the steady-state simulated geometry versus LSFM-acquired meshes. To compute the metrics of a folding network, we first compute the minimum principle curvature of the corresponding surface mesh representing the epidermis boundary. We then segment the skin folds by applying a skeletonization algorithm^40^, followed by graph simplification (using MATLAB R2021a), on the negative curvature regions of the mesh. Next, we compute the following geometrical and topological features of the network: number of domains (cycles), perimeters of domains, lengths of edges, curvatures of edges and lengths of incomplete edges. The final metrics is a vector of which the components are the means of these features, normalized to the diagonal length of its bounding box. Given that components within a metrics vector may differ significantly among each other, we need to normalize them properly. For this purpose, we use LSFM data to compute the metrics of controls at E64 and treated individuals (2 μg EGF) at E64. We then compute the interindividual (that is, among all individuals) mean and s.d. of each component (Fig. 2e). We finally normalize the components of any newly computed metrics by subtracting the interindividual mean and dividing by the interindividual s.d.

Finding optimal parameter values for control and treated targets is performed in two steps. First, we use an E64 control target mesh and perform optimization on the six-dimensional parameter space, including epidermis Young’s modulus, *E*_epidermis_ (keeping *E*_dermis_ = 1); epidermis and dermis Poisson’s ratios, *v*_epidermis/dermis_; dermis tangential growth values, ${G}_{T,{\rm{dermis}}}^{+/-}$ (keeping ${G}_{T,{\rm{epidermis}}}^{+/-}$ at 80% of the dermis values); and the fibre stiffness, *k*_1_ (*k*_2_ being set to 0). Second, using a 2 μg EGF-treated target, we perform another optimization on the three-dimensional parameter space including epidermis-related parameters, that is, *E*_epidermis_, *v*_epidermis_ and ${\lambda }_{T,{\rm{epidermis}}}^{{\rm{EGF}}}$ (additional epidermal tangential growth induced by EGF). See Supplementary Notes 4 and 6 for the definitions of parameters and Supplementary Table 4 for the complete list of parameter values. To minimize the distance between the metrics vectors of the simulated versus LSFM target geometry (control or treated), we use a Gaussian process (that is, a generalization of the multivariate normal distribution to infinite dimensions) in the optimization loop to approximate posterior mean and variance functions from which the objective function is sampled (Extended Data Fig. 8d). The posterior functions are updated at each iteration according to Bayesian inference and this information is then used to compute the expectation of the improvement function, which measures the chance of observing an objective (that is, the distance between simulation and observation) smaller than the minimum objective observed so far (Supplementary Note 7). The optimization process, which typically takes a few thousand iterations, continues until no more improvement is observed in the last 500 iterations.

### Reporting summary

Further information on research design is available in the Nature Portfolio Reporting Summary linked to this article.

## Online content

Any methods, additional references, Nature Portfolio reporting summaries, source data, extended data, supplementary information, acknowledgements, peer review information; details of author contributions and competing interests; and statements of data and code availability are available at 10.1038/s41586-024-08268-1.

## Supplementary information

Supplementary Information Supplementary Tables 1–5, Supplementary Figs. 1–12 and Supplementary Notes 1–7.

Reporting Summary

Supplementary Video 1 Growth series (TO-PRO-3).

Supplementary Video 2 Growth series (Alizarin Red).

Supplementary Video 3 EdU labelling.

Supplementary Video 4 2 μg EGF versus control.

Supplementary Video 5 EGF dose comparison.

Supplementary Video 6 Tissue layer geometry.

Supplementary Video 7 Simulation of normal head-scale patterning.

Supplementary Video 8 Simulation without bony ridges.

Supplementary Video 9 Simulation with fully homogeneous growth.

Supplementary Video 10 Simulation without collagen anisotropy.

Supplementary Video 11 Simulation of EGF-induced head-scale patterning.

Supplementary Video 12 Simulations of the transition to caiman-like head scales.

Supplementary Video 13 3D model of the embryonic crocodile head at E64.

## Data Availability

All data are provided in the Article and its Supplementary Information.

## Code availability

The executables and original code used for the numerical simulations presented here are provided as a Code Ocean capsule (https://codeocean.com/capsule/7106541/tree/v2).
