# Supplementary material for: Self-organized patterning of crocodile head scales by compressive folding
Source: Nature. 2024 Dec 11;637(8045):375–83. doi: 10.1038/s41586-024-08268-1 (PMC11711089; doi:10.1038/s41586-024-08268-1)
Supplement: Supplementary file 1 — Supplementary Tables 1–5, Supplementary Figs. 1–12 and Supplementary Notes 1–7. [file 41586_2024_8268_MOESM1_ESM.pdf]

nature portfolio

https://doi.org/10.1038/s41586-024-08268-1

Supplementary information

Self-organized patterning of crocodile head scales by compressive folding

In the format provided by the authors and unedited

***Nature* 2024**

Self-organised Patterning of Crocodile Head Scales by Compressive Folding

Supplementary Information

Gabriel N. Santos Durán[1,†], Rory L. Cooper[1,†], Ebrahim Jahanbakhsh[1,†], Grigorii Timin[1] & Michel. C. Milinkovitch[1,2,*]

1. Laboratory of Artificial & Natural Evolution (LANE), Dept. of Genetics & Evolution, University of Geneva, 1211 Geneva, Switzerland.
2. SIB Swiss Institute of Bioinformatics, Geneva, Switzerland.

[†]These authors contributed equally to this work.

[*]To whom correspondence should be addressed:

Michel C. Milinkovitch,

Laboratory of Artificial & Natural Evolution (LANE),

Dept. of Genetics & Evolution, University of Geneva,

Sciences III, 30, Quai Ernest-Ansermet, 1211 Geneva 4, Switzerland.

Tel: +41(0)22 379 67 85; e-mail: Michel.Milinkovitch@unige.ch

ORCID ID: 0000-0002-2553-0724

Supplementary Information Guide

1. Supplementary Data

**Supplementary Table 1**	Summary of embryonic replicates from intravenous EGF treatments.
**Supplementary Table 2**	Summary of hatched EGF-treated crocodile samples.
**Supplementary Table 3**	Cell density measurements.
**Supplementary Table 4**	Values of the mathematical model effective parameters used in simulations.
**Supplementary Table 5**	Summary of embryonic samples used for LSFM.
**Supplementary Figure 1**	LSFM imaging of integumentary multi-sensory organs (ISOs) and collagen fibres in crocodile head scales at E63.
**Supplementary Figure 2**	Confocal imaging of collagen architecture prior to head scale emergence at E48.
**Supplementary Figure 3**	Proliferation is not localised at the tips of propagating scale folds.
**Supplementary Figure 4**	All replicates from intravenous EGF treatments during embryonic head scale patterning.
**Supplementary Figure 5**	The effect of EGF treatment upon head scale patterning is dose-dependent — histological sections
**Supplementary Figure 6**	Post-embryonic effect (at 1 mph) of *in-ovo* EGF treatment.
**Supplementary Figure 7**	Post-embryonic effect (at 3 mph) of *in-ovo* EGF treatment.
**Supplementary Figure 8**	Post-embryonic effect (6 mph) of *in-ovo* EGF treatment.
**Supplementary Figure 9**	Post-embryonic effect (9 mph) of *in-ovo* EGF treatment.
**Supplementary Figure 10**	Sustained *in-ovo* EGF treatment generates a labyrinthine skin surface folding pattern in hatched Nile crocodiles.
**Supplementary Figure 11**	Effect of integumentary sensory organs (ISOs) on head-scale patterning.
**Supplementary Figure 12**	Mechanical growth simulation without collagen anisotropy.

2. Supplementary Notes

**Supplementary Note 1**	Finding the dominant orientations of collagen fibres using 3D Fourier coefficients
**Supplementary Note 2**	Minimisation of fibre energy functional
**Supplementary Note 3**	Spectral least-square approximation
**Supplementary Note 4**	Finite-strain theory and anisotropic neo-Hookean material model
**Supplementary Note 5**	Computing forces in the FEM framework
**Supplementary Note 6**	Growth model
**Supplementary Note 7**	Bayesian optimisation

3. Supplementary Videos

**Supplementary Video 1**	Growth Series (TO-PRO-3)
**Supplementary Video 2**	Growth Series (Alizarin red)
**Supplementary Video 3**	EdU labelling
**Supplementary Video 4**	2 ug EGF vs Control
**Supplementary Video 5**	EGF dose comparison
**Supplementary Video 6**	Tissue layer geometry
**Supplementary Video 7**	Simulation of normal head scale patterning
**Supplementary Video 8**	Simulation without bony ridges
**Supplementary Video 9**	Simulation with fully homogeneous growth
**Supplementary Video 10**	Simulation without collagen anisotropy
**Supplementary Video 11**	Simulation of EGF-induced head scale patterning
**Supplementary Video 12**	Simulations of the transition to caiman-like head scales
**Supplementary Video 13**	3D model of the embryonic crocodile head at E64

1. Supplementary Data

Supplementary Table 1. Summary of embryonic replicates from intravenous EGF treatments

**Box/ Clutch Number**	Treatment time (Embryonic day)	**Sample numbers for treatment types and doses**					
		Fixed at start of treatment	PBS control	EGF 0.625 µg	EGF 1.25 µg	EGF 2 µg	Total
**1**	E48 - E57	4	10	10	13	-	37
**2**	E55 - E64	4	8	10	11	12	45
**3**	E51 - E60	4	8	9	9	10	40
**4**	E55 - E64	4	11	6	5	5	31

Supplementary Table 2. Summary of hatched EGF-treated crocodile samples. This table lists the specific crocodile samples that were incubated until hatching and into the post-hatching phase, after *in-ovo* treatment with EGF.

Clutch	Treatment length	Treatment type	Survival post hatching	Imaged (mph)
**2**	E55 - E64	3X PBS control	1/1	1, 3
**2**	E55 - E64	3X 2 µg EGF	1/2	1, 3
**2**	E55 - E64	3X 1.25 µg EGF	1/1	1, 3
**2**	E55 - E64	3X 0.625 µg EGF	1/1	1, 3
**3**	E51 - E60	3X PBS control	1/1	1, 3, 6, 9
**3**	E51 - E60	3X 2 µg EGF	3/4	1, 3, 6, 9
**3**	E51 - E60	3X 1.25 µg EGF	1/1	1, 3, 6, 9
**3**	E51 - E60	3X 0.625 µg EGF	1/1	1, 3, 6, 9
**4**	E55 - E67	5X 2 ug EGF	1/3	0
		**Total survival: 11/15**		

Supplementary Table 3. Cell density measurements. Total cell density was measured by TO-PRO-3 staining, and the proportion of these cells that are proliferating were identified by EdU detection. These values are measured separately for the dermis and the epidermis in a region of interest for an untreated (E51) and a control (E60) sample using high resolution LSFM imaging. The ratio between EdU+ and TO-PRO-3+ cells indicates the growth rate of each layer. These values are then used in our simulations.

	$\rho_{\text{TO-PRO-3}}$ [10^6 cells/mL]		ρ_{EdU} [10^6 cells/mL]		$\rho_{\text{EdU}} / \rho_{\text{TO-PRO-3}}$		Relative growth (Epidermis / Dermis)
	Epidermis	Dermis	Epidermis	Dermis	Epidermis	Dermis	
Untreated E51	1.10	0.82	0.094	0.094	0.085	0.115	0.743
Control E60	1.21	0.72	0.064	0.044	0.053	0.062	0.860

Supplementary Table 4. Values of the mathematical model effective parameters used in simulations. The parameters related to the material properties and the growth model are explained in Supplementary Notes 4 and 6, respectively. For the parameters not listed in the table, the following constant values are used: $k_2 = 0$, $G^{+/-}_{N,\text{dermis}} = 0.3$, $G^{+/-}_{N,\text{epidermis}} = 0$, $\lambda^{\text{EGF}}_{N/T,\text{dermis}} = 0$, $\lambda^{\text{EGF}}_{N,\text{epidermis}} = 0$, $t_1 = 0$. In all cases $G^{+/-}_{T,\text{epidermis}}$ is set to $0.8G^{+/-}_{T,\text{dermis}}$ to comply with measurements from real samples (Supplementary Table 3). The values in bold are found through Bayesian optimisation followed by rounding.

		$E_{\text{epidermis/dermis}}$	$\nu_{\text{epidermis/dermis}}$	$G^{+/-}_{T,\text{dermis}}$	$G^{+/-}_{T,\text{epdermis}}$	$\lambda^{\text{EGF}}_{T,\text{epidermis}}$	t_2	k_1
Control E64 (Fig. 4A, ED Fig. 9C & 10B, SD Fig 12A)	upper jaw	**2.5** / 1	**0.15** / **0.27**	**1.1** / **0.8**	0.88 / 0.64	0	0	**300**
	lower jaw	**2.5** / 1	**0.15** / **0.27**	**1.0** / **0.9**	0.80 / 0.72	0	0	**500**
Control E75 (ED Fig. 10C)	upper jaw	3 / 1	0.05 / 0.27	1.1 / 0.8	0.88 / 0.64	0	0	300
	lower jaw	3 / 1	0.05 / 0.27	1.0 / 0.9	0.80 / 0.72	0	0	500
Treated 3x injection E64 (Fig. 4B & ED Fig. 10D)	upper jaw	**3** / 1	**0.35** / 0.27	1.1 / 0.8	0.88 / 0.64	**0.07**	9	300
	lower jaw	**3** / 1	**0.35** / 0.27	1.0 / 0.9	0.80 / 0.72	**0.07**	9	500
Treated 3x injection E75 (ED Fig. 10F)	upper jaw	3.5 / 1	0.075 / 0.27	1.1 / 0.8	0.88 / 0.64	0.07	9	300
	lower jaw	3.5 / 1	0.075 / 0.27	1.0 / 0.9	0.80 / 0.72	0.07	9	500
Treated 5x injection E75 (ED Fig. 10E)	upper jaw	4 / 1	0.1 / 0.27	1.1 / 0.8	0.88 / 0.64	0.07	15	300
	lower jaw	4 / 1	0.1 / 0.27	1.0 / 0.9	0.80 / 0.72	0.07	15	500
No-fibers E64 (SD Fig. 12B)	upper jaw	2.5 / 1	0.15 / 0.27	1.1 / 0.8	0.88 / 0.64	0	0	0
	lower jaw	2.5 / 1	0.15 / 0.27	1.0 / 0.9	0.80 / 0.72	0	0	0

Supplementary Table 5. Summary of embryonic samples used for LSFM

**Box/ Clutch Number**	**Sample numbers for treatment types and doses**						
	Treatment time (Embryonic day)	Fixed at start of treatment	PBS control	EGF 0.625 µg	EGF 1.25 µg	EGF 2 µg	Total
**1**	E48 - E57	3	3	3	3	-	12
**2**	E55 - E64	6	4	5	5	5	25
**3**	E51 - E60	4	4	4	4	4	20
**4**	E55 - E64	2	5	4	4	4	19

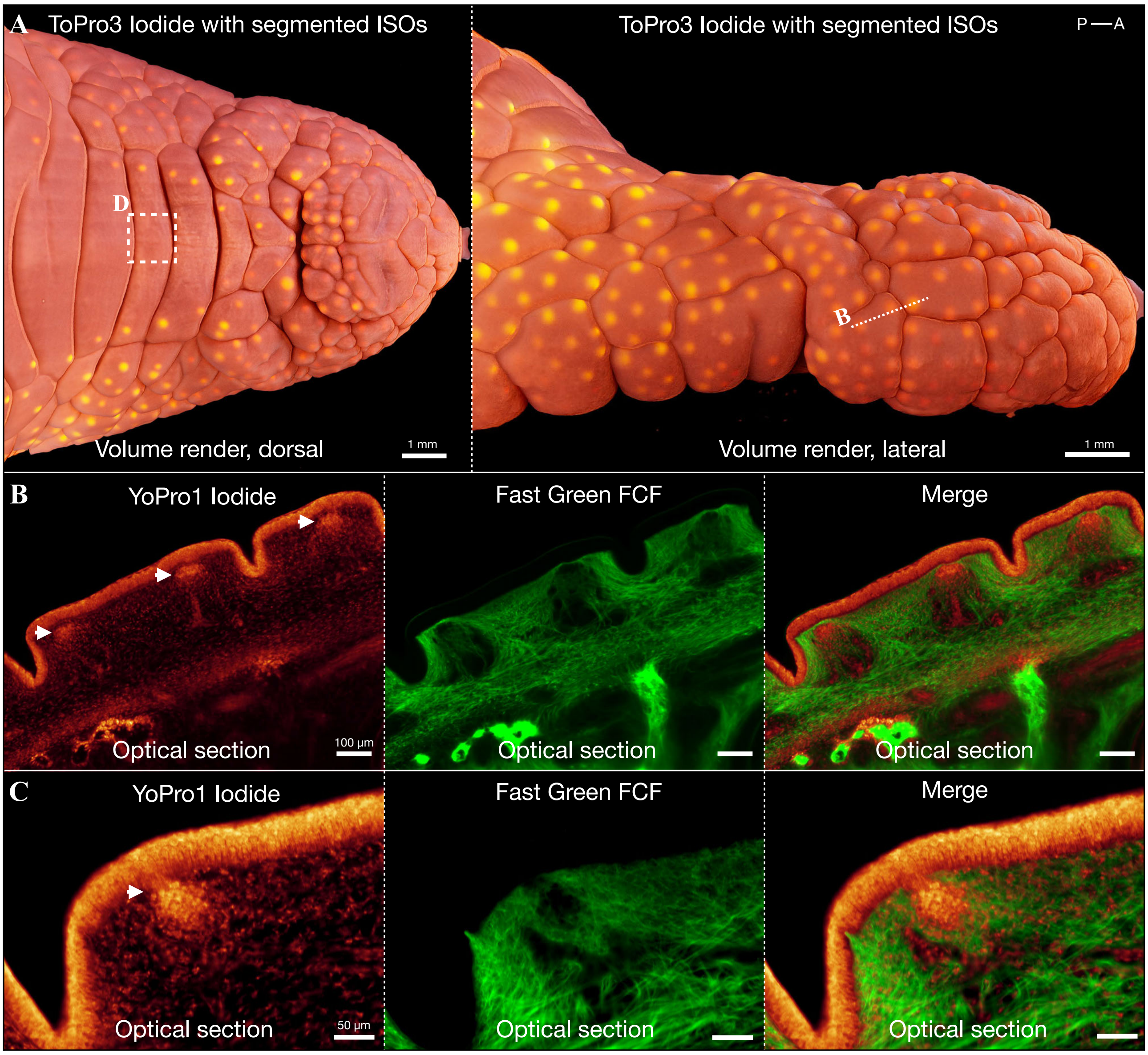

Supplementary Figure 1. LSFM imaging of integumentary multi-sensory organs (ISOs) and collagen fibres in crocodile head scales at E63. (A) Surface render of a 3D volume acquired with TO-PRO-3 Iodide nuclear staining in an embryonic Nile crocodile sample; the high cell density of ISOs allows their segmentation (shown in yellow). P = posterior; A = anterior. (**B**) An optical section of crocodile upper jaws labelled with YO-PRO-1 Iodide (orange, nuclear staining) and Fast Green FCF (green, collagen staining) shows that ISOs consist of a dermal cell aggregation (white arrow) within a collagen-rich dermis. (**C**) Further magnified optical sections reveal the attachment of developing ISOs to the overlying epidermal cells. Replicates of LSFM samples with nuclear staining are listed in Supplementary Table 5. The spatial variation of collagen architecture was validated in 8 individual biological replicates with either LSFM or confocal microscopy.

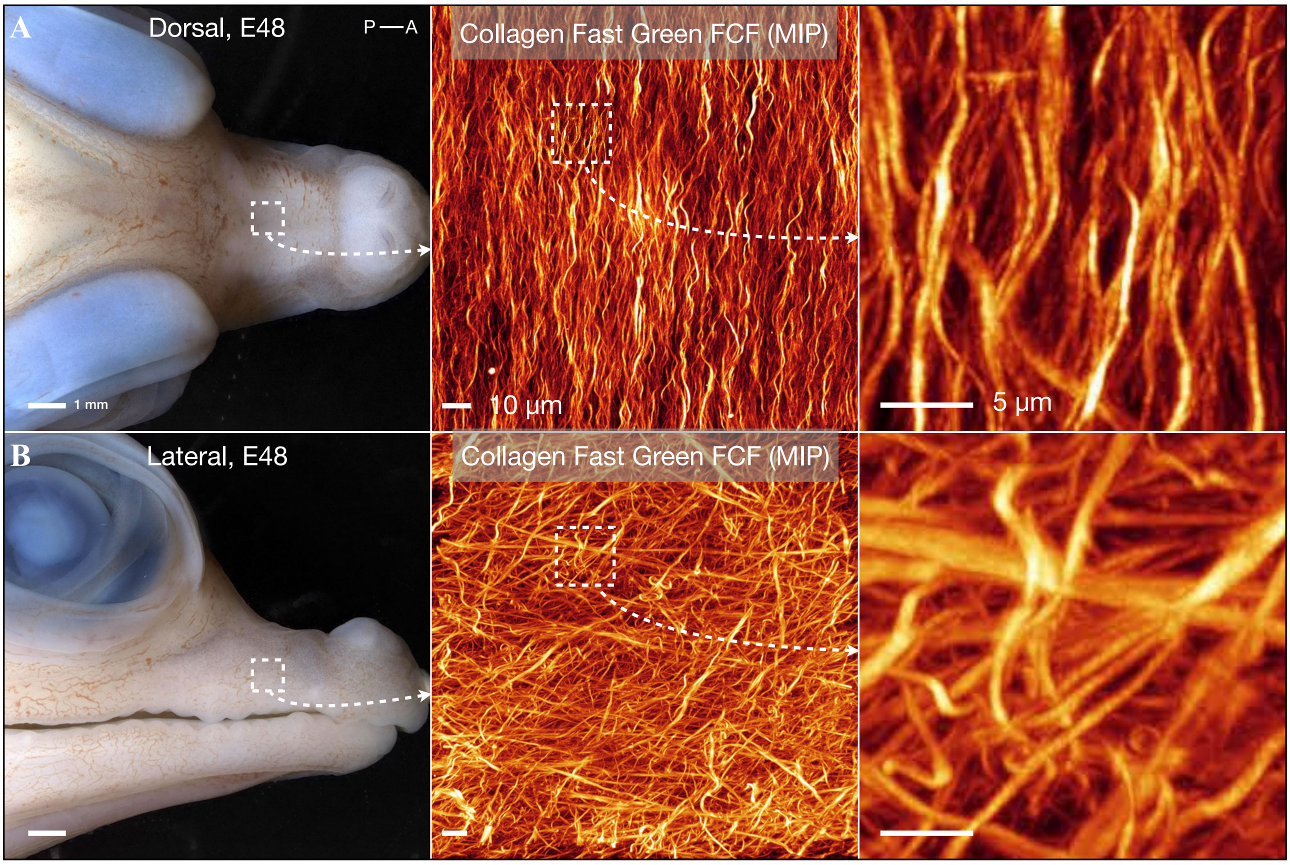

Supplementary Figure 2. Confocal imaging of collagen architecture prior to head scale emergence at E48. Confocal microscopy was used to capture fluorescent signal from Fast Green FCF staining in high resolution[22]. **(A)** Collagen architecture on the dorsal jaw dermis is highly organised, with fibres consistently running perpendicular to the direction of jaw elongation. **(B)** Collagen architecture on the lateral jaw surface lacks a single consistent dominant orientation of fibres. P = posterior; A = anterior. The spatial variation of 3D collagen network architecture was validated in 8 individual biological replicates with either LSFM or confocal microscopy.

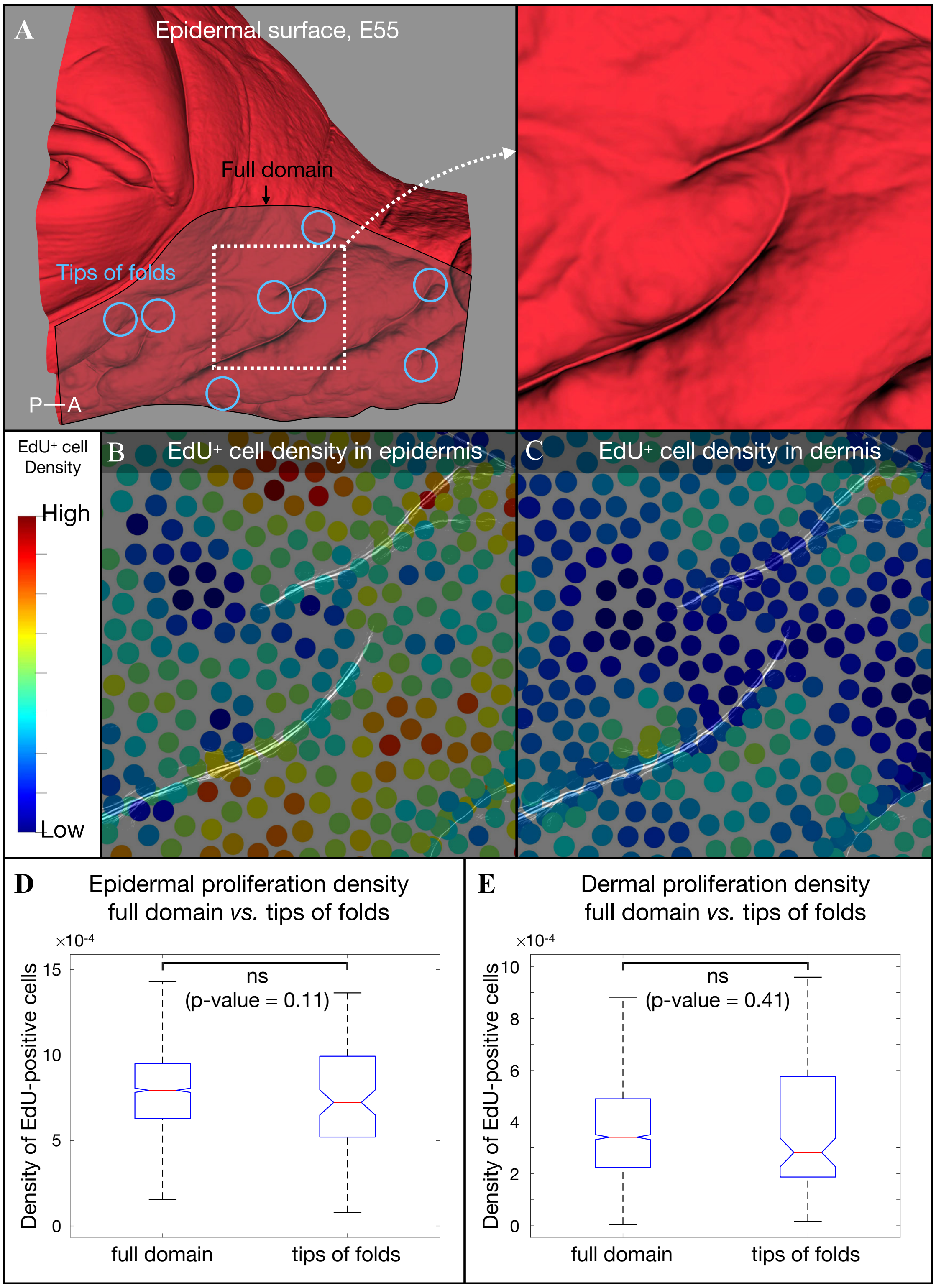

Supplementary Figure 3. Proliferation is not localised at the tips of propagating scale folds. (**A**) Scale fold network of an E55 embryonic crocodile upper jaw (epidermal surface reconstruction from LSFM imaging of TO-PRO-3 nuclear staining). **(B-C)** The density of proliferating (EdU+) cells is shown for both the epidermis and dermis (see 'Methods'). **(D-E)** The density of EdU^{+} cells is not significantly larger at tips of propagating folds (n=119 sampling volumes within the blue circles in panel A) than in the full domain (n=1907 sampling volumes within the grey outline of panel A). Hence, there is no local enhancement of cell proliferation associated with the tip of propagating folds. Medians (red lines), 25-75 percentiles (blue boxes), and ranges (black brackets) are shown. Statistical significance is calculated using one-way ANOVA.

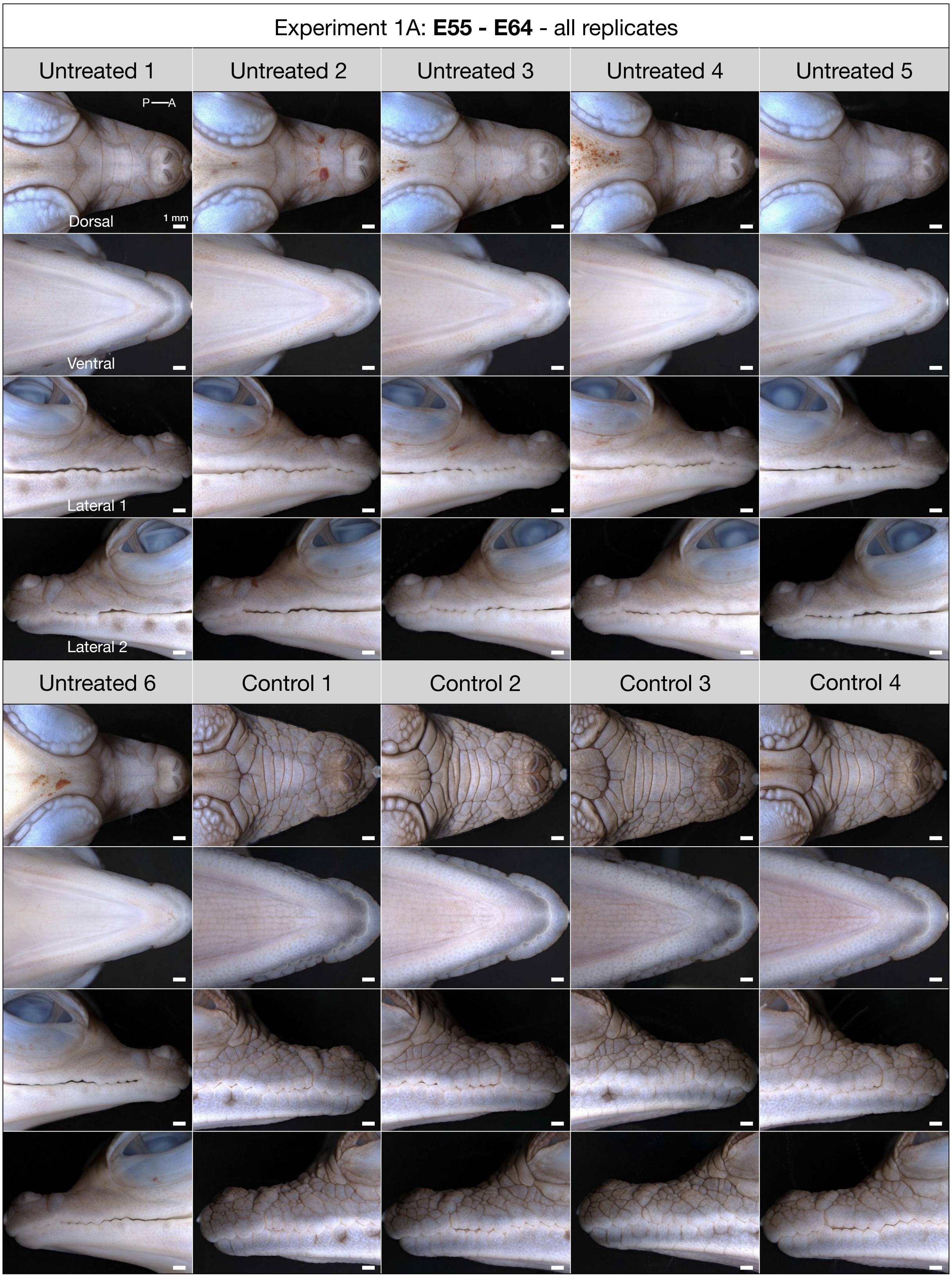

Supplementary Figure 4. All replicates from intravenous EGF treatments during embryonic head scale patterning. Results obtained with different treatment times and EGF doses are shown. Each experiment (numbered 1, 2, or 3) corresponds to a different timing of injections (see Fig. 2A in the Main Text) and corresponds to an individual clutch of eggs for which some embryos remain untreated and are fixed at day 0 of the treatment, whereas others are control samples injected with PBS or treated samples injected 3 times with different doses of EGF (indicated). P = posterior; A = anterior. The figure continues on the following 12 pages.

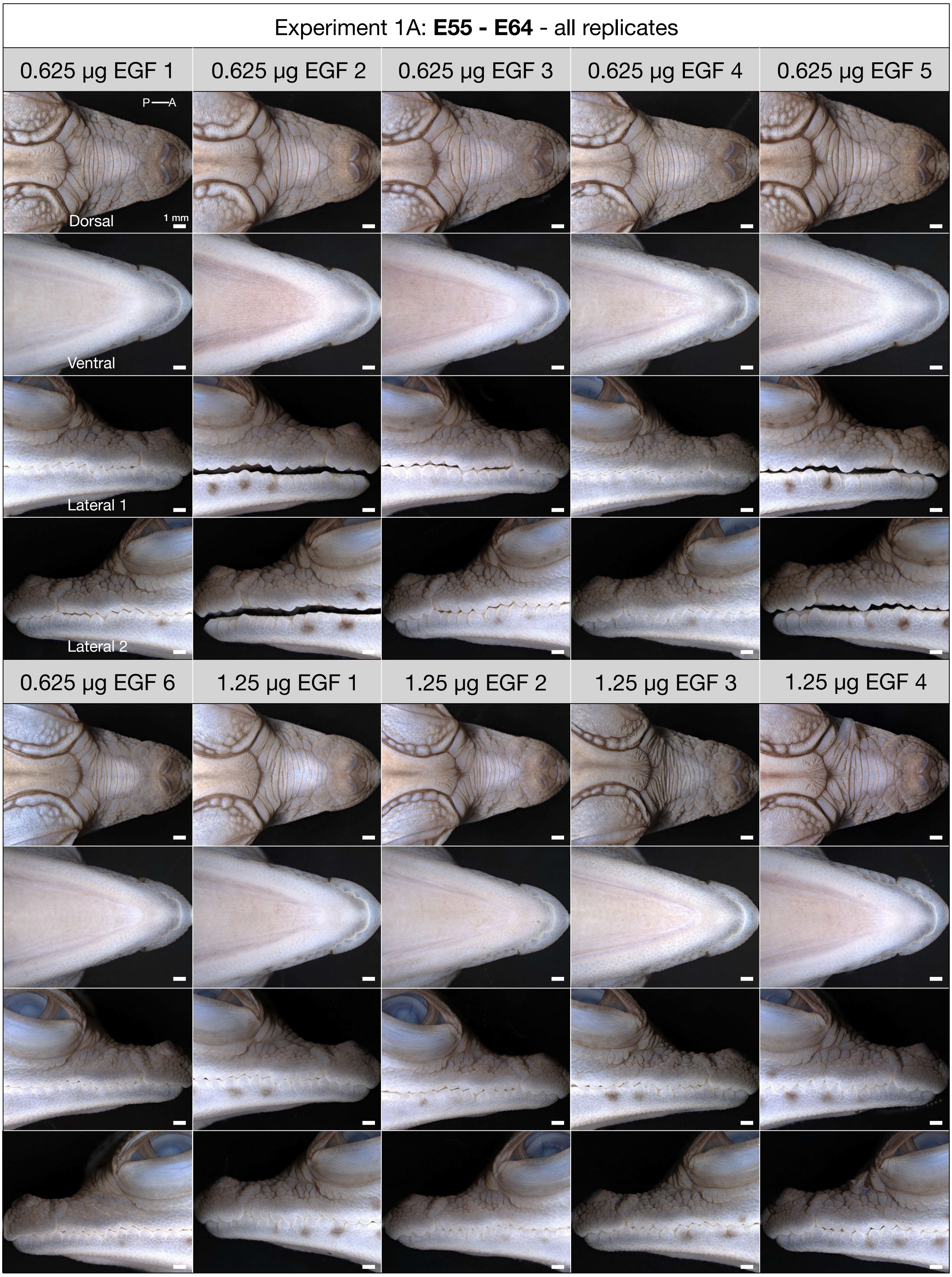

Supplementary Figure 4 — Continued.

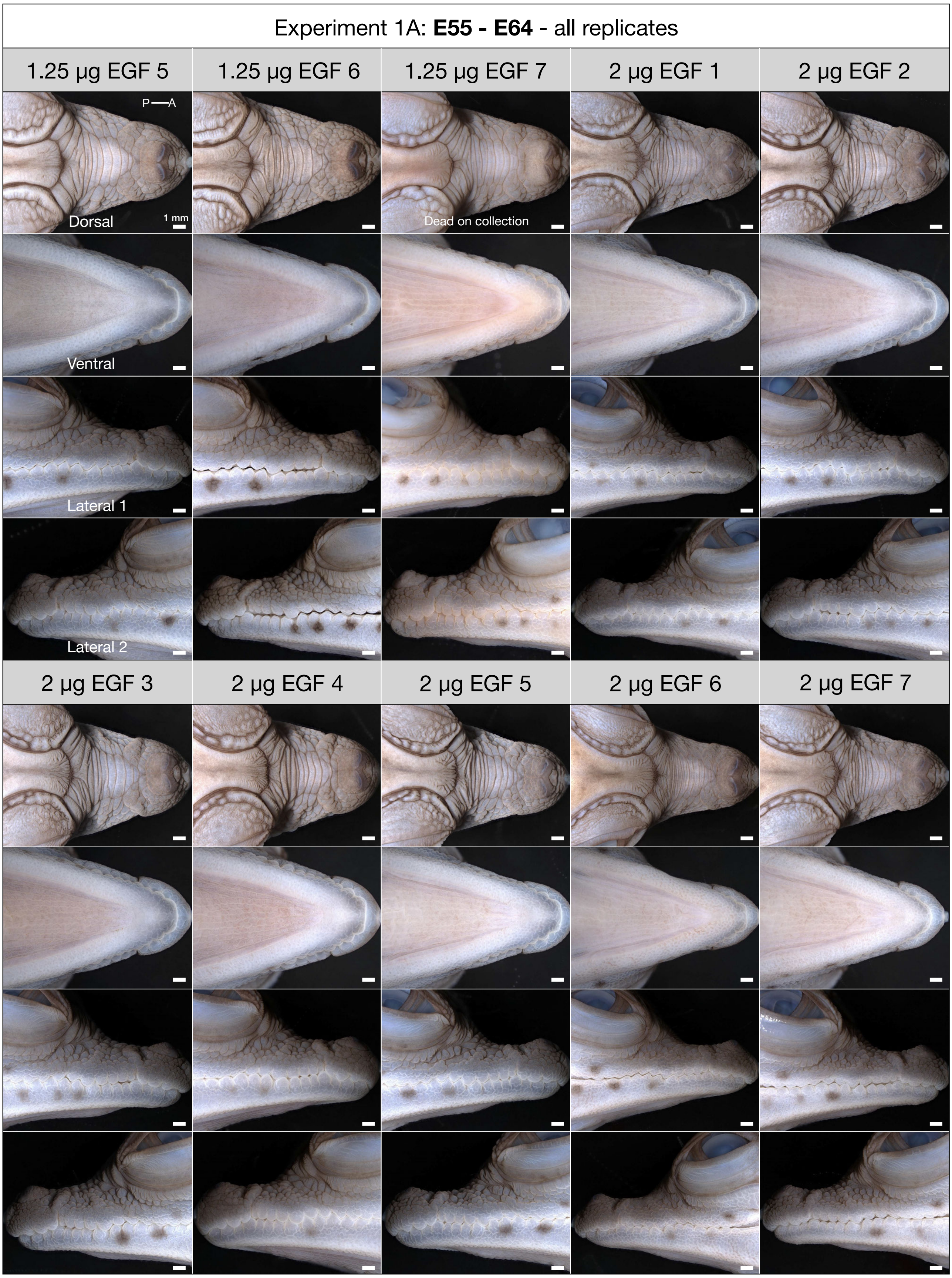

Supplementary Figure 4 — Continued.

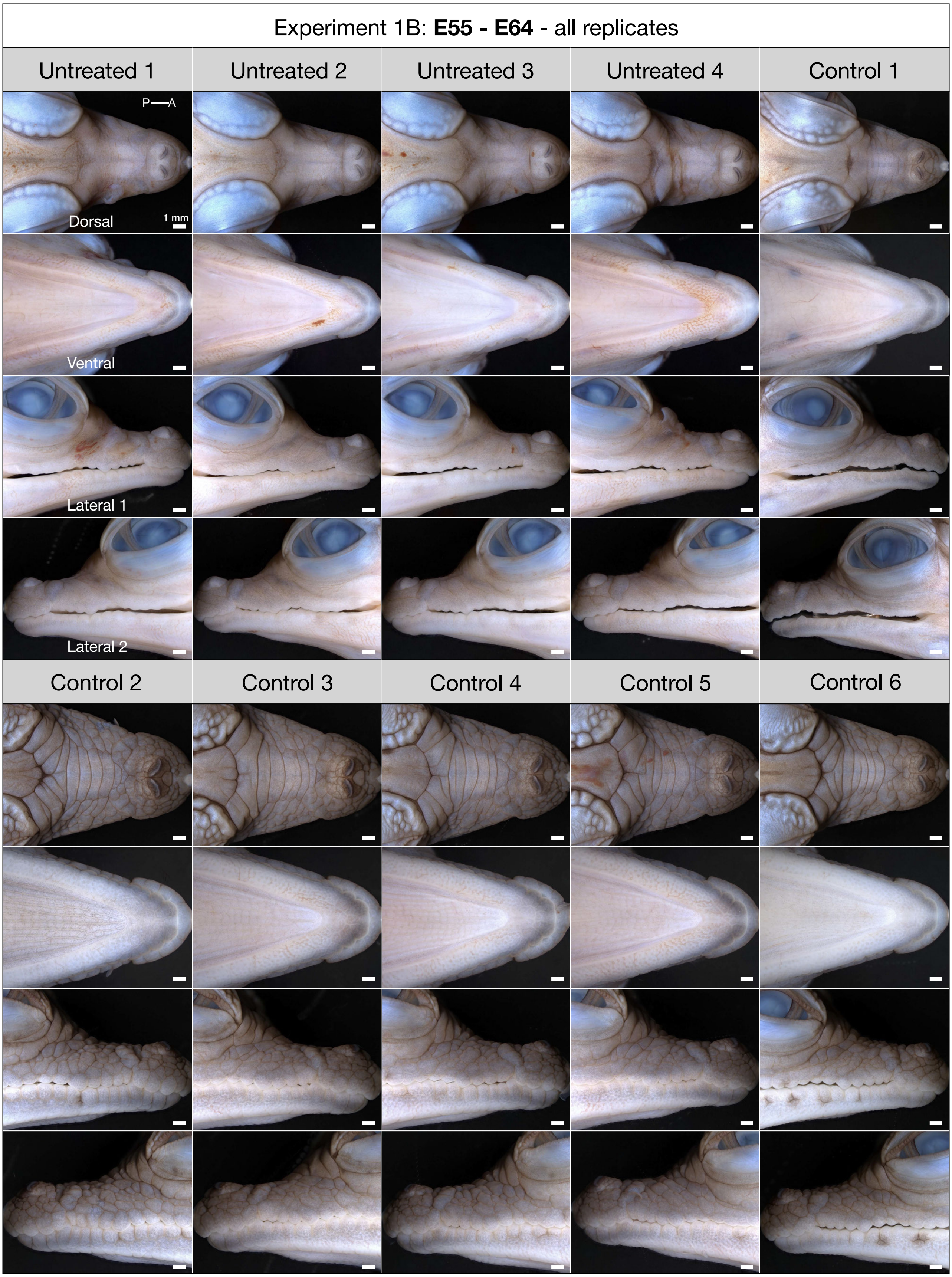

Supplementary Figure 4 — Continued.

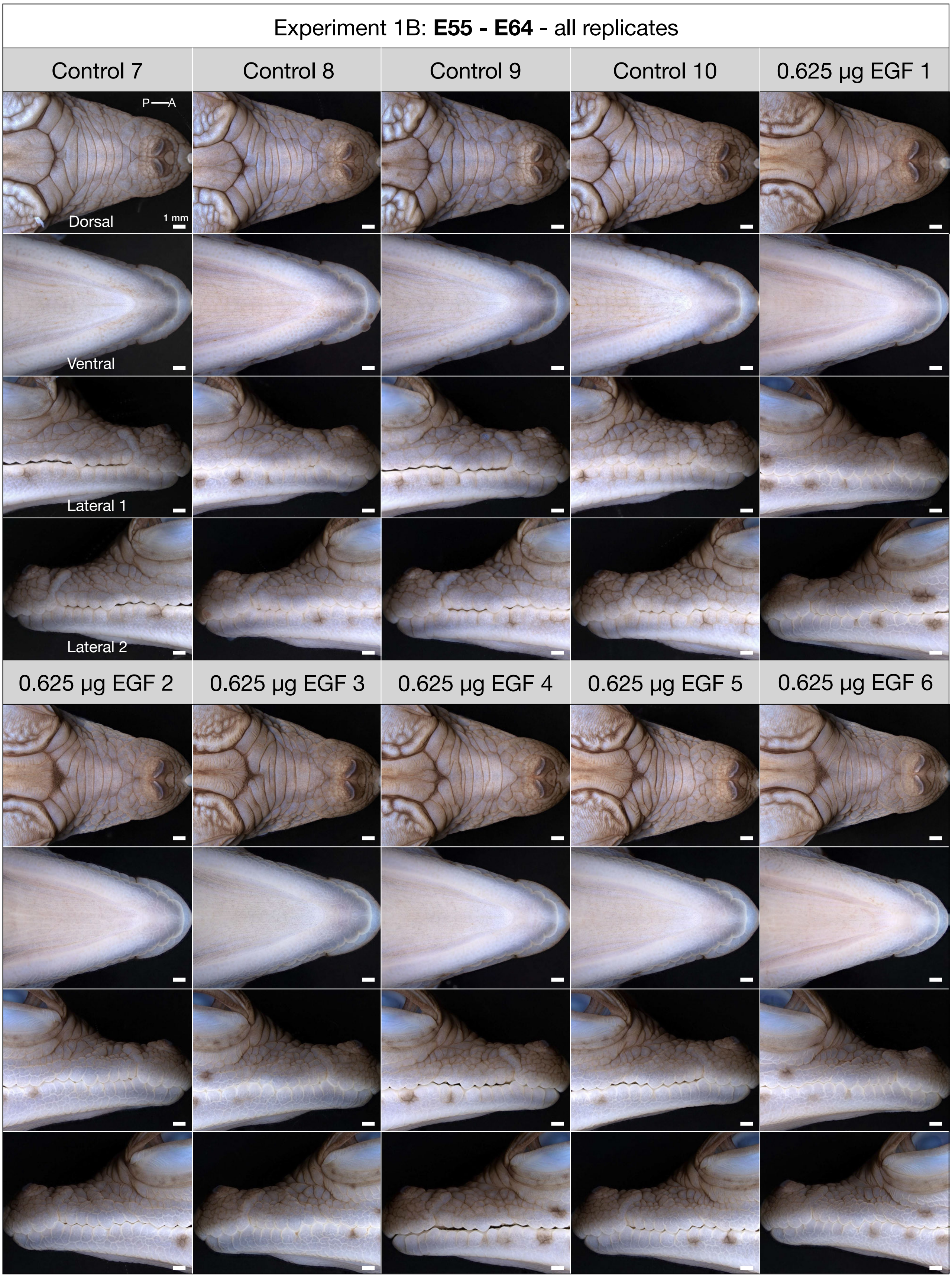

Supplementary Figure 4 — Continued.

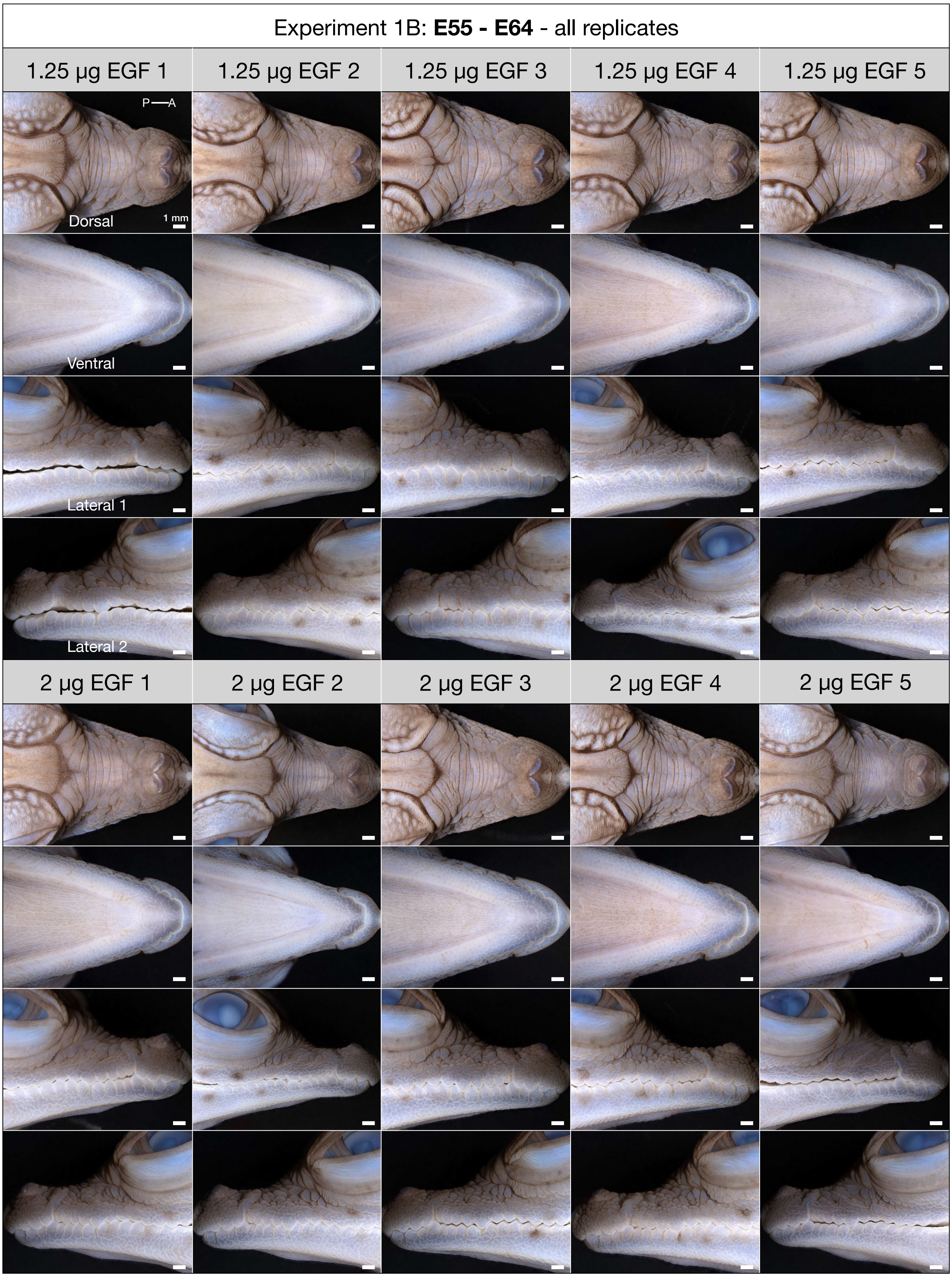

Supplementary Figure 4 — Continued.

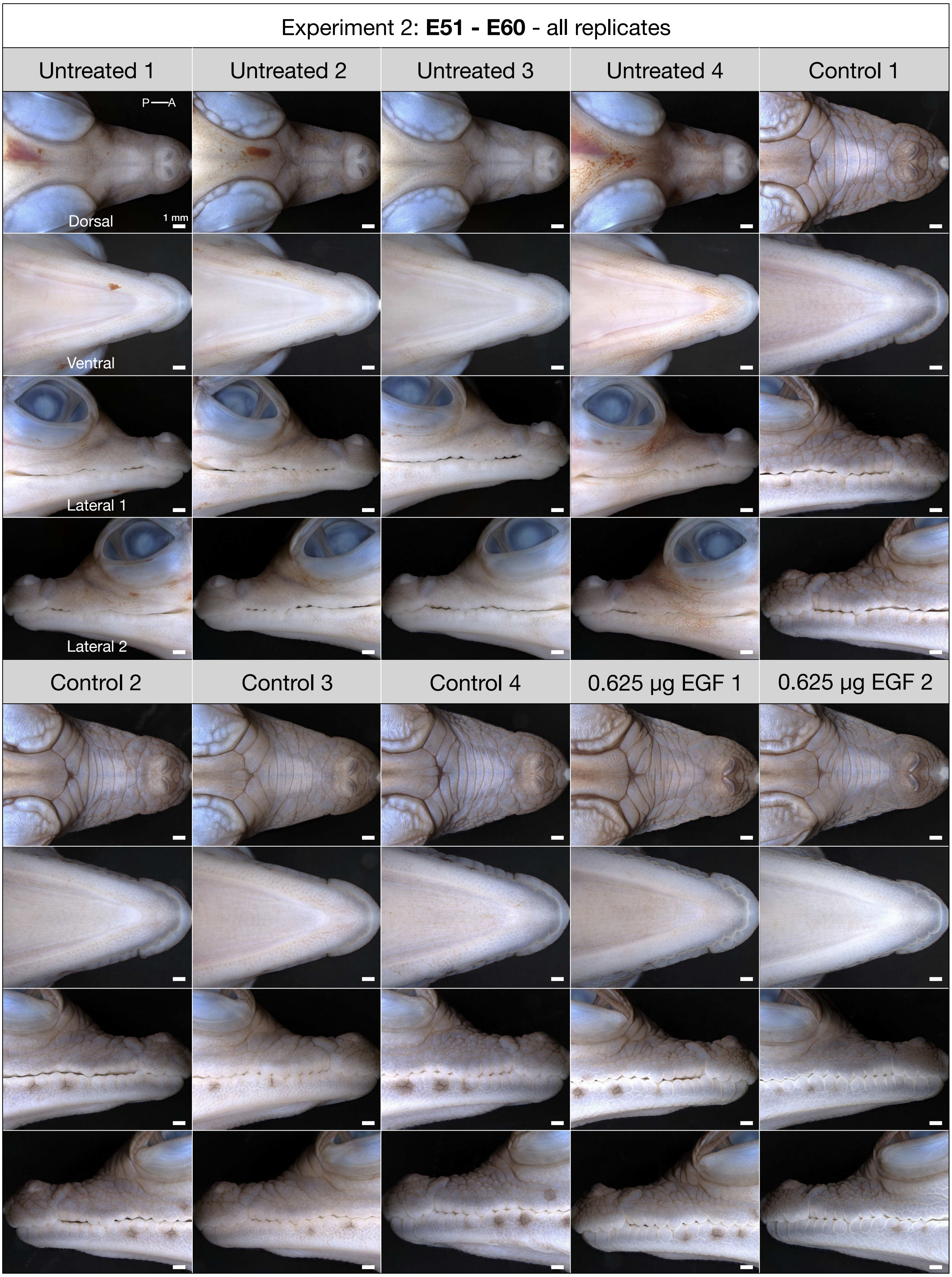

Supplementary Figure 4 — Continued.

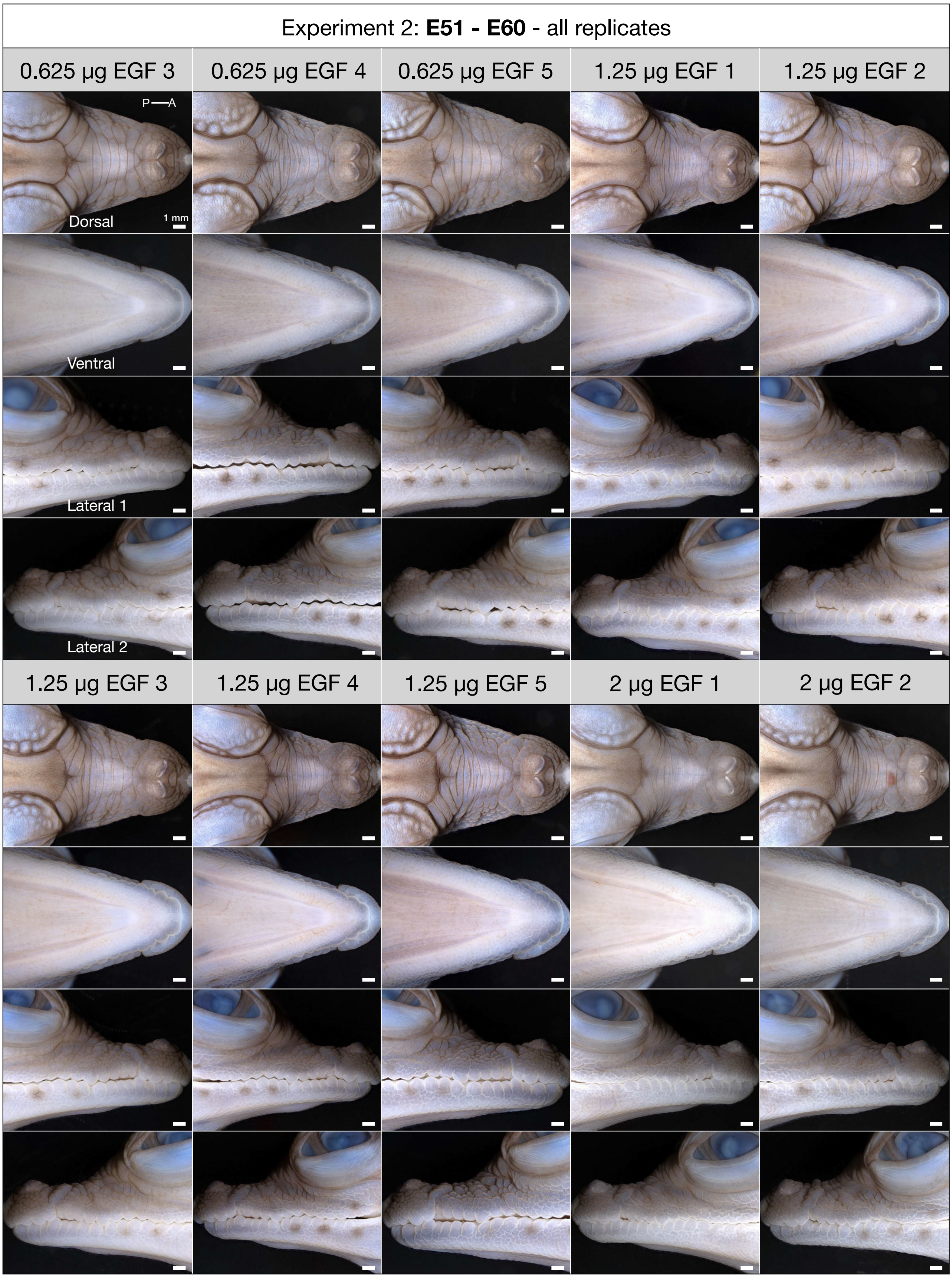

Supplementary Figure 4 — Continued.

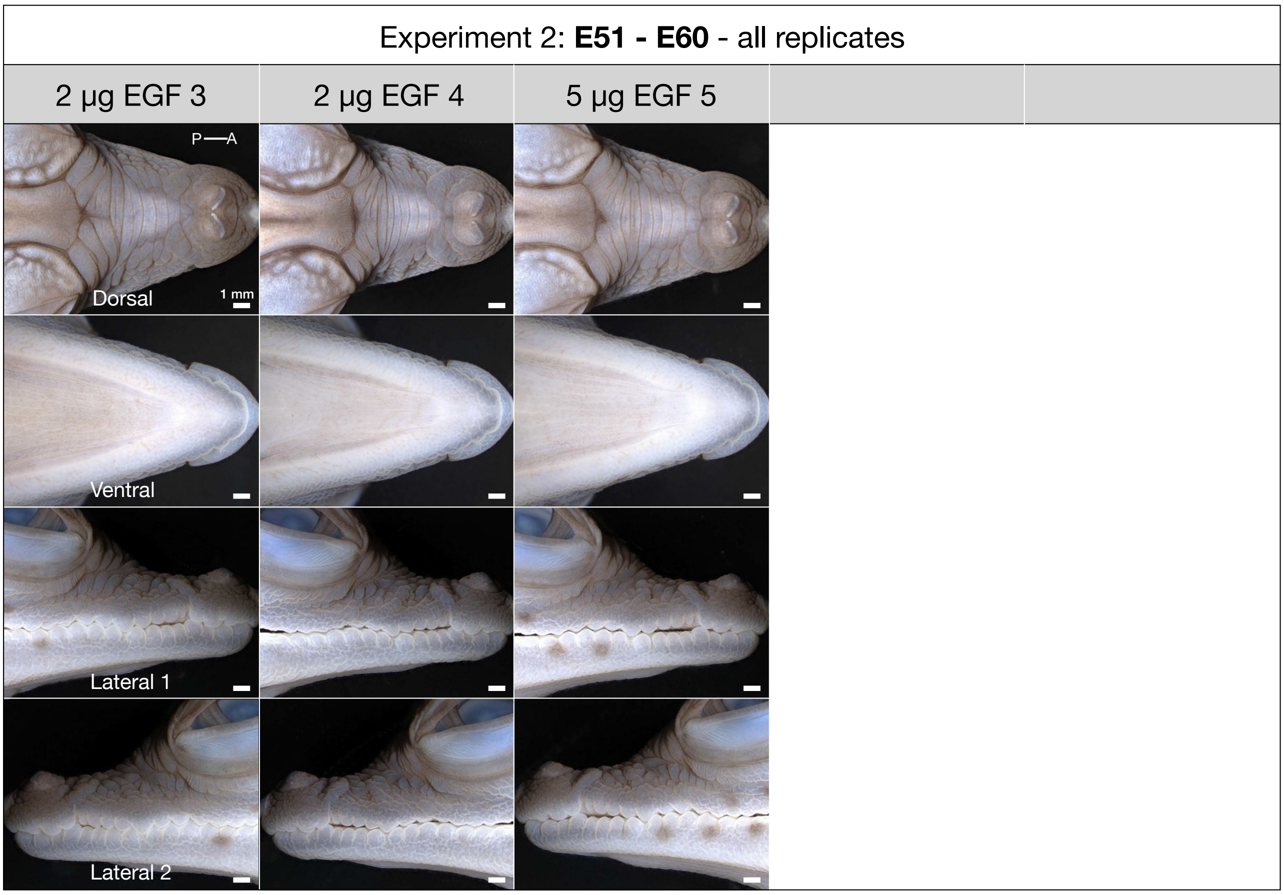

Supplementary Figure 4 — Continued.

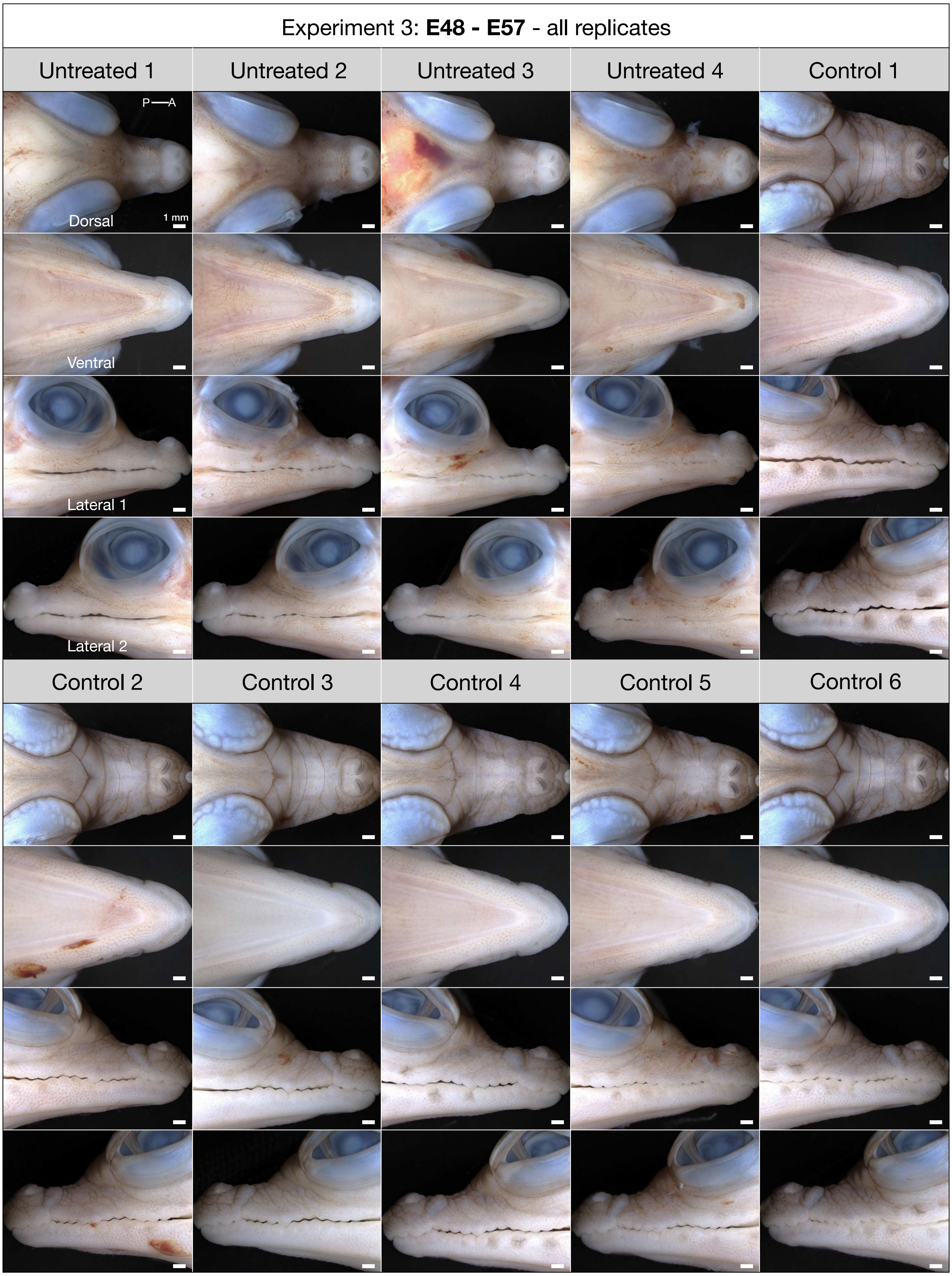

Supplementary Figure 4 — Continued.

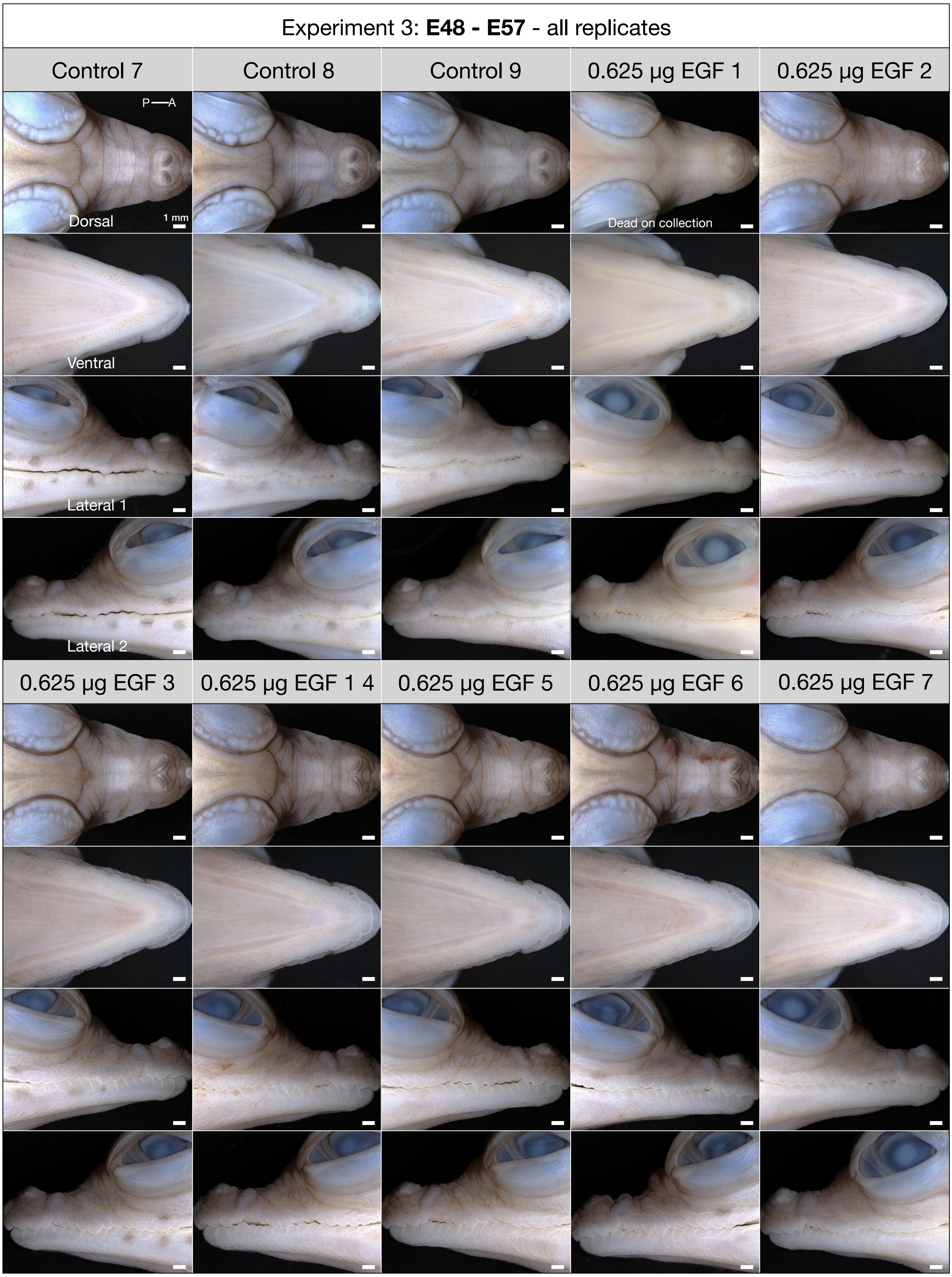

Supplementary Figure 4 — Continued.

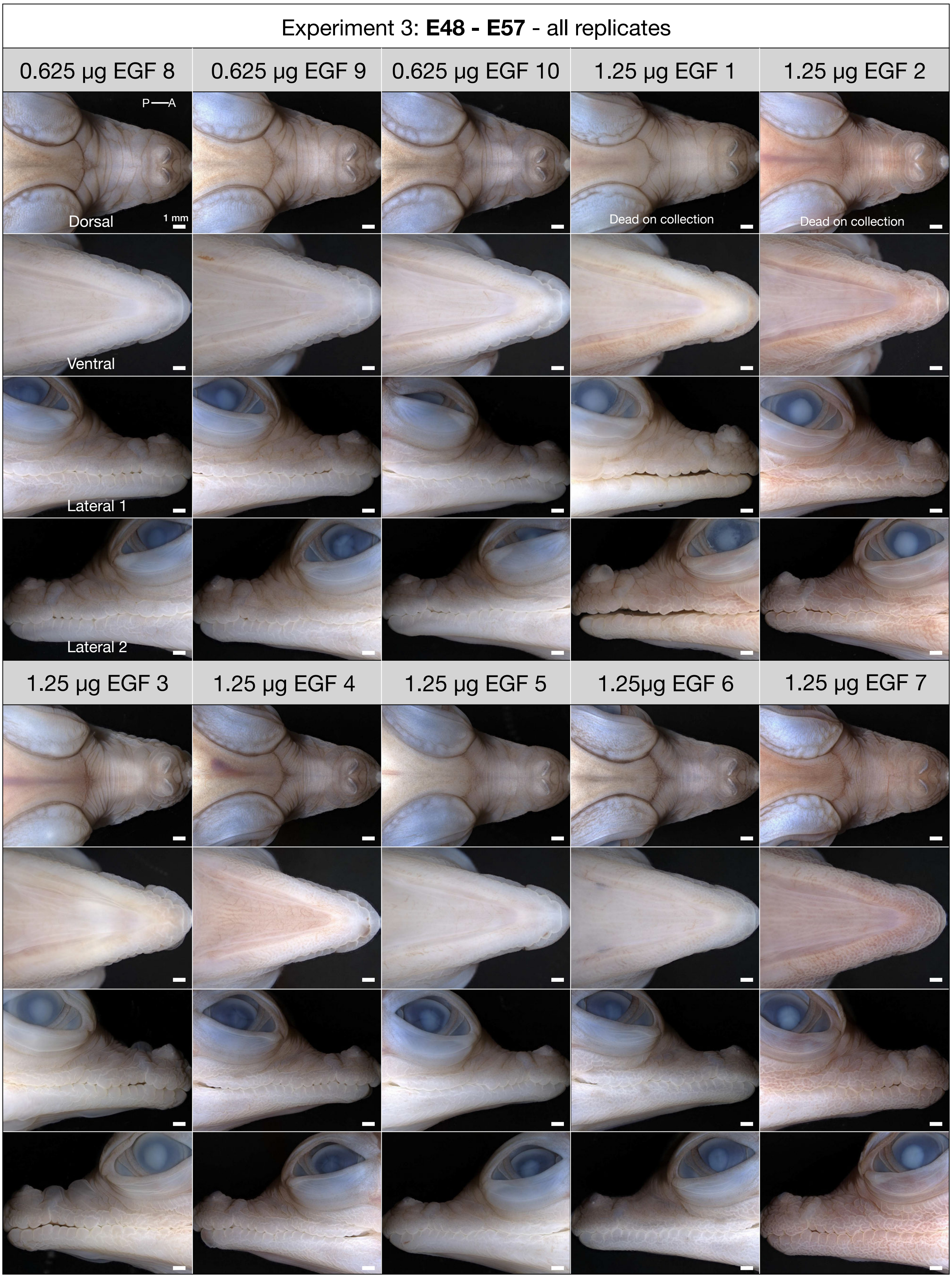

Supplementary Figure 4 — Continued.

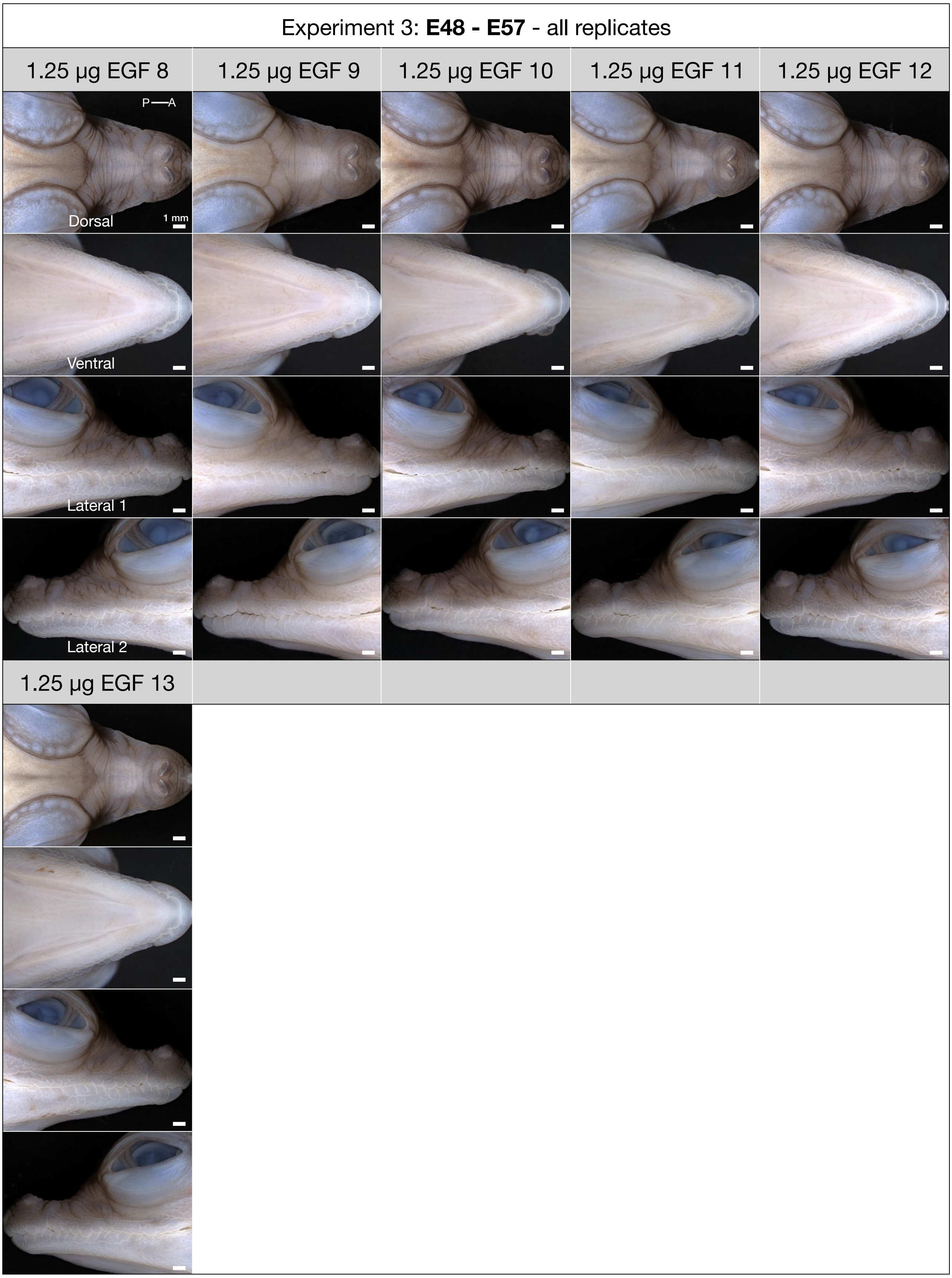

Supplementary Figure 4 — Continued.

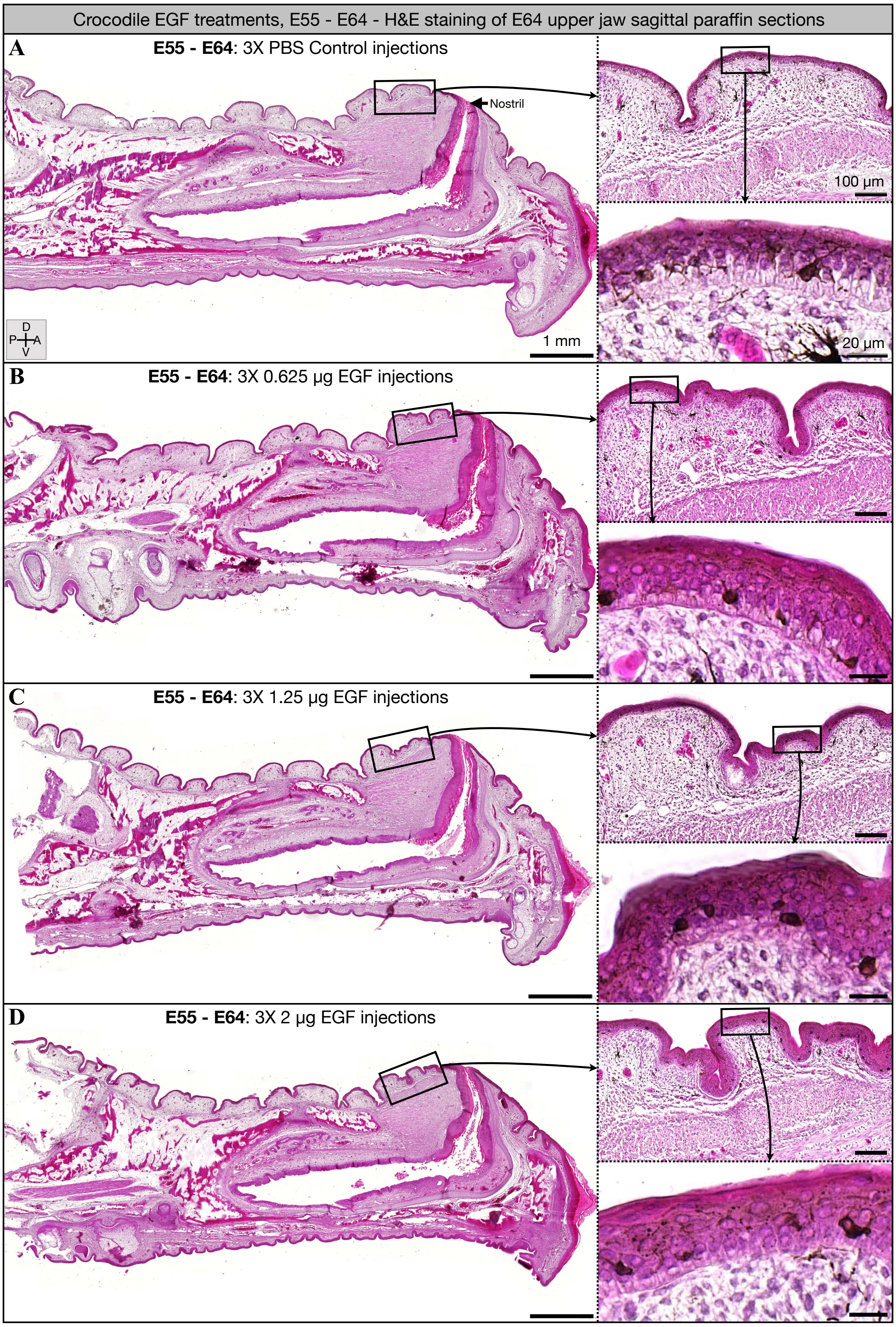

Supplementary Figure 5. The effect of EGF treatment upon head scale patterning is dose-dependent — histological sections. H&E-stained paraffin sections from the upper jaw taken in a sagittal plane from both control (PBS-injected) samples and EGF-treated samples, injected between E55 and E64. Increased doses of EGF (from (A) to (D)) result in increased epidermal thickness and keratinisation. Right panels show successive expanded views. The axes in panel A show the posterior (P) - anterior (A) and the dorsal (D) - ventral (V) orientations. Histological sections were prepared from two biological replicates for each treatment and further validated with LSFM (see Supplementary Table 5 for details of replicates).

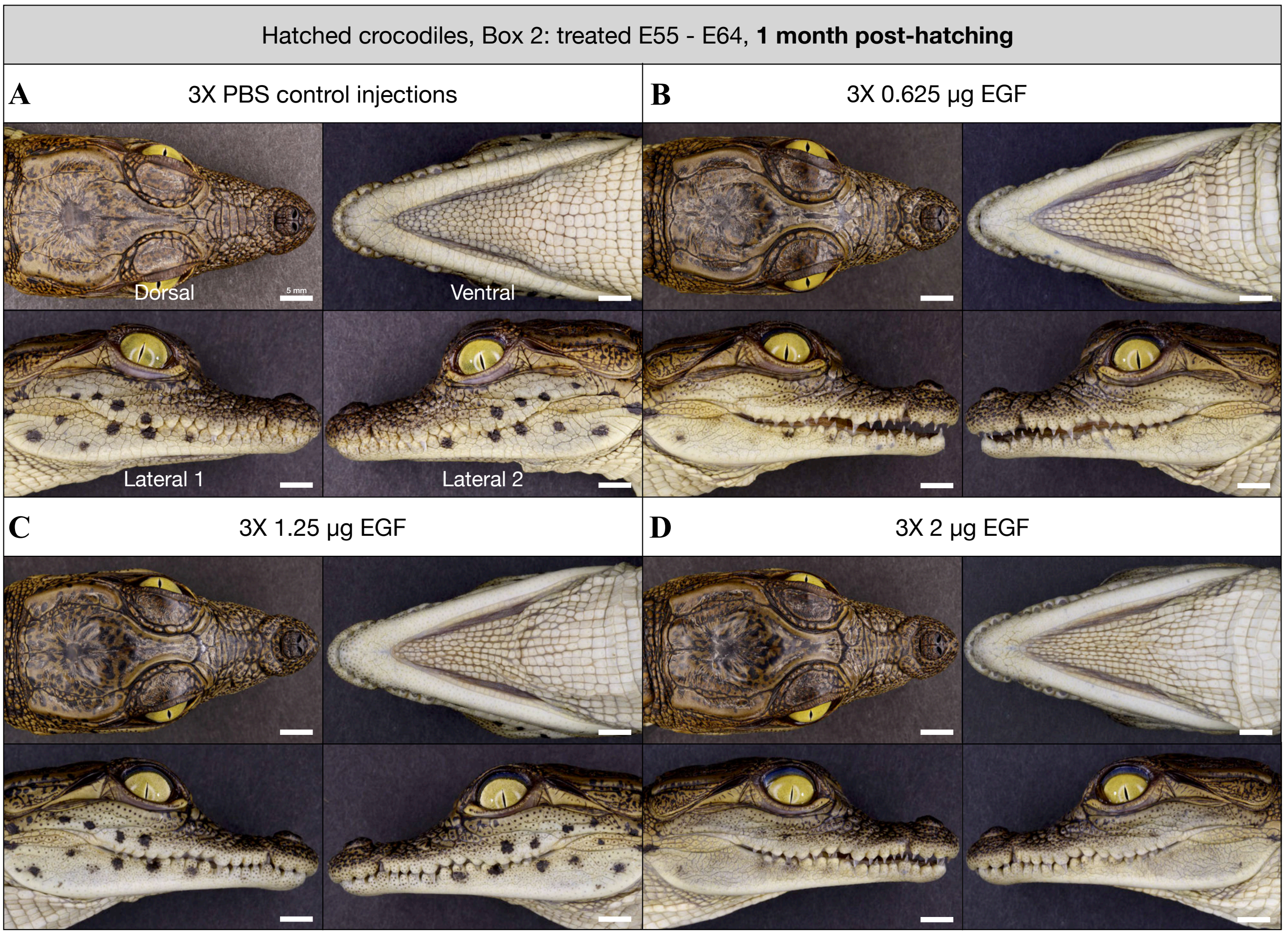

Supplementary Figure 6. Post-embryonic effect (at 1 mph) of *in-ovo* EGF treatment. (A-J) Nile crocodile embryos treated at E55-E64 (A-D) or E51-E60 (E-J) with either PBS (as controls) or different doses of EGF were hatched and allowed to develop until 1 month post-hatching (mph). Crocodiles show an EGF dose-dependent increase in the number of (smaller) polygonal domains, at either time point. Note that some specimens (*e.g.*, panel H), treated with 2 µg EGF from E51 to E60, have their scale folds partially obliterated as if fusion of distinct head scale domains occurred because of excessive epidermal growth and keratinisation. The figure continues on the next page.

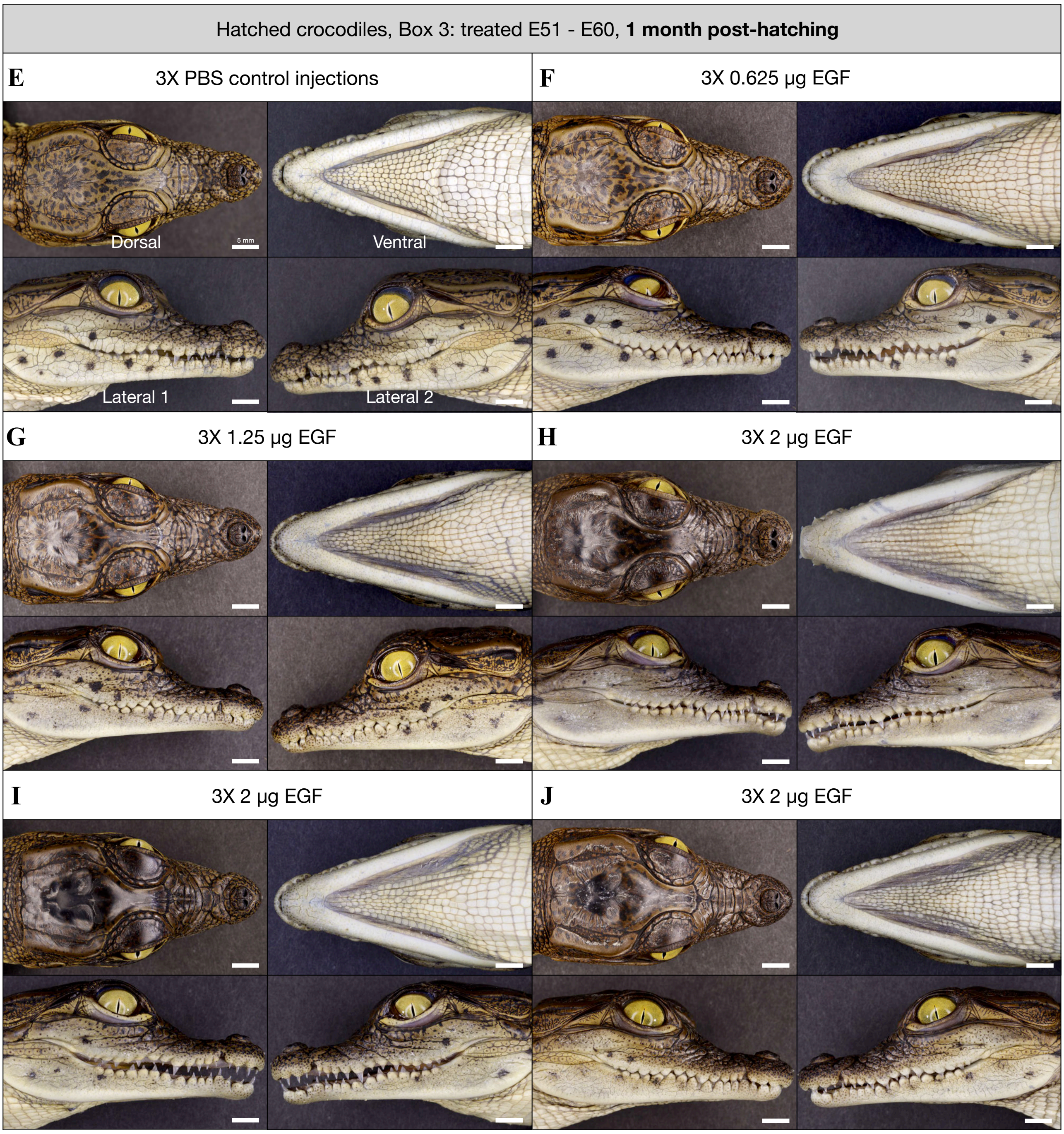

Supplementary Figure 6 — Continued.

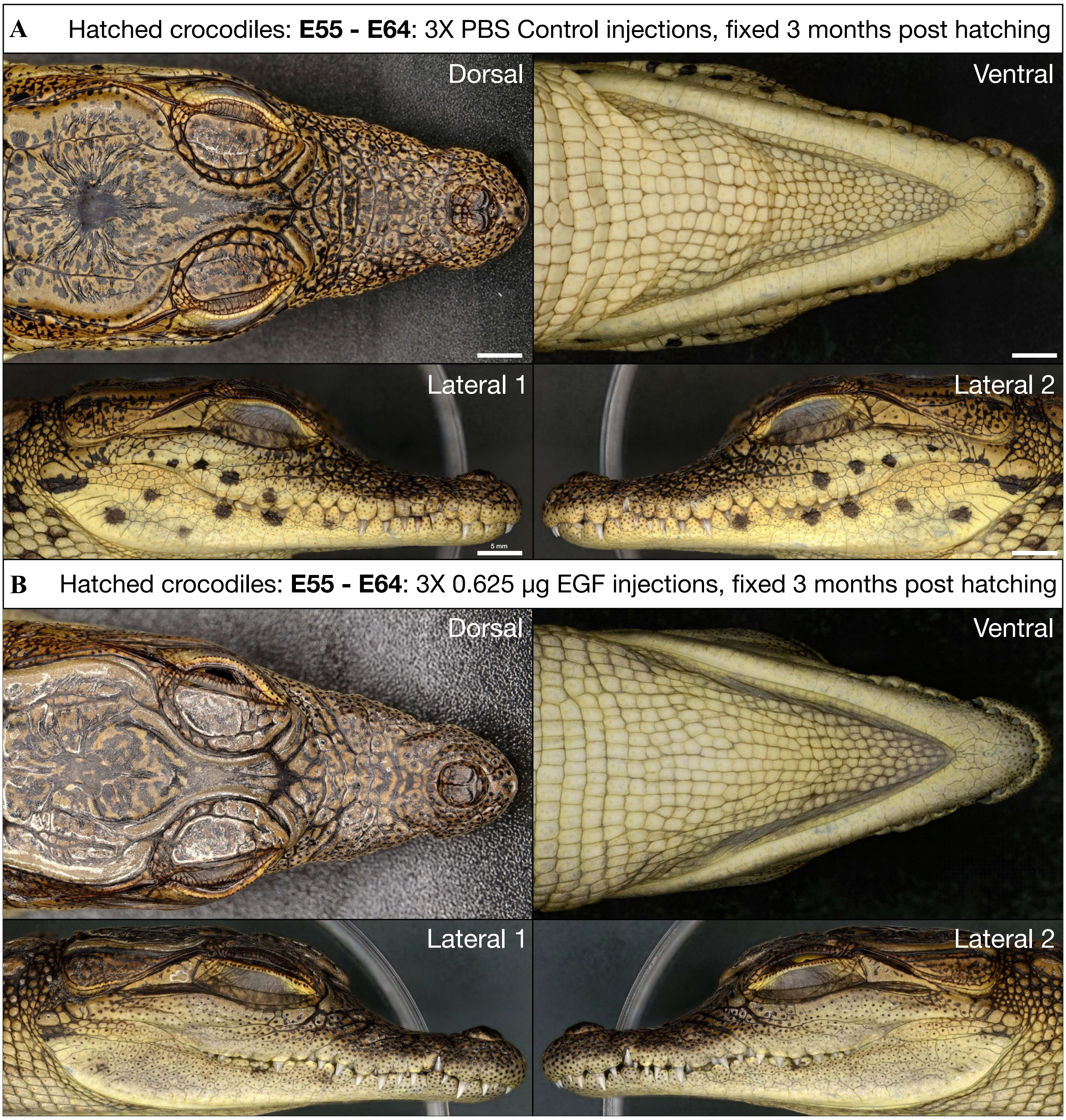

Supplementary Figure 7. Post-embryonic effect (at 3 mph) of *in-ovo* EGF treatment. (A-J) Nile crocodile embryos treated at E55-E64 (A-D) or E51-E60 (E-J) with either PBS (as controls) or different doses of EGF were hatched and allowed to develop until 3 month post-hatching (mph). Samples treated with EGF exhibit more numerous and smaller head scales (incidentally, exhibiting fewer ISOs per individual polygonal scale domain). Overall, the effect of EGF treatment on Nile crocodile head scale patterning remains visible after hatching. Individuals in (A) and (D) are shown in Extended Data Figure 6C and D. The figure continues on the next two page.

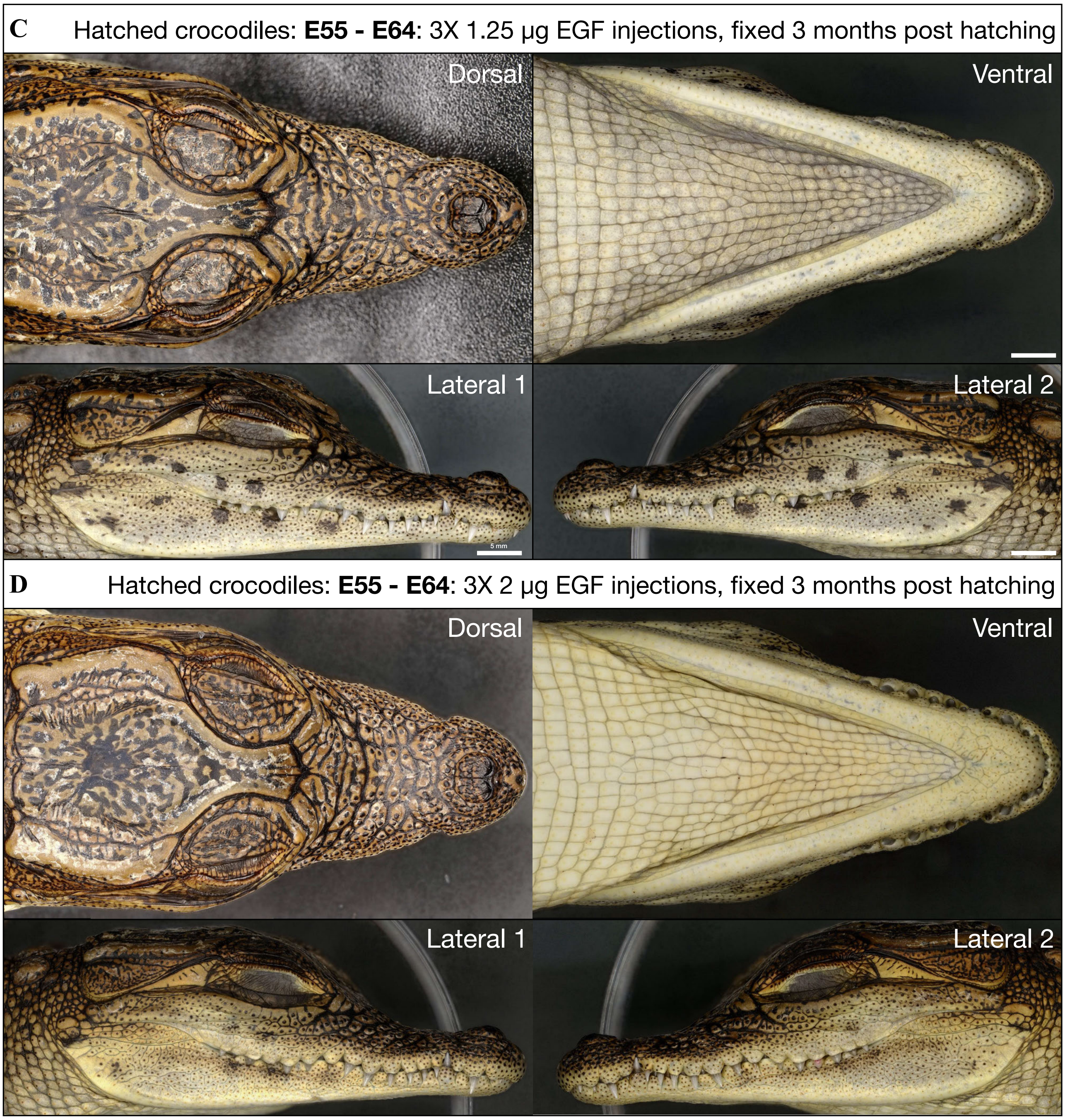

Supplementary Figure 7 — Continued.

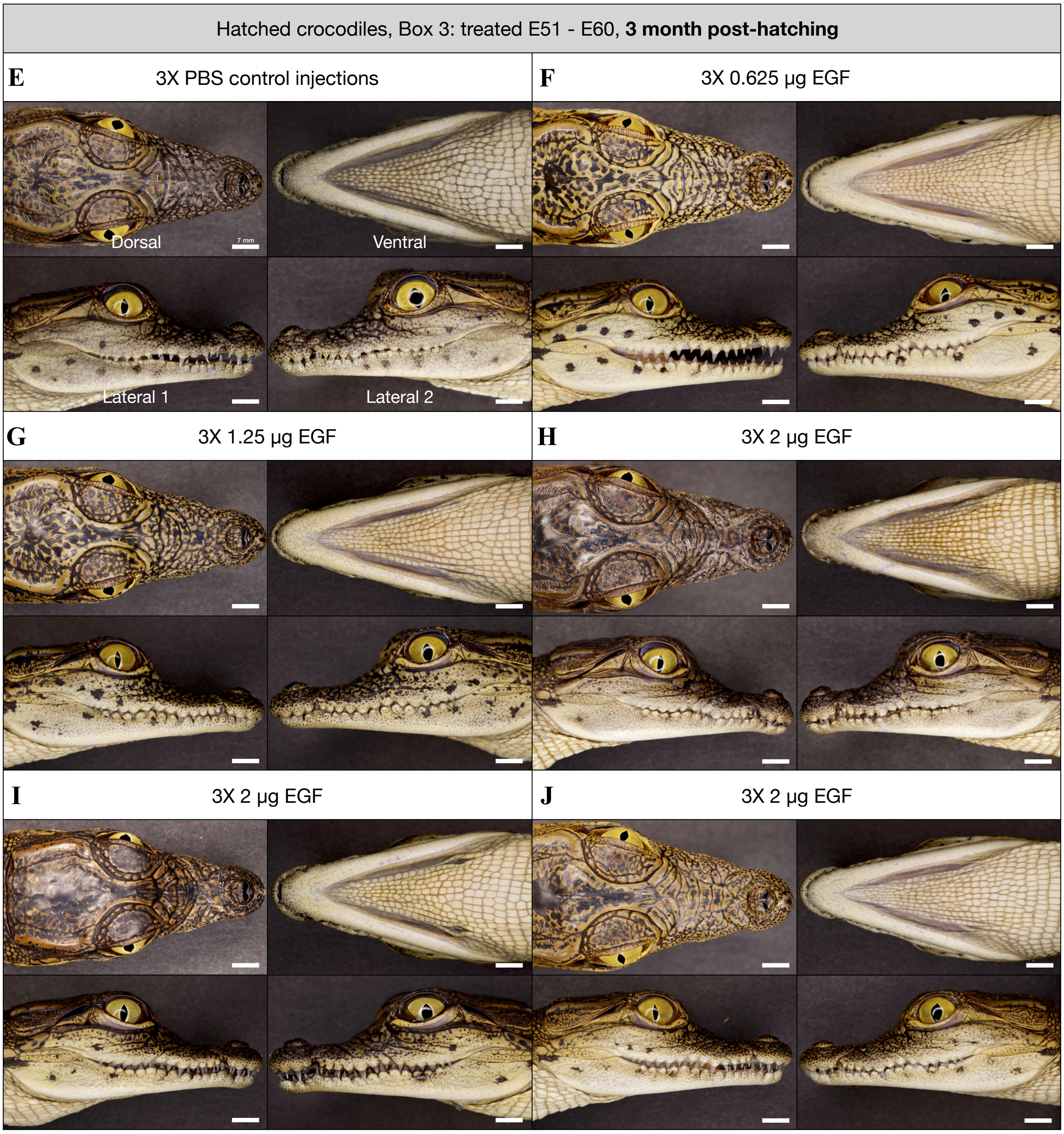

Supplementary Figure 7 — Continued.

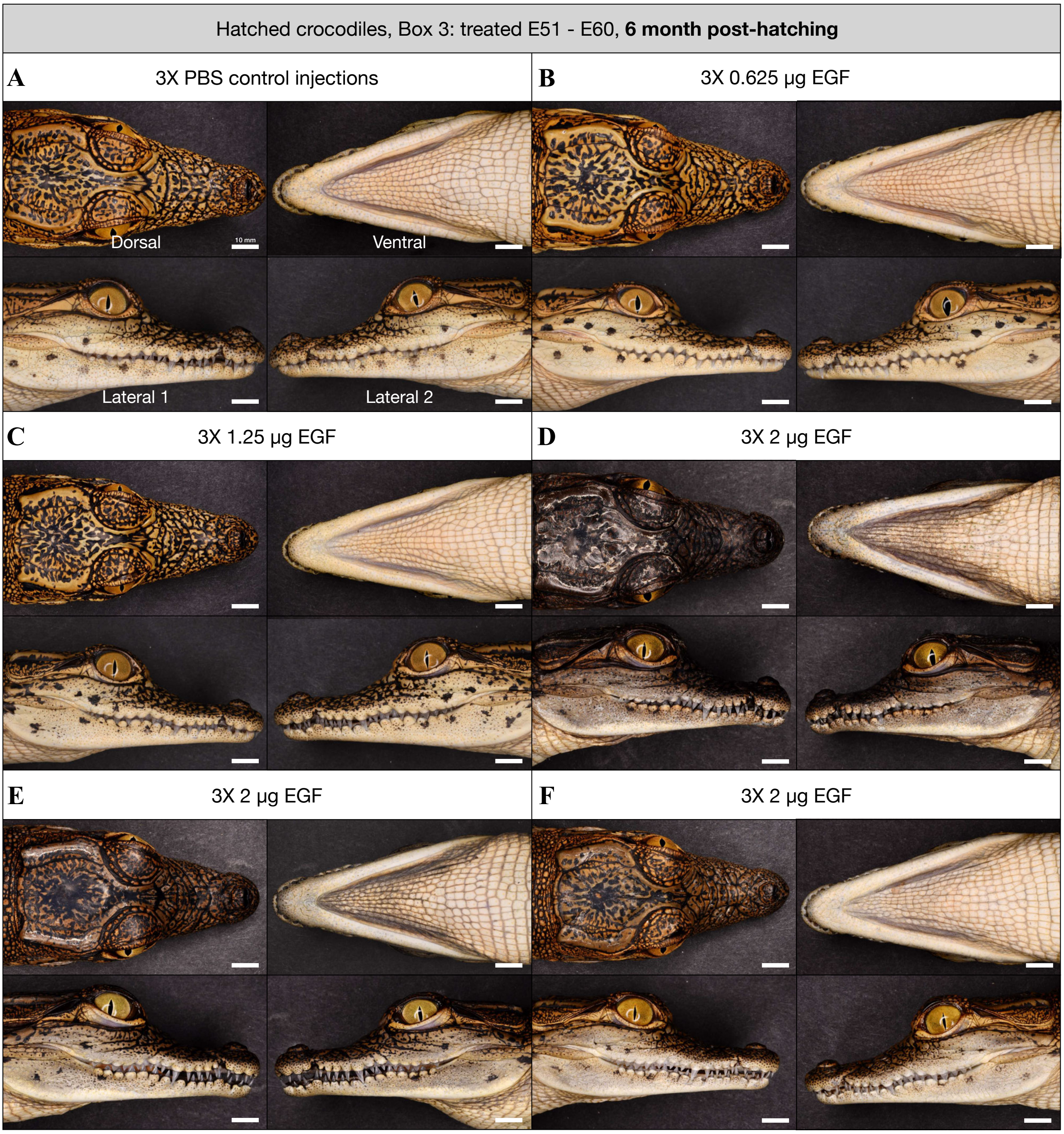

Supplementary Figure 8. Post-embryonic effect (6 mph) of *in-ovo* EGF treatment. (A-F) Control and EGF-treated Nile crocodiles injected at E51-E60 are shown at 6 mph. Crocodiles show an EGF dose-dependent increase in the number of (smaller) polygonal domains.

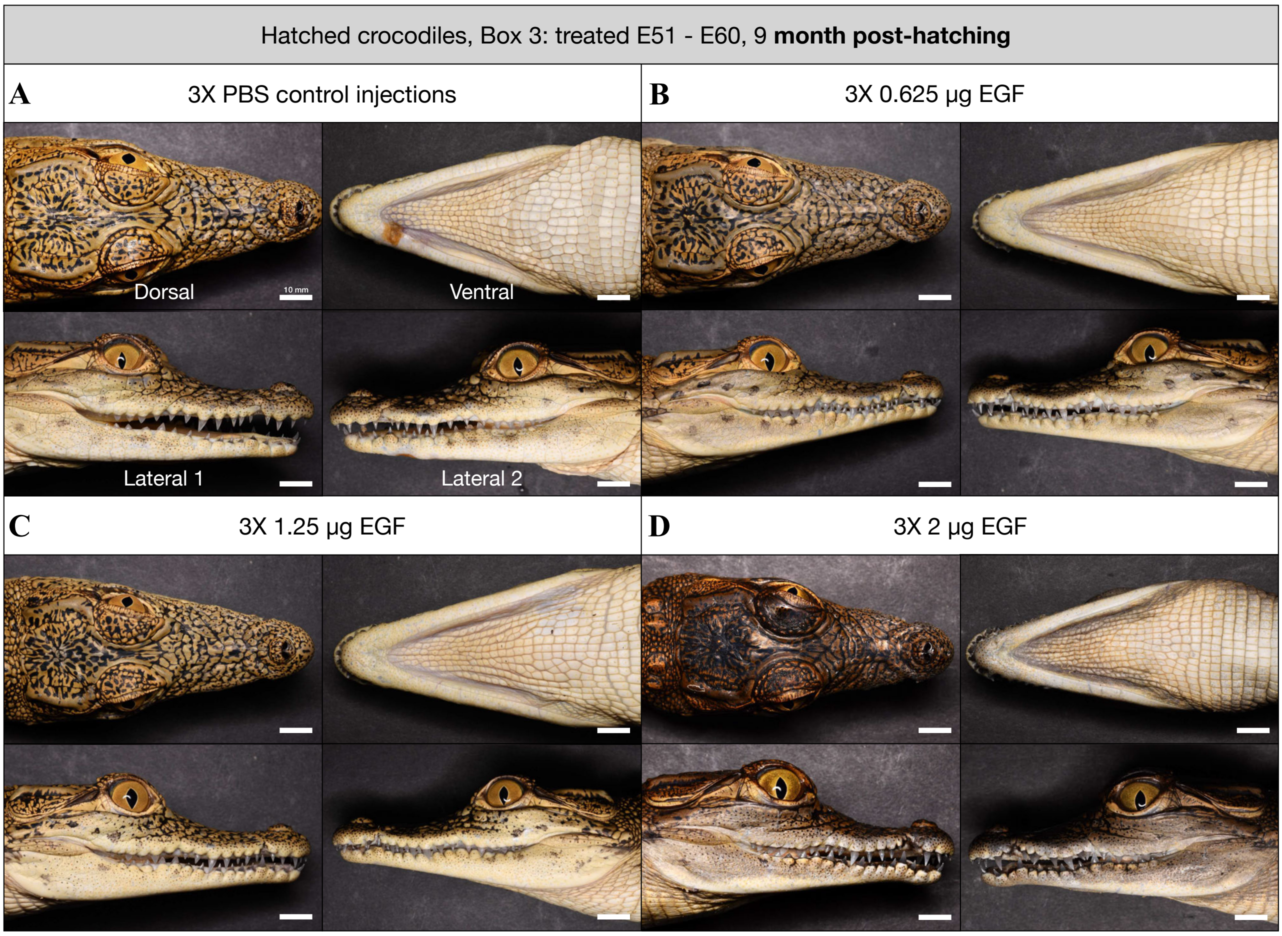

Supplementary Figure 9. Post-embryonic effect (9 mph) of in-ovo EGF treatment. (A-D) Control and EGF-treated crocodiles treated from E51-E60 are shown at 9 mph. Crocodiles show an EGF dose-dependent increase in the number of (smaller) polygonal domains.

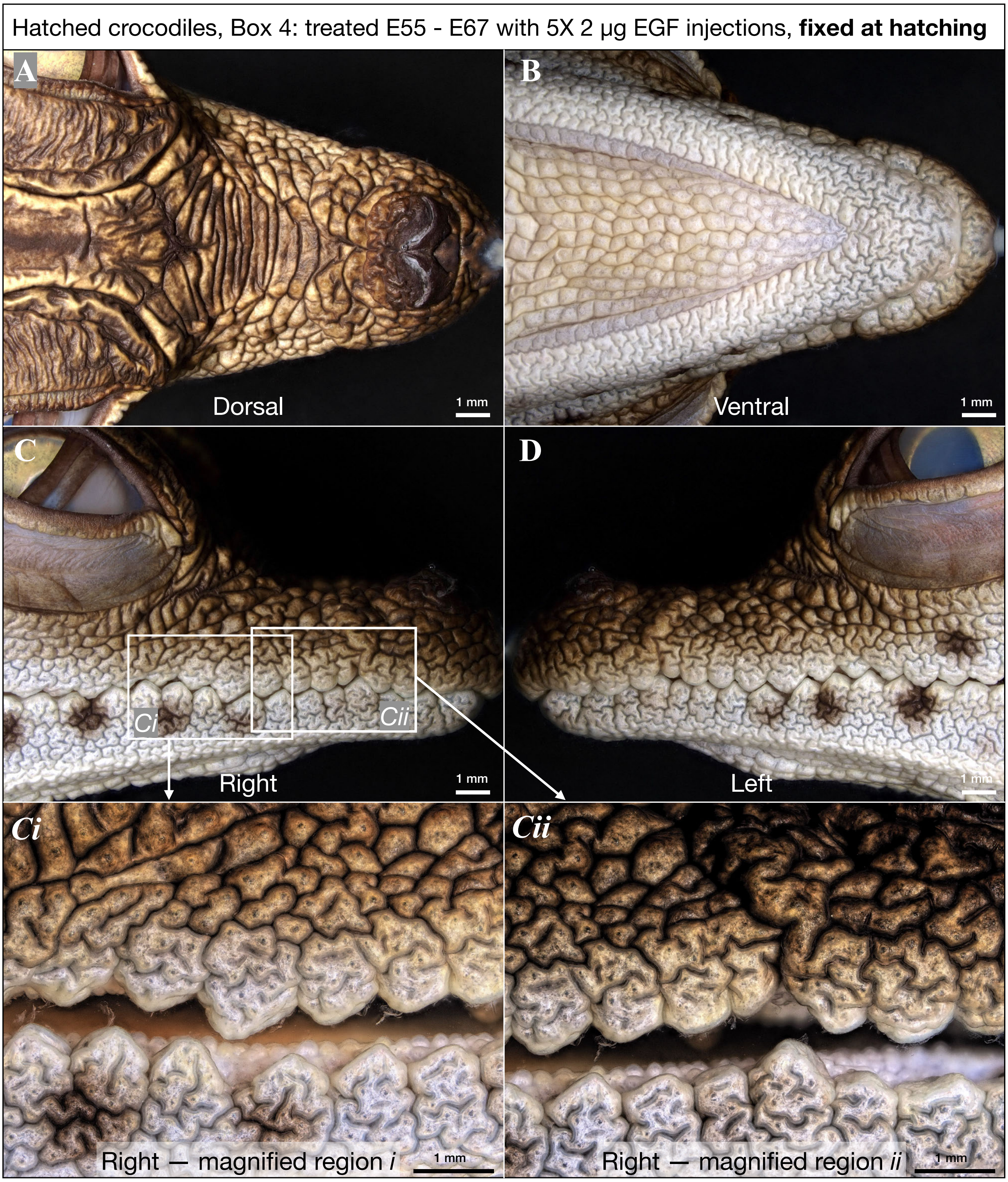

Supplementary Figure 10. Sustained *in-ovo* EGF treatment generates a labyrinthine skin surface folding pattern in hatched Nile crocodiles. An individual crocodile specimen survived after a longer treatment window including 5X injections of 2 µg EGF, from E55 to E67, prior to fixation at hatching. This individual showed extensive labyrinthine skin surface folding at the hatching stage, resulting from the high EGF dosage. This sample is also shown in Extended Data Figure 6F.

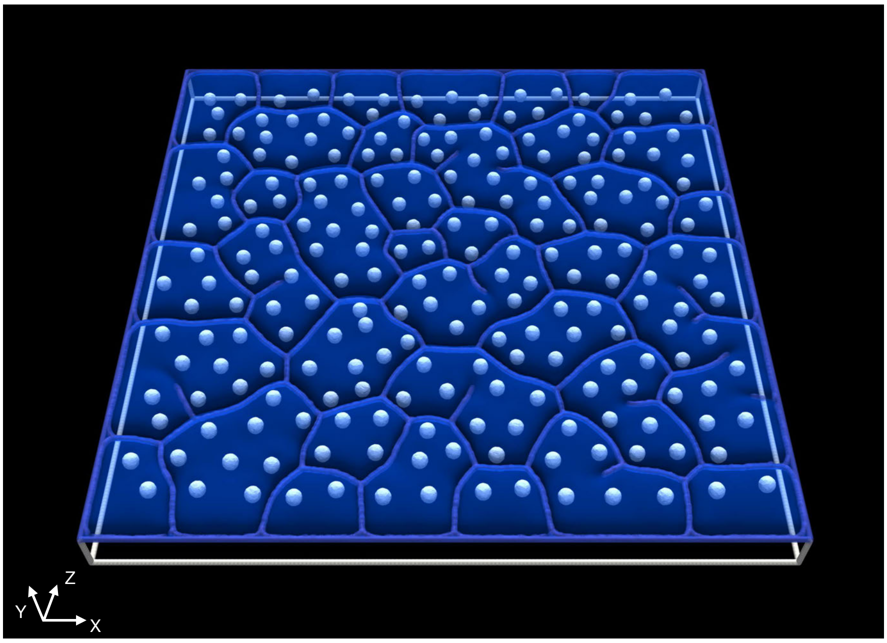

Supplementary Figure 11. Effect of integumentary sensory organs (ISOs) on head-scale patterning. Flat slab numerical simulations indicate that the compression-driven propagating folds avoid ISOs, and generate some incomplete edges, as well as a large number of 90° edge junctions at the domain boundaries. Simulation parameters are those optimised for the E64 upper jaw control sample (Supplementary Table 4). The relative thicknesses of dermis and epidermis are set to values similar to those observed in real embryos. ISOs are distributed on a noisy hexagonal lattice and their Young's modulus (relative to the dermis) and Poisson's ratio are set to 10 and 0.40, respectively.

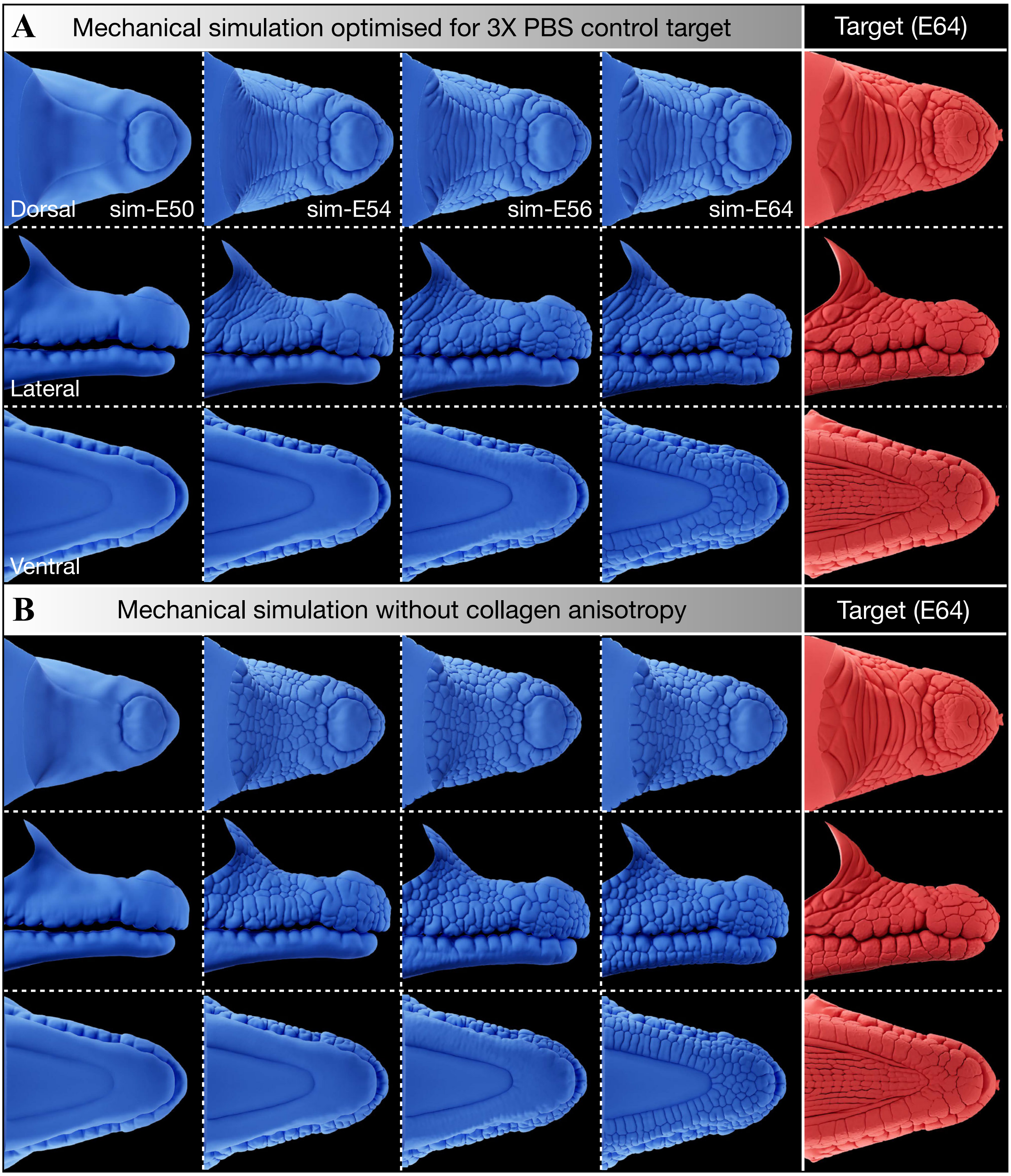

Supplementary Figure 12: Mechanical growth simulation without collagen anisotropy. (A) Our mechanical growth simulation optimised for a control target, as shown in Figure 4A, recapitulates the normal patterning of crocodile head scales. **(B)** The same simulation, in the absence of collagen architecture anisotropy, fails to successfully recapitulate the natural patterning. Specifically, we do not observe elongated scale domains on the dorsal surface of the upper jaw and instead observe smaller and more numerous scale domains on the dorsal, lateral, and ventral jaw surfaces. See also Supplementary Video 10.

2. Supplementary Notes

Supplementary Note 1: Finding the dominant orientations of collagen fibres using 3D Fourier coefficients

We assessed the orientation(s) of collagen fibres in each of 13 thousand homogeneously-distributed dermal samples (cubic patches of 50x50x50 voxels) by performing 3D Fast Fourier Transforms (FFT) on the corresponding fluorescence signal generated by Fast Green (FCF) staining (1). Note that the signal harmonics inside each patch are removed prior to performing FFT analyses to avoid boundary effects. We use the arrangement of the dominant FFT coefficients in the Fourier space to determine the orientations of fibres. For example, if one considers fibres all oriented in the same 3D orientation (see Supplementary Note 1, Fig. 1A), two dominant FFT modes (each representing a sinusoidal wave) appear as two pairs of axisymmetric coordinates in the Fourier space (red spots in Supplementary Note 1, Fig. 1B). The four dots form a square in Fourier space with its normal vector parallel to the fibre axis. The distance between the dots and the centre of the square corresponds to the fibres' spacing. If the 3D patch is populated with two or more families of fibres (*i.e.*, with different orientations), the FFT coefficients of each family form a different planar square (*i.e.*, a dominant coefficient). This approach simplifies the detection of the orientation of each fibre family as the appropriate clustering of the corresponding dominant FFT coefficients. To perform such clustering, we use a *mixture model* with probability density functions, adapted to our application.

In our mixture model, the populations are labelled by $g = 1,\ldots,G$. The probability distribution over the populations is categorical and is given by z_g with $\sum_{g=1}^{G} z_g = 1$. The probability distribution of a random variable $\boldsymbol{x}$ inside each population is given by

$$\Pr\left(\boldsymbol{x}\,|\,g\right) = f(\boldsymbol{x};\boldsymbol{\theta}_g)d\boldsymbol{x} \qquad \text{(S1-1)}$$

in which f is the probability density function and $\boldsymbol{\theta}_g$ is the set of parameters for population g. We can write the total probability as

$$\Pr(\boldsymbol{x}) = \sum_{g=1}^{G} \Pr(g)\Pr\left(\boldsymbol{x}\,|\,g\right) = \sum_{g=1}^{G} z_g f(\boldsymbol{x};\boldsymbol{\theta}_g)d\boldsymbol{x} \qquad \text{(S1-2)}$$

In our model, we assume that the Fourier coefficients associated to each fibre family (*i.e.*, each population) have the following density function

$$f(\boldsymbol{x};\boldsymbol{\theta}_g) = \frac{1}{\pi a_g b_g}\exp\left(-\frac{1}{a_g^2}\boldsymbol{x}^{\mathrm{T}}\left(\boldsymbol{n}_g\boldsymbol{n}_g^{\mathrm{T}}\right)\boldsymbol{x} - \frac{1}{b_g^2}\boldsymbol{x}^{\mathrm{T}}\left(\mathbf{I}-\boldsymbol{n}_g\boldsymbol{n}_g^{\mathrm{T}}\right)\boldsymbol{x}\right) \qquad \text{(S1-3)}$$

in which $\boldsymbol{x}$ is the coordinate of points in the Fourier space and $\boldsymbol{\theta}_g = \left(\boldsymbol{n}_g, a_g^2, b_g^2\right)^{\mathrm{T}}$ is the set of parameters for the function. Geometrically speaking, this function represents an "oblate spheroid" with a symmetric axis aligned with $\boldsymbol{n}_g$ and, its equatorial and polar radii proportional to b and a, respectively (Supplementary Note 1, Fig. 1C).

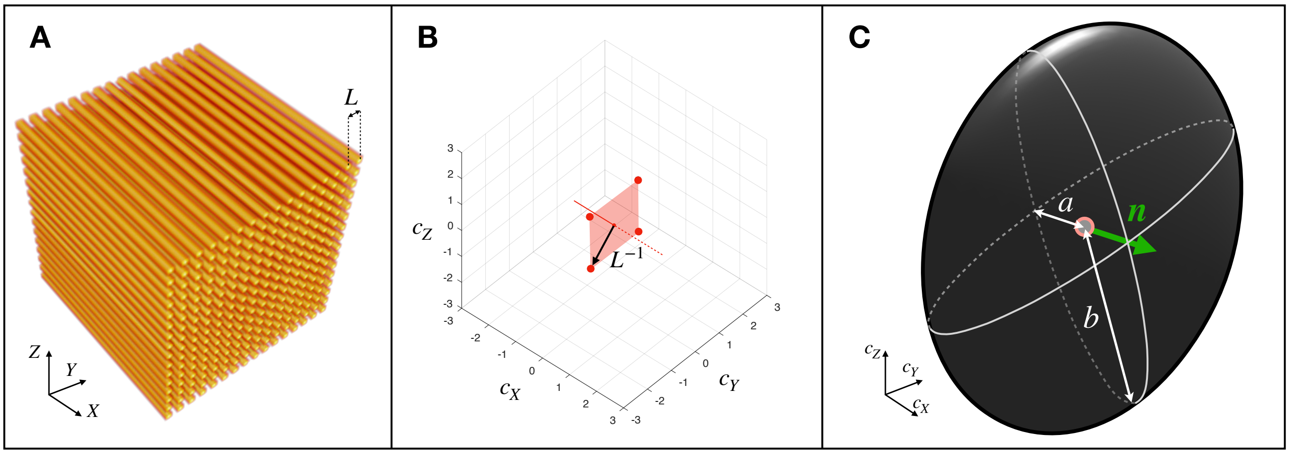

Supplementary Note 1, Figure 1 - Probability density function of a single family of fibres. (A) 3D image of an ideal single family of fibres. Fibres are elongated in the X axis and their centres are evenly distributed in the Y-Z plane with spacing (wave-length) L. **(B)** The FFT coefficients of the 3D image in (A) are visualised in the Fourier space as pairs of axisymmetric coordinates (red spots). The X,Y and Z coordinates in this plot corresponds to the wave-number (frequency) of the signal in each direction. The size of the red spots in a pair is proportional to the amplitude of the corresponding FFT mode, hence, to the 'dominance' of the corresponding wave number. In this example only four circles appear, meaning that all other coefficients vanish. The normal of the plane generated from these four points is aligned with the fibre family orientation and the distance of the spots from the centre of the square is equal to the inverse of L. **(C)** An iso-probability surface, computed from $f(\boldsymbol{x};\boldsymbol{\theta}_g)$, for a typical fibre family is visualised in Fourier space. The surface is an oblate spheroid with its symmetric axis aligned with $\boldsymbol{n}$ (also the direction of the fibre family), and its equatorial and polar radii proportional to b and a, respectively.

Furthermore, we consider the amplitudes of the Fourier coefficients as weights, w, for $\boldsymbol{x}$. In this case, the weighted probability reads

$$\Pr\left(\boldsymbol{x} \mid w\right) = \left(\sum_{g=1}^{G} z_g \Pr\left(\boldsymbol{x} \mid g\right)\right)^{w} = \left(\sum_{g=1}^{G} z_g f(\boldsymbol{x};\boldsymbol{\theta}_g) d\boldsymbol{x}\right)^{w} \tag{S1-4}$$

The likelihood of all Fourier coefficients, is computed as

$$\mathscr{L}\left(\boldsymbol{\theta}_1, \ldots, \boldsymbol{\theta}_G, z_1, \ldots, z_G\right) = \Pr\left(\boldsymbol{x}_1, \ldots, \boldsymbol{x}_N \mid w\right) = \prod_{n=1}^{N}\left(\sum_{g=1}^{G} z_g f(\boldsymbol{x}_n;\boldsymbol{\theta}_g) d\boldsymbol{x}\right)^{w_n} \tag{S1-5}$$

We are seeking for $\boldsymbol{\theta}_g$ and z_g which maximise the likelihood function $\mathscr{L}$ with the following constraints

$$\sum_{g=1}^{G} z_g = 1, \quad \boldsymbol{n}_g^{\mathrm{T}} \boldsymbol{n}_g = 1 \tag{S1-6}$$

Therefore, we can write the Lagrangian as

$$S = \log \mathscr{L} + \mu_0 \left(\sum_{g=1}^{G} z_g - 1 \right) + \sum_{g=1}^{G} \mu_g \left(\boldsymbol{n}_g^{\mathrm{T}} \boldsymbol{n}_g - 1 \right) \tag{S1-7}$$

In which μ_0 and μ_g are the Lagrange multipliers. The optimal values should satisfy $\partial_{z_g} S = 0$ and $\partial_{\boldsymbol{\theta}_g} S = 0$, which eventually results in the following set of nonlinear equations

$$z_g = \frac{1}{\sum_{n=1} w_n} \sum_n \gamma_g \left(\boldsymbol{x}_n \right) \tag{S1-8}$$

$$a_g^2 = \frac{2 \sum_n \gamma_g \left(\boldsymbol{x}_n \right) \left(\boldsymbol{x}_n^{\mathrm{T}} \left(\boldsymbol{n}_g \boldsymbol{n}_g^{\mathrm{T}} \right) \boldsymbol{x}_n \right)}{\sum_n \gamma_g \left(\boldsymbol{x}_n \right)} \tag{S1-9}$$

$$b_g^2 = \frac{2 \sum_n \gamma_g \left(\boldsymbol{x}_n \right) \left(\boldsymbol{x}_n^{\mathrm{T}} \boldsymbol{x}_n - \boldsymbol{x}_n^{\mathrm{T}} \left(\boldsymbol{n}_g \boldsymbol{n}_g^{\mathrm{T}} \right) \boldsymbol{x}_n \right)}{\sum_n \gamma_g \left(\boldsymbol{x}_n \right)} \tag{S1-10}$$

$$\mu_g \boldsymbol{n}_g = \sum_{n=1}^{N} \gamma_g \left(\boldsymbol{x}_n \right) \left(\frac{1}{b_g^2} - \frac{1}{a_g^2} \right) \left(\boldsymbol{x}_n \boldsymbol{x}_n^{\mathrm{T}} \right) \boldsymbol{n}_g \tag{S1-11}$$

with $\gamma_g \left(\boldsymbol{x}_n \right) = w_n z_g f(\boldsymbol{x}_n; \boldsymbol{\theta}_g) / \sum_{g'=1}^{G} z_{g'} f(\boldsymbol{x}_n; \boldsymbol{\theta}_{g'})$. Equations (S1-8)-(S1-11) can be solved iteratively until convergence of z_g, b_g^2, a_g^2 and $\boldsymbol{n}_g$.

We then use a Poisson-disk sampling (2) to produce a continuous field of fibre orientation(s) across the embryonic crocodile head. To achieve this aim, we select a few thousand points on the dermis midplane and perform mixture-model clustering for the cubic patches centred at those points. Note that we consider, at each point, a maximum of two dominant families of fibre directions, both tangential to the dermis mid-plane, as observed by confocal imaging of the embryonic crocodile samples (Fig. 1D and Supplementary Figure 2). We then reorder fibre families such that, at each point, the orientation mismatches with the nearest-neighbour points are minimised using a fibre orientation mismatch energy functional (see Supplementary Note 2 below for details). After reordering, we used spectral least-squares approximation (Supplementary Note 3) to extrapolate the fibre orientation(s) on the full dermis mid-plane.

Supplementary Note 2: Minimisation of fibre energy functional

Let's assume n_f as the number of fibre families in the system, with each family represented as a vector field $\boldsymbol{a}_f$ and the following associated two-tensor

$$\boldsymbol{A}_f = \boldsymbol{a}_f \boldsymbol{a}_f^{\mathrm{T}}, \quad f = 1,\ldots,n_f \qquad \text{(S2-1)}$$

We define the energy functional of fibres as

$$\mathscr{H} = \int_S d^2x \sum_{f=1}^{n_f} \left(\nabla A_{f,kl} \cdot \nabla A_{f,kl} \right) \qquad \text{(S2-2)}$$

in which k, l are summation indices. We consider that the surface is discretised into a triangular mesh. Therefore, we can use the discretised Laplace-Beltrami operator (see equation (S3-5) in Supplementary Note 3) and approximate the energy functional with

$$\mathscr{H} = \sum_{i,j} \sum_{f=1}^{n_f} L_{ij} \left(\boldsymbol{a}_{f,i} \cdot \boldsymbol{a}_{f,j} \right)^2 \qquad \text{(S2-3)}$$

in which i, j are the indices of the edge nodes. Here, we propose an algorithm for minimising $\mathscr{H}$ by optimising the order of fibres at each node. The algorithm starts by randomly selecting a node called the 'seed node'. Then, for each node connected to the seed node, we permute the order of fibres and compute its energy functional according to equation (S2-3). Looking at all possible permutations (*i.e.*, equal to $n_f!$), we keep the one which results is the lowest energy. When all connected nodes are checked, we proceed to the next iteration and repeat the same procedure, but this time for the nodes connected to the patch of nodes which has been already checked. By iterating this process, we ultimately check all points. Since we only change the fibre order of a single node by looking at the energy of its associated edges, there is a possibility that the selected order does not result in the global minimal energy. Therefore, we repeat the whole process multiple times from new seed points until convergence. For example, in the case of $n_f = 2$ (*i.e.*, two dominant orientations, as consider in our analyses), we converge to the minimal energy after about four repetitions.

Supplementary Note 3: Spectral least-square approximation

The two dominant collagen fibre orientations, both tangential to the dermis mid-plane, were assessed using spectral least-squares approximation. We assume a triangular mesh with node coordinates defined as

$$\boldsymbol{v} = \{\boldsymbol{v}_1, \ldots, \boldsymbol{v}_N\} \tag{S3-1}$$

for which, a scalar field is only known at a subset of nodes

$$f\left(\boldsymbol{x} = \boldsymbol{v}_i\right) = f_i, \qquad \boldsymbol{v}_i \in \boldsymbol{v}' : \boldsymbol{v}' \subset \boldsymbol{v} \tag{S3-2}$$

Using this information, we would like to estimate a scalar function $\tilde{f}(\boldsymbol{x})$ defined at every node of the mesh. For that purpose, we first compute the spatial modes of the 2-manifold, *i.e.*, the eigenfunctions of the Laplace-Beltrami operator computed from

$$\Delta\psi(\boldsymbol{x}) = -\lambda\psi(\boldsymbol{x}) \tag{S3-3}$$

in which Δ is the Laplace-Beltrami operator, λ are the eigenvalues and $\psi(\boldsymbol{x})$ are the eigenfunctions (spatial modes). Using a linear shape function defined on the triangular elements, the discretised version of (S3-3) reads :

$$L\psi = -\lambda M\psi \tag{S3-4}$$

where L and M denote, respectively, the discretised Laplace-Beltrami operator and the mass matrices computed as

$$L_{ij} = \begin{cases} \frac{\cot\alpha_{ij} + \cot\beta_{ij}}{2} & (i,j) \text{ edge} \\ -\sum_{k\in N(j)} L_{i,k} & i = j \\ 0 & \text{otherwise} \end{cases} \tag{S3-5}$$

and

$$M_{ij} = \begin{cases} \frac{t_1 + t_2}{12} & (i,j) \text{ edge} \\ \frac{1}{6}\sum_{k\in N(j)} t_k & i = j \\ 0 & \text{otherwise} \end{cases} \tag{S3-6}$$

in which, α_{ij} and β_{ij} are the two angles opposite to each edge (i,j), t_k is the area of each triangle *k* sharing node i, and $t_{1,2}$ are the areas of the two triangles sharing the edge (i,j). For a mesh with N nodes, we have N eigenfunctions and eigenvalues such that

$$0 = \lambda_1 \leq \ldots \leq \lambda_N \tag{S3-7}$$

We know that any arbitrary scalar function defined on a mesh can be decomposed into spatial modes. Thus $\tilde{f}(\boldsymbol{x})$ can be written as :

$$\tilde{f}(\boldsymbol{x}) = \sum_{i=1}^{N} q_i \psi_i(\boldsymbol{x}) \tag{S3-8}$$

whereas q_i is a scalar representing the amplitude of ith spatial mode. The absolute value of each amplitude indicates the contribution of the corresponding spatial mode in shaping the scalar function.

If we know the values of the function f at m positions, we can define the least-square error as

$$\mathscr{E} = \sum_{j=1}^{m} \left(\tilde{f}(\boldsymbol{x}_j) - f(\boldsymbol{x}_j) \right)^2 \tag{S3-9}$$

Substituting (S3-8) into (S3-9) yields

$$\mathscr{E} = \sum_{j=1}^{m} \left(\sum_{i=1}^{N} q_i \psi_i \left(\boldsymbol{x}_j \right) - f(\boldsymbol{x}_j) \right)^2 \tag{S3-10}$$

We know that the minimum error occurs when

$$\frac{\partial \mathscr{E}}{\partial q_k} = 0 \tag{S3-11}$$

Substituting (S3-10) into (S3-11) results into

$$\sum_{i=1}^{N} q_i \psi_i \left(\boldsymbol{x}_j \right) = f(\boldsymbol{x}_j) \tag{S3-12}$$

We can rewrite (S3-12) in a matrix form as

$$\left[\psi_i \left(\boldsymbol{x}_j \right) \right]_{m \times N} \left[q_i \right]_{N \times 1} = \left[f(\boldsymbol{x}_j) \right]_{m \times 1} \tag{S3-13}$$

Finally, the vector of amplitudes is computed as

$$\left[q_i \right]_{N \times 1} = \left[\psi_i \left(\boldsymbol{x}_j \right) \right]^{\dagger}_{N \times m} \left[f(\boldsymbol{x}_j) \right]_{m \times 1} \tag{S3-14}$$

in which † indicates the pseudo-inverse of the corresponding matrix. Now, we can use (S3-8) to compute the function $\tilde{f}(\boldsymbol{x})$ at every point of the mesh.

It is worth to mention that, most of the time, the number of spatial modes selected for computing $\tilde{f}(\boldsymbol{x})$ is much smaller than N, *i.e.*, the total number of nodes. If we select the first n modes such that $n < N$, the resulting function does not contain short-wavelengths. Therefore, n is a parameter that controls the smoothness of $\tilde{f}(\boldsymbol{x})$. Here, we choose n up to 200.

Supplementary Note 4: Finite-strain theory and anisotropic neo-Hookean material model.

We define the bulk of the material as the collection of points forming a specific configuration in 3D space. The motion of these points fully describes its deformation. We select an initial configuration as the reference configuration (*i.e.*, that did not experience any deformation) whose spatial coordinates form an invariant vector variable $\boldsymbol{X}$. To describe the current (deformed) configuration of the material points during numerical growth simulations, we define a vector variable $\boldsymbol{x}$ which stores the current spatial coordinates of the points :

$$\boldsymbol{x} = \boldsymbol{x}\,(\boldsymbol{X}, t) \tag{S4-1}$$

Clearly, at reference configuration ($t = 0$) we can write

$$\boldsymbol{x}\,(\boldsymbol{X}, 0) = \boldsymbol{X} \tag{S4-2}$$

The first and second time derivatives of the points $\boldsymbol{x}$ are, respectively their velocities and accelerations:

$$\boldsymbol{v} = \frac{d\boldsymbol{x}}{dt}, \quad \boldsymbol{a} = \frac{d^2\boldsymbol{x}}{dt^2}. \tag{S4-3}$$

Then, we define the deformation gradient, $\boldsymbol{F}$, as a second-order tensor which relates the reference configuration to the current configuration :

$$\boldsymbol{F} = \frac{d\boldsymbol{x}}{d\boldsymbol{X}} = F_{iJ}\mathbf{e}_i \otimes \mathbf{E}_J \tag{S4-4}$$

where, $\mathbf{e}_i$ and $\mathbf{E}_J$, denote the Cartesian unit vectors in the current and reference configurations, respectively, and $i, J = 1,2,3$ are the corresponding summation indices.

Neo-Hookean material models (a sub-group of *hyper-elastic* models) are appropriate for soft material (bio-)mechanics because they are robust even under large deformations(3-5). Importantly, we use a standard phenomenological model in which mechanical parameters are *effective* parameters that ignore lower scales. For example, the various layers within the epidermis are likely to exhibit different stiffnesses (Young's moduli), but their combination produces an effective 'mean' modulus for the all epidermal depth. Multiple studies(4-9) have demonstrated that mesoscopic and macroscopic properties of biological dynamical systems can be captured by such models without integrating the unmanageable profusion of variables that populate the nanoscopic and microscopic scales(10). In hyper-elastic models, the stress tensor is computed from the *strain energy density function*, which can be described by a deformation gradient together with material properties. The strain energy density function describes the potential elastic energy stored at each point of the material. These models allow for the effective incorporation of anisotropic behaviours, such as that of collagen fibres(11), which are resistant to deformation along their axis. In that case, the anisotropic strain energy can be incorporated into the whole strain energy function:

$$\Psi = \Psi_{\text{iso}} + \Psi_{\text{aniso}} \tag{S4-5}$$

whereas, Ψ_{iso}, Ψ_{aniso} and Ψ denote isotropic, anisotropic and total strain energy density functions, respectively. Similarly to(12), we use the following neo-Hookean model for the isotropic material behaviour:

$$\Psi_{\text{iso}} = \frac{\mu}{2}\left(\text{tr}\left(\boldsymbol{F}\boldsymbol{F}^{\text{T}}\right)J^{-\frac{2}{3}} - 3\right) + K\left(J - \ln J - 1\right), \tag{S4-6}$$

where $J = \det \boldsymbol{F}$, and μ and K are shear and bulk moduli, respectively, which are related to Young's modulus E and Poisson's ratio ν as follows:

$$\mu = \frac{E}{2(1+\nu)} \text{ and } K = \frac{E}{3(1-2\nu)}. \tag{S4-7}$$

For the anisotropic component, we modified the model of[11] as follows:

$$\Psi_{\text{aniso}} = \frac{k_1}{2k_2}\sum_{i=1}^{n_f}\left(\exp\left(k_2\left(I_i - \mathscr{A}_i^2\right)^2\right) - 1\right), \tag{S4-8}$$

where k_1 and k_2 are the fibre stiffness constants, n_f is the number of fibre families, $\mathscr{A}_i = \|\boldsymbol{\mathscr{A}}_i\|$ is the Euclidian norm of the fibre vectors, and I_i is the fibre strain invariant defined as

$$I_i = \boldsymbol{\mathscr{A}}_i \cdot \left(\boldsymbol{F}^{\text{T}}\boldsymbol{F}\boldsymbol{\mathscr{A}}_i\right). \tag{S4-9}$$

Note that in the original model[11], fibre vectors are unit vectors, which implies $\mathscr{A}_i = 1$.

In the framework of hyper-elastic materials, we compute the Cauchy stress $\boldsymbol{\sigma}$ directly from the strain energy density function:

$$\boldsymbol{\sigma} = \frac{1}{J}\frac{\partial \Psi}{\partial \boldsymbol{F}}\boldsymbol{F}^{\text{T}} \tag{S4-10}$$

Substituting (S4-5) into (S4-10) results in following stress function

$$\boldsymbol{\sigma} = \boldsymbol{\sigma}_{\text{iso}} + \boldsymbol{\sigma}_{\text{aniso}} \tag{S4-11}$$

with

$$\boldsymbol{\sigma}_{\text{iso}} = \mu J^{-5/3}\text{dev}\left(\boldsymbol{F}\boldsymbol{F}^{\text{T}}\right) + K\left(1 - \frac{1}{J}\right)\mathbf{I} \tag{S4-12}$$

$$\boldsymbol{\sigma}_{\text{aniso}} = 2k_1 J^{-1}\sum_i \left(I_i - \mathscr{A}_i^2\right)\exp\left(k_2\left(I_i - \mathscr{A}_i^2\right)^2\right)\boldsymbol{F}\boldsymbol{\mathscr{A}}_i \otimes \boldsymbol{F}\boldsymbol{\mathscr{A}}_i \tag{S4-13}$$

If fibres are under compression, their strain invariant becomes smaller than one, *i.e.* $I_i < 1$. In that case, the anisotropic component $\boldsymbol{\sigma}_{\text{aniso}}$ is removed to avoid unrealistic calculations.

According to the balance of internal forces, multiplying all stress components by a constant value does not change the steady-state solution. Hence, only the ratios of stiffnesses (and not their absolute values) among interacting tissue layers and fibres are relevant.

Supplementary Note 5: Computing forces in the FEM framework

The finite-element method (FEM) is a standard approach for solving problems in solid mechanics. Using this technique, we discretise the reference configuration into small elements. Here we consider tetrahedral elements which are the simplest volumetric shapes constructed with 4 nodes. This choice enables us to perform a linear interpolation of displacement inside each element such that the deformation gradient tensor of each element is computed as :

$$\boldsymbol{F} = [\boldsymbol{x}_1 - \boldsymbol{x}_4, \quad \boldsymbol{x}_2 - \boldsymbol{x}_4, \quad \boldsymbol{x}_3 - \boldsymbol{x}_4] \left[\boldsymbol{X}_1 - \boldsymbol{X}_4, \quad \boldsymbol{X}_2 - \boldsymbol{X}_4, \quad \boldsymbol{X}_3 - \boldsymbol{X}_4\right]^{-1} \qquad \text{(S5-1)}$$

in which $\boldsymbol{x}_n$ and $\boldsymbol{X}_n$ with $n = 1,2,3,4$ denote the current and reference position vectors of the tetrahedral nodes, respectively. Matrices appearing in equation (S5-1) are 3x3 and the second matrix in the right hand side is invertible if the tetrahedron volume at the reference configuration is non zero. The deformation gradient computed by equation (S5-1) is constant inside each element. In this specific case, it has been shown (13) that the discretisation of FEM is exactly equivalent to the node-centred finite-volume approach. Here, we follow the later notations since it is geometrically more intuitive. After computing the deformation gradient, we compute the Cauchy stress tensor for each element using equations (S4-12) and (S4-13) in the Supplementary Note 4. This stress tensor converts to four forces when multiplied by the area vectors of the tetrahedron triangular facets. The facial forces are then equally divided between the three nodes constructing each triangle. Therefore, nodal force can be computed as

$$\boldsymbol{f}_{n,\text{elastic}} = \frac{1}{3} \sum_{e} \boldsymbol{\sigma}_e \cdot \left(\boldsymbol{A}_1 + \boldsymbol{A}_2 + \boldsymbol{A}_3\right) \qquad \text{(S5-2)}$$

in which $\boldsymbol{f}_n$ is the total force applied on the nth node, e denotes the tetrahedra sharing the nth node, and $\boldsymbol{A}_{1,2,3}$ are the three area vectors of the triangles both belonging to element e and containing node n (see Supplementary Note 5, Fig. 2A).

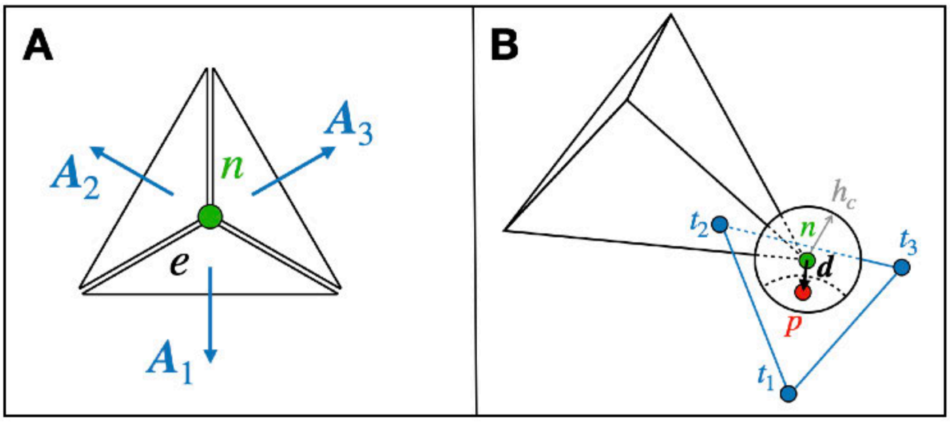

Supplementary Note 5, Figure 2 - Tetrahedral element. (A) Node n (green spot) in tetrahedron e is common among three surface triangles. The corresponding area vectors $\boldsymbol{A}_{1,2,3}$ are used to calculate the facial forces. **(B)** When a node n from a tetrahedron is in contact with a face (here, the triangle $\boldsymbol{t}_{1,2,3}$) of another tetrahedron, contact forces must be computed. First, the distance vector $\boldsymbol{d}$ that connects node n to its projection, $\boldsymbol{p}$, on the triangle $\boldsymbol{t}_{1,2,3}$ is computed. Then, the contact force is computed based on the depth of penetration of an imaginary sphere, of radius h_c (one third of the mean size of all tetrahedra) into the triangle.

Knowing the nodal forces, we use Newton's second law of motion and perform explicit time integration. Similar to reference (12), we use a damped Euler forward scheme:

$$m_n \frac{\boldsymbol{v}_n^{(t+\delta t)} - \boldsymbol{v}_n^{(t)}}{\delta t} = \boldsymbol{f}_n^{(t)} - \gamma \boldsymbol{v}_n^{(t)} \qquad \text{(S5-3)}$$

in which, δt is the time-step, $\boldsymbol{v}_n$ is the velocity of the nth node, $\boldsymbol{f}_n$ is the total force applied on the node, $\gamma_n = \dfrac{m_n}{20}$ is the damping coefficient and m_n is the nodal mass computed from

$$m_n = \frac{1}{4} \sum_e \rho V_e \qquad \text{(S5-4)}$$

In equation (S5-4), V_e denotes the volume of the tetrahedra sharing the nth node, and ρ is the density. Here, we consider homogenous and constant density $\rho = 1$ for all tissues because our results are not affected by the inertia of the nodes. It is worth to mention that, in the context of the finite-element method, equation (S5-4) is precisely the so-called 'lumped-mass matrix' in which the diagonal entries are equal to m_n and off-diagonal entries are zero.

In order to complete the time integration, we update the positions of nodes using

$$\frac{\boldsymbol{x}_n^{(t+\delta t)} - \boldsymbol{x}_n^{(t)}}{\delta t} = \boldsymbol{v}_n^{(t+\delta t)} \qquad \text{(S5-5)}$$

The total force appearing in (S5-3) reads

$$\boldsymbol{f}_n = \boldsymbol{f}_{n,\text{elastic}} + \boldsymbol{f}_{n,\text{contact}} + \boldsymbol{f}_{n,\text{viscous}} \qquad \text{(S5-6)}$$

in which $\boldsymbol{f}_{n,\text{contact}}$ and $\boldsymbol{f}_{n,\text{viscous}}$ are contact and viscous forces presented in the next sections.

Contact forces. In case of contact, we use a penalty force approach in which any penetration of a node into the surface of any other tetrahedron is penalised by a force proportional to the penetration depth (see Supplementary Note 5, Fig. 2). More precisely, at each time step, we look for Hertzian contacts defined by an elastic sphere (representing a tetrahedron node) indenting an elastic surface (representing a face of another tetrahedron), producing the following penalty force

$$\boldsymbol{f}_{\text{penalty}} = \kappa E_* h_c^{1/2} \left(\max(h_c - \|\boldsymbol{d}\|, 0)\right)^{3/2} \frac{\boldsymbol{d}}{\|\boldsymbol{d}\|} \qquad \text{(S5-7)}$$

in which κ is a force factor, h_c is the radius of the imaginary sphere located at the node and $\boldsymbol{d}$ is the penetration vector (see Supplementary Note 5, Fig. 2B). E_* is the effective stiffness computed as

$$E_* = \frac{E_n E_t}{E_n\left(1 - \nu_t^2\right) + E_t\left(1 - \nu_n^2\right)}. \qquad \text{(S5-8)}$$

E_n and E_t are Young's moduli of the nodes and ν_n, ν_t are their Poisson's ratios. We then update the nodal forces for all 4 nodes involved in the contact event, *i.e.*, one node penetrating the surface triangle and the three nodes forming the surface triangle:

$$\boldsymbol{f}_{n,\text{contact}} \leftarrow \boldsymbol{f}_{n,\text{contact}} - \boldsymbol{f}_{\text{penalty}} \tag{S5-9}$$

$$\boldsymbol{f}_{t_i,\text{contact}} \leftarrow \boldsymbol{f}_{t_i,\text{contact}} + \alpha_i \boldsymbol{f}_{\text{penalty}} \tag{S5-10}$$

in which $i = 1,2,3$ is the index of the triangle nodes and $\alpha_i \geq 0$ are the barycenteric coordinates on the triangle which satisfies $\alpha_1 + \alpha_2 + \alpha_3 = 1.$

Viscous forces. Biological tissues are demonstrating viscoelastic behaviour during deformation. Moreover, the presence of viscous effects can improve the stability of the numerical model by diminishing short wave length fluctuations. Here, we use the Kelvin–Voigt viscoelastic model in which the viscous force is added to the total force. Similar to the elastic force in equation (S5-2), the viscous force for n^{th} node is

$$\boldsymbol{f}_{n,\text{viscous}} = \frac{1}{3} \sum_e \boldsymbol{\sigma}_e^{(v)} \cdot \left(\boldsymbol{A}_1 + \boldsymbol{A}_2 + \boldsymbol{A}_3\right) \tag{S5-11}$$

in which the viscous stress for each tetrahedron is computed as

$$\boldsymbol{\sigma}^{(v)} = \eta \left(\nabla \boldsymbol{v} + \nabla \boldsymbol{v}^{\mathrm{T}} \right) \tag{S5-12}$$

whereas η is the viscosity constant and the gradient of velocity for a tetrahedral element is:

$$\nabla \boldsymbol{v} = [\boldsymbol{v}_1 - \boldsymbol{v}_4, \quad \boldsymbol{v}_2 - \boldsymbol{v}_4, \quad \boldsymbol{v}_3 - \boldsymbol{v}_4] \cdot [\boldsymbol{x}_1 - \boldsymbol{x}_4, \quad \boldsymbol{x}_2 - \boldsymbol{x}_4, \quad \boldsymbol{x}_3 - \boldsymbol{x}_4]^{-1} \tag{S5-13}$$

Supplementary Note 6: Growth model

Tissue growth can be effectively modelled in the finite-strain theory framework. For that propose, the deformation gradient tensor $\boldsymbol{F}$ is decomposed into elastic and growth components $\boldsymbol{F}_e$ and $\boldsymbol{F}_g$, respectively. Here, we perform this decomposition by computing the product of the two deformation tensors:

$$\boldsymbol{F} = \boldsymbol{F}_e \boldsymbol{F}_g, \tag{S6-1}$$

Here, $\boldsymbol{F}_g$ describes the effective growth of the tissue and can be derived from the biological experiments/observations. Since $\boldsymbol{F}_g$ contributes to the isotropic stress only through $\boldsymbol{F}_e$, in accordance with Tallinen et al. 2013 (12) the isotropic stress reads

$$\boldsymbol{\sigma}_{\text{iso}} = \mu J_e^{-5/3} \text{dev}\left(\boldsymbol{F}_e \boldsymbol{F}_e^{\text{T}}\right) + K\left(1 - \frac{1}{J_e}\right)\mathbf{I}, \tag{S6-2}$$

where $J_e = \det \boldsymbol{F}_e$ and $\boldsymbol{F}_e = \boldsymbol{F}\boldsymbol{F}_g^{-1}$.

Fibres and substrate are two different components that could, in principle, exhibit different growth functions. Here, to allow fibres to exert anisotropic properties to the tissue, we consider that their relative growth (in comparison to the substrate material) is zero. Hence, equation (S4-13) can be used directly.

In our model, we define the growth of the tissue in the form of a rate equation

$$\dot{\boldsymbol{F}}_g = \boldsymbol{f}(t) \tag{S6-3}$$

in which $\boldsymbol{f}(t)$ is the growth rate function. By integrating the growth rate in time, the growth deformation gradient is obtained. Defining the growth rate function enables us to model the effect of the EGF drug more precisely. The time derivative of the growth deformation gradient (the rate of growth), is equal to the gradient of the growth velocities in the reference configuration, *i.e.*, $\dot{\boldsymbol{F}}_g = \nabla_{\boldsymbol{X}} \boldsymbol{V}_g$. Therefore, by defining $\boldsymbol{f}(t)$, we impose that the velocity field of the growth process in the tissue has a known gradient. We can define $\boldsymbol{f}(t)$ such that it is diagonalizable and its eigenvectors form an arbitrary time-invariant basis such that

$$\boldsymbol{f}(t) = \sum_{i=1}^{3} \lambda_i(t) \boldsymbol{E}_i \otimes \boldsymbol{E}_i \tag{S6-4}$$

in which $\boldsymbol{E}_i$ and λ_i are the eigenvalues and eigenvectors of $\boldsymbol{f}(t)$, respectively. Here, we are interested to decompose growth into normal and tangent components (with respect to the epidermal surface). In this case, equation (S6-4) simplifies to

$$\boldsymbol{f}(t) = \lambda_N(t) \boldsymbol{N} \otimes \boldsymbol{N} + \lambda_T(t)(\mathbf{I} - \boldsymbol{N} \otimes \boldsymbol{N}) \tag{S6-5}$$

in which, $\boldsymbol{N}$ is the normal vector in the reference configuration, and $\mathbf{I}$ is the identity matrix. Furthermore, λ_N and λ_T, as the eigenvalues of $\boldsymbol{f}(t)$, determine the rate of growth in the normal and tangential directions, respectively. If we integrate equation (S6-5) in time, we have

$$\boldsymbol{F}_g(t) - \boldsymbol{F}_g(0) = \boldsymbol{N} \otimes \boldsymbol{N} \int_0^t \lambda_N(\tau)\, d\tau + (\mathbf{I} - \boldsymbol{N} \otimes \boldsymbol{N}) \int_0^t \lambda_T(\tau)\, d\tau. \tag{S6-6}$$

The amount of growth at t=0 is zero, which implies that $\boldsymbol{F}_g(0) = \mathbf{I}$. Therefore, we can write

$$\boldsymbol{F}_g(t) = \mathbf{I} + \boldsymbol{N} \otimes \boldsymbol{N} \int_0^t \lambda_N(\tau)\, d\tau + (\mathbf{I} - \boldsymbol{N} \otimes \boldsymbol{N}) \int_0^t \lambda_T(\tau)\, d\tau. \quad \text{(S6-7)}$$

Here, we consider that the normal and tangential growth rate functions are decaying exponentially in time. However, for the cases of transient increased growth due to EGF treatment, we consider an additional non-zero constant rate function in a defined time window for $t \in [t_1, t_2]$, such that the growth rate function becomes

$$\lambda_{N/T}(t) = \lambda_{N/T}^{\text{ctrl}} \exp\left(-\beta t\right) + \lambda_{N/T}^{\text{EGF}} \left(H(t - t_1) - H(t - t_2)\right) \quad \text{(S6-8)}$$

in which, $\lambda_{N/T}^{\text{ctrl}}$ and $\lambda_{N/T}^{\text{EGF}}$ are the growth rates constants corresponding to the control (PBS) and EGF treatments, respectively. H is the Heaviside function and $\beta > 0$ is the rate constant. By substituting equation (S6-8) into (S6-7), the growth deformation gradient reads

$$\begin{aligned} \boldsymbol{F}_g(t) = \mathbf{I} &+ \left[\frac{\lambda_N^{\text{ctrl}}}{\beta}\left(1 - \exp\left(-\beta t\right)\right) + \lambda_N^{\text{EGF}} W_{t_1,t_2}(t)\right] \boldsymbol{N} \otimes \boldsymbol{N} \\ &+ \left[\frac{\lambda_T^{\text{ctrl}}}{\beta}\left(1 - \exp\left(-\beta t\right)\right) + \lambda_T^{\text{EGF}} W_{t_1,t_2}(t)\right] (\mathbf{I} - \boldsymbol{N} \otimes \boldsymbol{N}) \end{aligned} \quad \text{(S6-9)}$$

with $W_{t_1,t_2}(t) = (t - t_1)H(t - t_1) - (t - t_2)H(t - t_2)$. To consider the spatial variation of growth (measured as variation of cell-proliferation density, $\rho_{\text{EdU}}(\boldsymbol{x})$), we first rescale the density map into a scalar function (ranging between 0 and 1) that divides the domain into a high *versus* low cell-proliferation regions:

$$s(\boldsymbol{x}) = \frac{1}{2} + \frac{1}{2}\tanh\left(20\left[\frac{\rho_{\text{EdU}}(\boldsymbol{x}) - \rho_{\min}}{\rho_{\max} - \rho_{\min}} - \frac{1}{2}\right]\right) \quad \text{(S6-10)}$$

in which, $\rho_{\min\,/\,\max}$ is the minimum / maximum value of $\rho_{\text{EdU}}(\boldsymbol{x})$. Using $s(\boldsymbol{x})$, we define the growth map $G(\boldsymbol{x})$ as

$$G(\boldsymbol{x}) = s(\boldsymbol{x})\left(G^+ - G^-\right) + G^- \quad \text{(S6-11)}$$

in which G^+ and G^- are the growth constants corresponding to high and low cell-proliferation regions, respectively. By combining equations (S6-9) and (S6-11), the spatio-temporal function of the growth deformation gradient reads as

$$\begin{aligned} \boldsymbol{F}_g(\boldsymbol{x}, t) = \mathbf{I} &+ \left[G_N(\boldsymbol{x})\left(1 - \exp\left(-\beta_N(\boldsymbol{x})t\right)\right) + g_N(\boldsymbol{x})\lambda_N^{\text{EGF}} W_{t_1,t_2}(t)\right] \boldsymbol{N} \otimes \boldsymbol{N} \\ &+ \left[G_T(\boldsymbol{x})\left(1 - \exp\left(-\beta_T(\boldsymbol{x})t\right)\right) + g_T(\boldsymbol{x})\lambda_T^{\text{EGF}} W_{t_1,t_2}(t)\right] (\mathbf{I} - \boldsymbol{N} \otimes \boldsymbol{N}) \end{aligned} \quad \text{(S6-12)}$$

in which $G_{N/T}(\boldsymbol{x})$ is the growth map for the normal / tangential directions, and $g_{N/T}(\boldsymbol{x}) = \exp\left(7\left(G_{N/T}(\boldsymbol{x})/G^{+}_{N/T} - 1\right)\right)$ determines the spatial dependency of drug-induced growth. Using $g_{N/T}(\boldsymbol{x})$ in the simulations results in formation of elongated dorsal scales more similar to the observed treated samples. Furthermore, $\beta_{N/T}(\boldsymbol{x})$ is defined as

$$\beta_{N/T}(\mathbf{x}) = \frac{\beta_{\mathrm{ref}} G^{+}}{G_{N/T}(\mathbf{x})} \quad \text{(S6-13)}$$

in which $\beta_{\mathrm{ref}} = 0.3$ is the reference rate constant. Defining $\beta_{N/T}(\boldsymbol{x})$ as equation (S6-13) ensures that every points in a given skin layer (epidermis or dermis) grows initially with the same rate irrespective to its steady-state growth value defined in $G(\boldsymbol{x})$. This feature results in a better agreement between the simulated folding dynamics and the developmental stages observed in LSFM imaging.

Supplementary Note 7: Bayesian optimisation

Bayesian Optimisation is a machine-learning global minimisation algorithm suitable for solving problems with objective functions that are continuous, expensive to evaluate, have less than 20 dimensions and whose feasible parameter set is a hyper-rectangle or a d-dimensional simplex (14).

Formally, we write the problem as $\min_{\boldsymbol{x}} f(\boldsymbol{x})$, in which $f(\boldsymbol{x})$ is the objective function and $\boldsymbol{x} \in \mathbb{R}^d$ is the optimisable variable vector. We assume that the objective function belongs to a random distribution called a *Gaussian process* defined as

$$f(\boldsymbol{x}) \sim P(f) = \mathcal{GP}\left(m(\boldsymbol{x}), k(\boldsymbol{x}, \boldsymbol{x}')\right) \tag{S7-1}$$

in which $m(\boldsymbol{x})$ and $k(\boldsymbol{x}, \boldsymbol{x}')$ are the mean and covariance functions, respectively. The covariance function $k(\boldsymbol{x}, \boldsymbol{x}')$ we use here is:

$$k(\boldsymbol{x}, \boldsymbol{x}') = \sigma_f^2 \left(1 + \sqrt{5}h + \frac{5}{3}h^2\right) \exp\left(-\sqrt{5}h\right) \tag{S7-2}$$

in which

$$h = \sqrt{(\boldsymbol{x} - \boldsymbol{x}')\boldsymbol{\sigma}^{-2}(\boldsymbol{x} - \boldsymbol{x}')^{\mathrm{T}}}, \quad \boldsymbol{\sigma} = \begin{pmatrix} \sigma_1 & 0 & 0 \\ 0 & \ddots & 0 \\ 0 & 0 & \sigma_d \end{pmatrix} \tag{S7-3}$$

The scalar σ_f and the diagonal matrix $\boldsymbol{\sigma}$ are called *hyper-parameters* (that allow handling different scales in parameter space) whose values are adapted within the optimisation loop.

The Gaussian process can be described as a generalisation of the multivariate Normal distribution to infinite dimensions. Therefore, similar to the Normal distribution, the Gaussian process is used to sample random functions. Now, let's assume that we already observed N data points, meaning that their objective functions and associated variable vectors are known as

$$\mathcal{F} = \begin{pmatrix} f(\boldsymbol{x}_1) \\ \vdots \\ f(\boldsymbol{x}_N) \end{pmatrix}, \qquad \boldsymbol{X} = \begin{pmatrix} \boldsymbol{x}_1 \\ \vdots \\ \boldsymbol{x}_N \end{pmatrix}. \tag{S7-4}$$

Therefore, for the next observation, we can compute the conditional probability of the objective function as

$$P\left(f_{N+1} \,|\, \mathcal{F}\right) = \frac{P\left(\mathcal{F}, f_{N+1}\right)}{P\left(\mathcal{F}\right)} = \frac{P\left(\mathcal{F}, f_{N+1}\right)}{\int P\left(\mathcal{F}, f\right) df} \tag{S7-5}$$

in which $f_{N+1} = f\left(\boldsymbol{x}_{N+1}\right)$. By definition, any finite set of data chosen from a Gaussian process exhibit a Normal joint distribution. Since $\mathcal{F}$ and f_{N+1} are sampled from the same Gaussian process, the probability $P\left(\mathcal{F}, f_{N+1}\right)$ is a Normal joint distribution which reads

$$P\left(\mathscr{F}, f_{N+1}\right) = \mathcal{N}\left(\begin{pmatrix} \boldsymbol{M} \\ m_{N+1} \end{pmatrix}, \begin{pmatrix} \boldsymbol{K} & \boldsymbol{K}_{N+1}^{\mathrm{T}} \\ \boldsymbol{K}_{N+1} & k_{N+1} \end{pmatrix}\right) \tag{S7-6}$$

whereas, $m_{N+1} = m\left(\boldsymbol{x}_{N+1}\right)$, $k_{N+1} = k\left(\boldsymbol{x}_{N+1}, \boldsymbol{x}_{N+1}\right)$, $\boldsymbol{M} = \left(m(\boldsymbol{x}_1), \ldots, m(\boldsymbol{x}_N)\right)^{\mathrm{T}}$and $\boldsymbol{K}$ and $\boldsymbol{K}_{N+1}$ are covariance matrices defined as

$$\boldsymbol{K} = \begin{pmatrix} k(\boldsymbol{x}_1, \boldsymbol{x}_1) & \ldots & k(\boldsymbol{x}_1, \boldsymbol{x}_N) \\ \vdots & \ddots & \vdots \\ k(\boldsymbol{x}_N, \boldsymbol{x}_1) & \ldots & k(\boldsymbol{x}_N, \boldsymbol{x}_N) \end{pmatrix}, \qquad \boldsymbol{K}_{N+1} = \begin{pmatrix} k(\boldsymbol{x}_1, \boldsymbol{x}_{N+1}) \\ \vdots \\ k(\boldsymbol{x}_N, \boldsymbol{x}_{N+1}) \end{pmatrix} \tag{S7-7}$$

Substituting equation (S7-6) into equation (S7-5) and computing the integral of the latter, it can be shown that $P\left(f_{N+1} \mid \mathscr{F}\right)$ is itself a Normal distribution defined as

$$P\left(f_{N+1} \mid \mathscr{F}\right) = \mathcal{N}\left(m_{\text{post}}\left(\boldsymbol{x}_{N+1}\right), k_{\text{post}}\left(\boldsymbol{x}_{N+1}, \boldsymbol{x}'_{N+1}\right)\right) \tag{S7-8}$$

in which

$$m_{\text{post}}\left(\boldsymbol{x}_{N+1}\right) = m\left(\boldsymbol{x}_{N+1}\right) + \boldsymbol{K}_{N+1}^{\mathrm{T}} \boldsymbol{K}^{-1}\left(\mathscr{F} - \boldsymbol{M}\right), \tag{S7-9}$$

and

$$k_{\text{post}}\left(\boldsymbol{x}_{N+1}, \boldsymbol{x}'_{N+1}\right) = k\left(\boldsymbol{x}_{N+1}, \boldsymbol{x}'_{N+1}\right) - \boldsymbol{K}_{N+1}^{\mathrm{T}} \boldsymbol{K}^{-1} \boldsymbol{K}_{N+1}. \tag{S7-10}$$

This allows us to update equation (S7-1) as

$$f(\boldsymbol{x}) \sim \mathscr{GP}\left(m_{\text{post}}(\boldsymbol{x}), k_{\text{post}}(\boldsymbol{x}, \boldsymbol{x}')\right). \tag{S7-11}$$

In comparison to the original Gaussian process in equation (S7-1), equation (S7-11) is a more precise approximation of $f(\boldsymbol{x})$ because its covariance function is always smaller, which implies that the uncertainty of the observations become lower.

To guess the next sampling point we consider an *improvement function* defined as :

$$I\left(f(\boldsymbol{x})\right) = \begin{cases} f_{\min} - f(\boldsymbol{x}) & f(\boldsymbol{x}) < f_{\min} \\ 0 & f(\boldsymbol{x}) \geq f_{\min} \end{cases} \tag{S7-12}$$

in which $f_{\min}$ is the minimum value of the objective function evaluated so far. This improvement function indicates the amount by which the objective function is expected to be reduced with respect to $f_{\min}$. Note that, for values of the objective function that exceeds $f_{\min}$, their contributions to the integral at any point $(\boldsymbol{x})$ in parameter space are set to zero when computing the improvement function expectation. The latter takes the following closed form (15):

$$
\begin{aligned}
\mathbb{E}\left[I\left(f\right)\right] &= \int_{-\infty}^{\infty} I\left(f\right) \mathscr{G}\mathscr{P}\left(m_{\text{post}}(\boldsymbol{x}), k_{\text{post}}(\boldsymbol{x},\boldsymbol{x})\right) df \\
&= \left(f_{\min} - m_{\text{post}}(\boldsymbol{x})\right) \Phi\left(\frac{f_{\min} - m_{\text{post}}(\boldsymbol{x})}{k_{\text{post}}(\boldsymbol{x},\boldsymbol{x})}\right) \quad + \\
&\quad k_{\text{post}}(\boldsymbol{x},\boldsymbol{x})\, \phi\left(\frac{f_{\min} - m_{\text{post}}(\boldsymbol{x})}{k_{\text{post}}(\boldsymbol{x},\boldsymbol{x})}\right)
\end{aligned}
\tag{S7-13}
$$

where ϕ and Φ are the probability density function and cumulative density function of the standard Normal distribution, respectively. Note that this expectation function is much cheaper to evaluate than the objective function. The point to sample in the next iteration is the one that maximises the expected improvement function in equation (S7-13). This classical minimisation problem is efficiently handled by a gradient descent algorithm.

References

1. G. Timin, M. C. Milinkovitch, High-resolution confocal and light-sheet imaging of collagen 3D network architecture in very large samples. *Iscience* **26**, (2023).
2. M. Corsini, P. Cignoni, R. Scopigno, Efficient and Flexible Sampling with Blue Noise Properties of Triangular Meshes. *Ieee T Vis Comput Gr* **18**, 914-924 (2012).
3. M. Ben Amar, F. Jia, Anisotropic growth shapes intestinal tissues during embryogenesis. *Proc Natl Acad Sci U S A* **110**, 10525-10530 (2013).
4. A. E. Shyer *et al.*, Villification: How the Gut Gets Its Villi. *Science* **342**, 212-218 (2013).
5. T. Tallinen *et al.*, On the growth and form of cortical convolutions. *Nat Phys* **12**, 588-593 (2016).
6. L. Manukyan, S. A. Montandon, A. Fofonjka, S. Smirnov, M. C. Milinkovitch, A living mesoscopic cellular automaton made of skin scales. *Nature* **544**, 173-179 (2017).
7. A. Chuyen *et al.*, The Scf/Kit pathway implements self-organized epithelial patterning. *Dev Cell* **56**, 795-810 e797 (2021).
8. E. Jahanbakhsh, M. C. Milinkovitch, Modeling convergent scale-by-scale skin color patterning in multiple species of lizards. *Curr Biol* **32**, 5069-5082 e5013 (2022).
9. S. Zakany, M. C. Milinkovitch, Simple Reaction-Diffusion Modeling Predicts Inconspicuous Neighborhood-Dependent Color Subclustering of Lizard Scales. *Phys Rev X* **13**, (2023).
10. M. C. Milinkovitch, Emergence of self-organizational patterning at the mesoscopic scale. *Dev Cell* **56**, 719-721 (2021).
11. D. R. Nolan, A. L. Gower, M. Destrade, R. W. Ogden, J. P. McGarry, A robust anisotropic hyperelastic formulation for the modelling of soft tissue. *J Mech Behav Biomed* **39**, 48-60 (2014).
12. T. Tallinen, J. S. Biggins, L. Mahadevan, Surface Sulci in Squeezed Soft Solids. *Phys Rev Lett* **110**, (2013).
13. J. Teran, S. Blemker, V. N. T. Hing, R. Fedkiw, paper presented at the Proceedings of the 2003 ACM SIGGRAPH/Eurographics symposium on Computer animation, San Diego, California, 2003.
14. P. I. Frazier, A Tutorial on Bayesian Optimization. *arXiv:1807.02811v1*, (2018).
15. D. R. Jones, M. Schonlau, W. J. Welch, Efficient global optimization of expensive black-box functions. *J Global Optim* **13**, 455-492 (1998).

3. Supplementary Videos

SUPPLEMENTARY VIDEO LEGENDS

Supplementary Video 1 - Growth Series (TO-PRO-3) (Fig. 1). Normal development of head scales in the embryonic crocodile.

Supplementary Video 2 - Growth Series (Alizarin red) (Extended Data Figure 1B). Normal development of jaw bones in the embryonic crocodile.

Supplementary Video 3 - EdU labelling. Proliferating (EdU+) cells during head scale development in the embryonic crocodile.

Supplementary Video 4 - 2 ug EGF vs Control (Fig. 2). EGF treatment results in abnormal head scale patterning in the embryonic crocodile.

Supplementary Video 5 - EGF dose comparison (Extended Data Figure 3). EGF treatment effect is dose-dependent, with higher doses resulting in more folding.

Supplementary Video 6 - Tissue layer geometry (Fig. 3). LSFM was used to obtain the precise geometry of different tissue layers.

Supplementary Video 7 - Simulation (Fig. 4). Our mechanical growth simulation recapitulates normal crocodile head scale patterning.

Supplementary Video 8 - Simulation without bony ridges. Bony ridges are required for the proper alignment of the lateral borders of the elongated dorsal scales.

Supplementary Video 9 - Simulation with fully homogeneous growth. Failing to account for the observed reduced skin growth on the dorsal region of the upper jaws prevents the development of properly-elongated scales.

Supplementary Video 10 - Simulation without collagen anisotropy (Supplementary Figure 12). Collagen anisotropy is essential for the normal patterning of crocodile head scales.

Supplementary Video 11 - Simulation (Fig. 4). Our mechanical growth simulation recapitulates abnormal (EGF-induced) crocodile head scale patterning.

Supplementary Video 12 - Simulations (Extended Data Figure 10). Mechanical growth simulations recapitulate all our experimental results, including the transition from labyrinthine to caiman-like head scale patterning.

Supplementary Video 13 - 3D model of the embryonic crocodile head at E64 integrating anatomical elements captured with light-sheet microscopy: bone (beige), teeth (white), dermis (light pink), collagen fibre directions (purple), ISOs (orange), and epidermis (red).
