# Supplementary material for: Self-organized patterning of crocodile head scales by compressive folding
Source: Nature. 2024 Dec 11;637(8045):375–83. doi: 10.1038/s41586-024-08268-1 (PMC11711089; doi:10.1038/s41586-024-08268-1)
Supplement: Supplementary file 2 — Reporting Summary [file 41586_2024_8268_MOESM2_ESM.pdf]

nature portfolio

Corresponding author(s): Michel C. Milinkovitch

Last updated by author(s): October 18, 2024

Reporting Summary

Nature Portfolio wishes to improve the reproducibility of the work that we publish. This form provides structure for consistency and transparency in reporting. For further information on Nature Portfolio policies, see our Editorial Policies and the Editorial Policy Checklist.

Statistics

For all statistical analyses, confirm that the following items are present in the figure legend, table legend, main text, or Methods section.

n/a	Confirmed	
☐	☒	The exact sample size (*n*) for each experimental group/condition, given as a discrete number and unit of measurement
☐	☒	A statement on whether measurements were taken from distinct samples or whether the same sample was measured repeatedly
☐	☒	The statistical test(s) used AND whether they are one- or two-sided<br>*Only common tests should be described solely by name; describe more complex techniques in the Methods section.*
☒	☐	A description of all covariates tested
☒	☐	A description of any assumptions or corrections, such as tests of normality and adjustment for multiple comparisons
☐	☒	A full description of the statistical parameters including central tendency (e.g. means) or other basic estimates (e.g. regression coefficient) AND variation (e.g. standard deviation) or associated estimates of uncertainty (e.g. confidence intervals)
☐	☒	For null hypothesis testing, the test statistic (e.g. *F*, *t*, *r*) with confidence intervals, effect sizes, degrees of freedom and *P* value noted<br>*Give P values as exact values whenever suitable.*
☐	☒	For Bayesian analysis, information on the choice of priors and Markov chain Monte Carlo settings
☐	☒	For hierarchical and complex designs, identification of the appropriate level for tests and full reporting of outcomes
☒	☐	Estimates of effect sizes (e.g. Cohen's *d*, Pearson's *r*), indicating how they were calculated

Our web collection on statistics for biologists contains articles on many of the points above.

Software and code

Policy information about availability of computer code

Data collection	ImspectorPro v7.5.4 (Miltenyi Biotech), Optics11 nanoindenter v3.4.1 (Optics 11).
Data analysis	Imaris v9.9.1 (Oxford Instruments); ImageJ; Redshift engine of Houdini v20.5 (SideFX) and Unreal Engine v5.4 (Epic Games); Prism9 (GraphPad); MATLAB R2021a (MathWorks); Meshlab v2023.12., CUDA v11.7. The executables and original code used for the numerical simulations presented here are provided as a Code Ocean capsule at https://codeocean.com/capsule/7106541/tree/v2 and https://doi.org/10.24433/CO.2472398.v2

For manuscripts utilizing custom algorithms or software that are central to the research but not yet described in published literature, software must be made available to editors and reviewers. We strongly encourage code deposition in a community repository (e.g. GitHub). See the Nature Portfolio guidelines for submitting code & software for further information.

Data

Policy information about availability of data

All manuscripts must include a data availability statement. This statement should provide the following information, where applicable:

- Accession codes, unique identifiers, or web links for publicly available datasets
- A description of any restrictions on data availability
- For clinical datasets or third party data, please ensure that the statement adheres to our policy

Provide your data availability statement here.

Research involving human participants, their data, or biological material

Policy information about studies with human participants or human data. See also policy information about sex, gender (identity/presentation), and sexual orientation and race, ethnicity and racism.

Reporting on sex and gender	N/A
Reporting on race, ethnicity, or other socially relevant groupings	N/A
Population characteristics	N/A
Recruitment	N/A
Ethics oversight	N/A

Note that full information on the approval of the study protocol must also be provided in the manuscript.

Field-specific reporting

Please select the one below that is the best fit for your research. If you are not sure, read the appropriate sections before making your selection.

☒ Life sciences ☐ Behavioural & social sciences ☐ Ecological, evolutionary & environmental sciences

For a reference copy of the document with all sections, see nature.com/documents/nr-reporting-summary-flat.pdf

Life sciences study design

All studies must disclose on these points even when the disclosure is negative.

Sample size	The number of individual embryos and juveniles are listed in the relevant figure legends and supplementary data tables. These individuals were necessary to establish the robustness of the treatment, establish the existence of dose effect, and establish significance. Sample size was sufficient to infer significance of the results.
Data exclusions	We did not exclude data.
Replication	All replicates of EGF treatment are listed in Suppl. Data Tables 1 & 2 and shown in Suppl. Data Figs. 4 and 6-9. All samples used for LSFM are listed in Suppl. Data Table 5.
Randomization	Embryos are collected opportunistically and are considered as random representatives of the species.
Blinding	N/A

Reporting for specific materials, systems and methods

We require information from authors about some types of materials, experimental systems and methods used in many studies. Here, indicate whether each material, system or method listed is relevant to your study. If you are not sure if a list item applies to your research, read the appropriate section before selecting a response.

Materials & experimental systems

n/a	Involved in the study	
☒	☐	Antibodies
☒	☐	Eukaryotic cell lines
☒	☐	Palaeontology and archaeology
☐	☒	Animals and other organisms
☒	☐	Clinical data
☒	☐	Dual use research of concern
☒	☐	Plants

Methods

n/a	Involved in the study	
☒	☐	ChIP-seq
☒	☐	Flow cytometry
☒	☐	MRI-based neuroimaging

Animals and other research organisms

Policy information about studies involving animals; ARRIVE guidelines recommended for reporting animal research, and Sex and Gender in Research

Laboratory animals	Nile crocodile embryos and juveniles. Maintenance of, and experiments with, crocodile embryos and juveniles were approved by the Geneva Canton ethical regulation authority (authorisation GE10619B) and performed according to Swiss law. There is no specific strain.
Wild animals	No wild animal were used.
Reporting on sex	N/A (we study here a sexually monomorphic trait).
Field-collected samples	Eggs were note collected in the field but in a captive breeding farm, and were incubated in the laboratory.
Ethics oversight	Maintenance of, and experiments with, crocodile embryos and juveniles were approved by the Geneva Canton ethical regulation authority (authorisation GE10619B) and performed according to Swiss law.

Note that full information on the approval of the study protocol must also be provided in the manuscript.

Plants

Seed stocks	N/A
Novel plant genotypes	N/A
Authentication	N/A
